# The efficacy of fiber-supplemented enteral nutrition in critically ill patients: a systematic review and meta-analysis of randomized controlled trials with trial sequential analysis

**DOI:** 10.1186/s13054-024-05128-2

**Published:** 2024-11-07

**Authors:** Jana Larissa Koch, Charles Chin Han Lew, Felix Kork, Alexander Koch, Christian Stoppe, Daren K. Heyland, Ellen Dresen, Zheng-Yii Lee, Aileen Hill

**Affiliations:** 1 Medical Faculty, RWTH Aachen University, Aachen, Germany; 2 Department of Anaesthesiology, University Hospital RWTH Aachen, Pauwelsstraße 30, 52074 Aachen, Germany; 3 Department of Dietetics and Nutrition, Ng Teng Fong General Hospital, Singapore, Singapore; 4 Faculty of Health and Social Sciences, Singapore Institute of Technology, Singapore, Singapore; 5 Department of Gastroenterology, Metabolic Diseases and Internal Intensive Care Medicine, University Hospital RWTH Aachen, Aachen, Germany; 6 Department of Anaesthesiology, Intensive Care, Emergency and Pain Medicine, University Hospital Würzburg, Würzburg, Germany; 7 Department of Cardiac Anaesthesiology and Intensive Care Medicine, Charité Berlin, Berlin, Germany; 8 Department of Critical Care Medicine, Queen’s University, Kingston, ON Canada; 9 Department of Anaesthesiology, University of Malaya, Kuala Lumpur, Malaysia; 10 Department of Intensive Care Medicine, University Hospital RWTH Aachen, Aachen, Germany

**Keywords:** Medical nutrition therapy, Fiber, Enteral nutrition, Critical care, Systematic review, Meta-analysis, Trial sequential analysis

## Abstract

**Background:**

Evidence on the benefits of fiber-supplemented enteral nutrition (EN) in critically ill patients is inconsistent, and critical care nutrition guidelines lack recommendations based on high-quality evidence. This systematic review and meta-analysis (SRMA) aims to provide a current synthesis of the literature on this topic.

**Methods:**

For this SRMA of randomized controlled trials (RCT), electronic databases (MEDLINE, EMBASE, CENTRAL) were searched systematically from inception to January 2024 and updated in June 2024. Trials investigating clinical effects of fiber-supplemented EN versus placebo or usual care in adult critically ill patients were selected. Two independent reviewers extracted data and assessed the risk of bias of the included studies. Random-effect meta-analysis and trial sequential analysis (TSA) were conducted. The primary outcome was overall mortality, and one of the secondary outcomes was diarrhea incidence. Subgroup analyses were also performed for both outcomes.

**Results:**

Twenty studies with 1405 critically ill patients were included. In conventional meta-analysis, fiber-supplemented EN was associated with a significant reduction of overall mortality (RR 0.66, 95% CI 0.47, 0.92, *p* = 0.01, I^2^ = 0%; 12 studies) and diarrhea incidence (RR 0.70, 95% CI 0.51, 0.96, *p* = 0.03, I^2^ = 51%; 11 studies). However, both outcomes were assessed to have very serious risk of bias, and, according to TSA, a type-1 error cannot be ruled out. No subgroup differences were found for the primary outcome.

**Conclusion:**

Very low-certainty evidence suggests that fiber-supplemented EN has clinical benefits. High-quality multicenter RCTs with large sample sizes are needed to substantiate any firm recommendation for its routine use in this group of patients.

*PROSPERO registration number:* CRD42023492829.

**Supplementary Information:**

The online version contains supplementary material available at 10.1186/s13054-024-05128-2.

## Introduction

Critical illness is frequently associated with severe changes in gut function, metabolism and induces a catabolic stress state, often leading to malnutrition and compromised immune function [1–3].

Enteral nutrition (EN) is the preferred route of medical nutrition therapy for critically ill patients [4]. However, a common challenge among critically ill patients is enteral feeding intolerance with a prevalence of up to 75% [5, 6], leading to inadequate nutrient delivery and gastrointestinal (GI) symptoms like constipation or diarrhea [7, 8]. Therefore, inexpensive and safe interventions would be needed to manage this challenge.

Dietary fiber (DF) is a type of carbohydrate that is not/only partially hydrolyzed or absorbed in the human small intestine [9]. DF has been shown to provide various benefits in disease prevention among healthy individuals, including, among other benefits, reduced risk of mortality, type-2 diabetes, and cardiovascular disease [10–13]. A recent narrative review suggested considerable benefits from DF in critically ill patients, attributed to its functions in maintaining gut barrier integrity, modulating immune responses, supporting the gut microbiome, and contributing to systemic anti-inflammatory responses [9]. Formulations containing DF have been introduced attempting to improve GI tolerance of EN in critically ill patients [14]. However, existing trials about DF for critically ill patients yield inconsistent results [13], and there is a lack of up-to-date, high-quality systematic reviews of randomized controlled trials (RCTs) with meta-analysis (SRMA). Consequently, the routine use of DF in intensive care unit (ICU) settings remains unclear. While the American Society for Parenteral and Enteral Nutrition (ASPEN) and the Society of Critical Care Medicine (SCCM) recommend considering the routine use of fermentable soluble DF supplements in stable medical and surgical ICU patients, they advise against the routine use of mixed soluble and insoluble DFs due to concerns about bowel ischemia and dysmotility [15]. Conversely, the European Society for Clinical Nutrition and Metabolism (ESPEN) guidelines do not address the use of DF in the ICU [16].

Prior systematic reviews examining the effects of fiber-supplemented EN in adult critically ill patients [17–19], have included non-RCTs [20–24]. Furthermore, none of the preceding meta-analyses applied trial sequential analysis (TSA), limiting accurate assessment of type-1 and –2 error within the meta-analyses [25]. TSA helps in assessing the robustness of results and minimizes the risk of distortion due to random errors [26].

Therefore, we conducted a SRMA of RCTs and included TSA to generate a higher quality and more precise estimate regarding the efficacy of fiber-supplemented EN in critically ill patients. We also performed GRADE certainty of evidence assessment, thereby enhancing the conclusiveness and reliability of our findings.

## Methods

This SRMA was performed in accordance with the 2020 Preferred Reporting Items for Systematic Reviews and Meta-Analyses (PRISMA) statement [27]. The PRISMA 2020 checklist is shown in Additional file 1: Part 1. The protocol was registered in PROSPERO (CRD42023492829).

### Eligibility criteria

RCTs of adult (age ≥ 16 years) critically ill patients (defined as admission to the ICU, or if uncertain, a mortality rate of ≥ 5% in the control group or mechanical ventilation at the study inclusion) that compared fiber-supplemented EN with placebo or usual care and reported at least one clinical or GI outcome were included. Pseudorandomized trials and studies that investigated the effects of synbiotics were excluded. Studies among patients with elective or cancer surgery or studies only reporting laboratory, metabolic or nutritional outcomes were also excluded.

### Outcomes

The primary outcome was overall mortality. When multiple mortality endpoints were reported in a trial, the data was included in the following order of preference: 28-/30-day mortality > hospital mortality > ICU mortality > other mortality. Secondary outcomes included diarrhea, other GI complications, ICU and hospital length of stay (LOS), duration of mechanical ventilation (MV), infectious complications, metabolic (blood glucose, triglycerides) and nutritional (e.g. tolerated feeding volumes, time to reach energy targets) outcomes.

### Information sources and search strategies

MEDLINE, EMBASE, and CENTRAL (Cochrane Database of Systematic Reviews and the Cochrane Central Register of Controlled Trials) were searched through OVID on January 11, 2024, for all relevant RCTs published from database inception to January 09, 2024. No language restrictions were made. The reference lists of previous SRMAs were also reviewed and ClinicalTrials.gov was searched for ongoing studies. The detailed search strategies are presented in Additional file 1: Part 1. The search was repeated on June 10, 2024, to identify potential studies published after the initial search.

### Study selection

Search results were exported into Covidence (Veritas Health Innovation, Melbourne, Australia) for screening and removal of duplicates. The article titles and abstracts were screened by two independent reviewers (JK and AH). Full texts of potential eligible trials were retrieved and reviewed independently by the same two reviewers. Disagreements were discussed with a third author (ZYL).

### Data collection

Data from eligible trials were extracted independently by two reviewers (JK and AH). Abstracted data including study and patient characteristics, funding sources, feeding information, clinical, metabolic and nutritional outcomes, diarrhea, and adverse events are summarized in Additional file 1: Tables S1–S7. For studies that reported median (Q1–Q3) for continuous outcomes, authors were contacted to obtain the mean and standard deviation (SD). If means and SDs were unavailable, those outcomes were excluded from the meta-analysis. No assumption or data conversion was made if the information could not be obtained.

### Study quality and risk-of-bias assessment

The quality of the included trials was evaluated independently by two authors using the Canadian Critical Care Nutrition (CCN) Methodological Quality System (JK and ZYL) and the Cochrane Risk of Bias 2 tool (ROB2) (JK and ZYL) [28]. The overall ROB2 assessment was categorized as high risk-of-bias, some concerns, or low risk of bias. The risk of bias traffic light and summary plots were generated by the risk-of-bias visualization (robvis) tool [29]. The CCN Methodological Quality System is used in CCN systematic reviews and allows quality comparisons across topics and time [30]. The methodologic score ranges from 0 to 14 points, where a higher score indicates higher study quality.

### Data analysis

All analyses were performed with a random effects model using RevMan 5.4 (Cochrane IMS, Oxford, UK). For dichotomized outcomes, the pooled risk ratio (RR) was estimated by the DerSimonian and Laird random effect meta-analysis. For continuous outcomes, the random effect mean difference (MD) was estimated. Heterogeneity was quantified by the I^2^ measure. The result of the meta-analysis was presented in the forest plot generated by RevMan. Presence of potential publication bias was evaluated by funnel plots for overall outcomes. Egger’s test for funnel plot asymmetry was performed by using the metafor package in RStudio (version 2023.12.1) if ≥ 10 studies were included in a meta-analysis [31]. All estimates were provided with 95% confidence intervals (CI). A *p*-value < 0.05 was considered statistically significant.

### Subgroup analyses

Subgroup analyses were performed for overall mortality and diarrhea incidence. The following a priori subgroup analyses were conducted: (1) publication date before 2000 versus after 2000, (2a) fermentable versus non-fermentable versus mixed fiber, (2b) viscous versus non-viscous versus mixed fiber, (2c) soluble versus insoluble versus mixed fiber (based on the classification provided by Gill et al [10]), (3) daily fiber dose < 20 g versus  ≥ 20 g, (4) average age < 50 years versus  ≥ 50 years, (5) average APACHE II score < 17 versus  ≥ 17, (6) medical versus surgical versus mixed ICU, (7) intervention start ≤ 24 h versus  ≤ 48 h, and (8) minimum duration of intervention < 6 days versus ≥ 6 days. All cut-offs for continuous data were based on the median. The calculation of the daily fiber doses is detailed in Additional file, Table S12. For the age subgroup, an a priori planned cut-off of 65 years was adjusted to the median of 50 years post-hoc to provide a more even distribution of studies while maintaining validity for the comparison of younger versus older study population. Subgroup analyses were not performed for the following pre-planned domains as data were not sufficiently available: patients with abdominal surgery versus others, and patients with shock/vasopressor use versus others. Additionally, no subgroup analysis on study quality was conducted, as none of the included studies had a low risk of bias. The following post-hoc subgroup analyses were added: (9) Co-intervention with immunonutrition versus DF only, (10) funding source of the trial (industry vs. non-industry), and (11) standard formula versus non-standard formula in the control group. All results of the subgroup analyses were not adjusted for multiplicity. Hence, they should be viewed as hypothesis-generating.

### Trial sequential analysis

To control for type-1 and type-2 errors [25], TSA was performed for the following outcomes: overall mortality, diarrhea incidence, ICU LOS and hospital LOS. All TSA were performed using the TSA software (0.9.5.10 Beta, The Copenhagen Trial Unit, Denmark) with the pre-specified parameters detailed in Additional file 1: Part 1.

### Certainty of evidence

The Grading of Recommendations Assessment, Development, and Evaluation (GRADE) system was used to rate the certainty of evidence for outcomes analyzed with TSA [32]. The certainty of the evidence was rated as high, moderate, low, and very low by considering the risk of bias, inconsistency, indirectness, imprecision, and publication bias. GRADEpro was used to prepare the GRADE evidence profile table [33].

### Deviations from the original protocol

While diarrhea was initially considered as secondary outcome alongside other GI complications in the original protocol, we decided to place particular emphasis on it in our analyses for two main reasons. First, diarrhea is a highly prevalent symptom associated with enteral nutrition, with prevalence rates up to 41%, and it significantly impacts patient dignity and morbidity, contributing to issues such as electrolyte imbalances and increased infection risk [34]. Additionally, the incidence of diarrhea was the second most frequently reported outcome in the included studies, after mortality, and provided substantially more data than other secondary outcomes.

Subgroup analyses were performed only for mortality and diarrhea incidence, which was not explicitly specified in the original protocol. This decision was made, as these outcomes were the most clinically relevant and had the most data available, thereby avoiding excessive analyses with limited data.

## Results

### Study selection

**Fig. 1:**
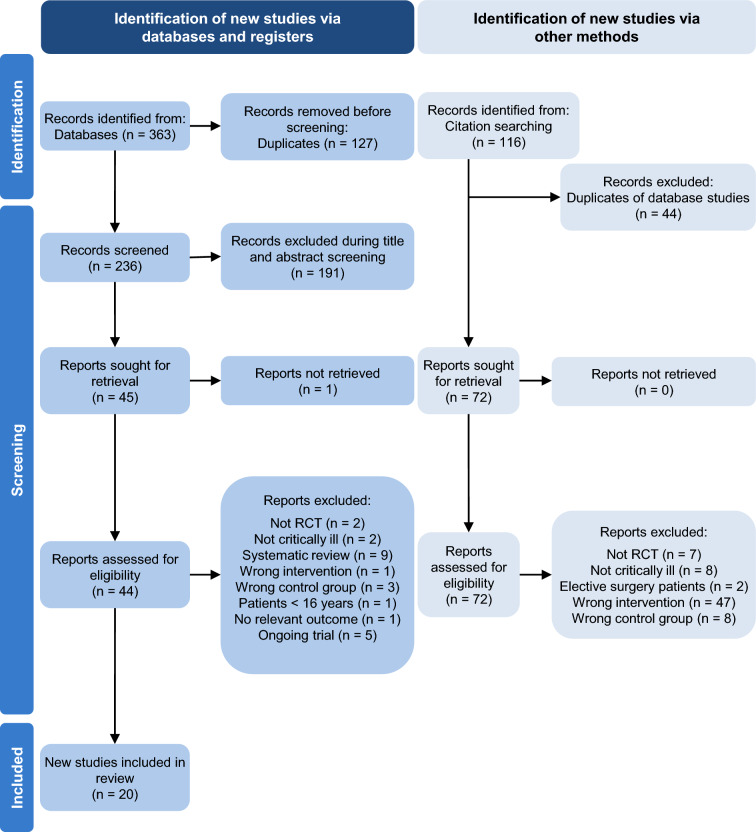
PRISMA Flowchart. One report was not retrieved because neither the abstract nor the full text was available

The search identified a total of 363 records from the databases. After removing duplicates, 236 abstracts were screened and of these, 44 full-text articles were assessed for eligibility. Twenty trials with a total of 1405 patients published between 1988 and 2021 were included. The detailed study selection flow is presented in Fig. 1. Five ongoing or unpublished related trials were identified (Additional file 1: Table S8). The excluded trials with the reason for exclusion are listed in Additional file 1: Table S9.

### Risk of bias and study quality

The CCN score of the studies ranged from 2 to 10, with a median score of 6 (Additional file 1: Table S10). The ROB2 plots are presented in Additional file 1: Fig. S1. None of the included studies had an overall rating of low risk of bias. In 12 studies that reported mortality outcomes, 9/12 (75%) were at high risk of bias and 3/12 (25%) had some concerns. The biases mainly arose from the randomization process, deviations from intended interventions and selection of the reported results.

### Study characteristics

Included studies and patient characteristics are summarized in Additional file 1: Table S1. The sample sizes ranged from 20 to 220 (median: 56). Only one study was a multi-center trial [35]. Seven studies enrolled mixed medical and surgical patients [35–41], three included only medical [42–44] and two included only surgical ICU patients [45, 46]. One study included trauma and septic patients with stress diabetes [47], two studies included patients with severe acute pancreatitis [48, 49], two included patients with multi-organ trauma [50, 51], one included patients with traumatic brain injury and hemorrhagic stroke [52], and diseases or ICU admission category were unclear in two studies [53, 54]. In all reviewed studies, EN was administered via feeding tubes. The majority (n = 16) compared fiber-supplemented EN with standard EN [36, 38–45, 48–54]. Two studies added immunomodulating components in the intervention group: one study included arginine and antioxidants (vitamins E and C) [35], and another added glutamine [51], while the control groups lacked these components. Conversely, two studies compared fiber-supplemented EN against control EN formulations that either contained glutamine, arginine, and linolenic acid [46] or were high in protein [46, 47]. The intervention groups in these studies did not receive these additional components. One study administered glutamine in both groups [37], and another provided high-protein formulas in both groups [35]. A detailed summary of the interventions is outlined in Additional file 1: Table S3*,* and all relevant outcomes are summarized in Additional file 1: Tables S4–S6.

### Overall mortality

**Fig. 2:**
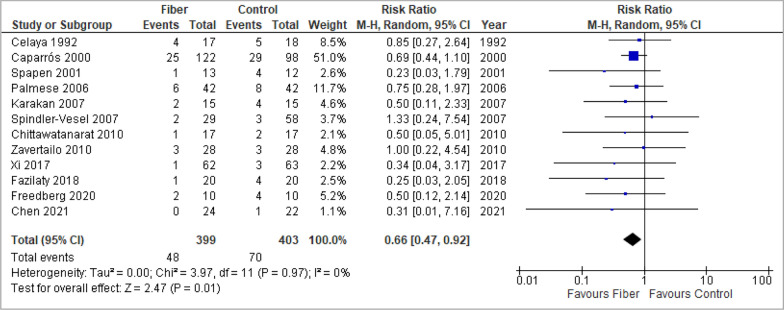
Meta-analysis of overall mortality

In statistically aggregated data from twelve studies, a significant effect of fiber-supplemented EN on overall mortality was observed (RR 0.66, 95% CI 0.47, 0.92, *p* = 0.01, I^2^ = 0%) (Fig. 2).

No evidence of funnel plot asymmetry was detected in the overall analysis (*p* = 0.14, Additional file 1: Fig. S9).

There was no evidence for subgroup differences in any of the subgroup analyses. The results of all subgroup analyses are summarized in Additional file 1: Table S11 and visualized in Additional file 1: Fig. S2.

### Diarrhea

**Fig. 3:**
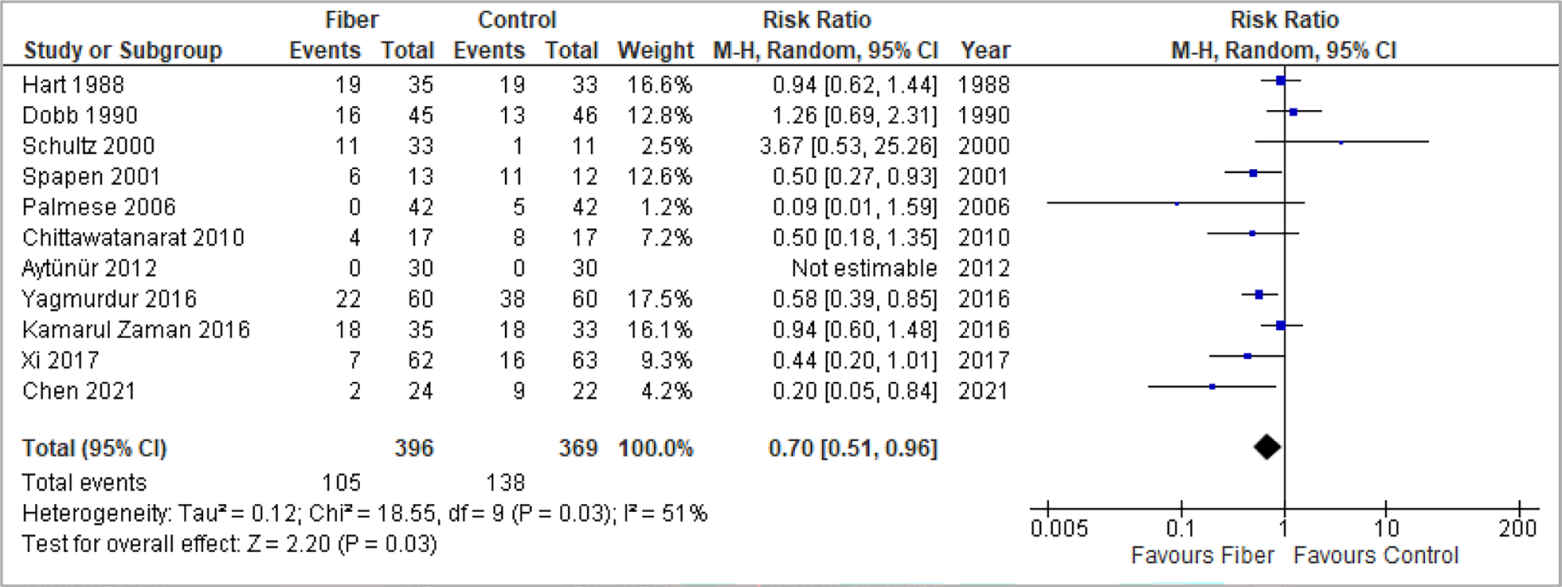
Meta-analysis of diarrhea incidence

Fiber-supplemented EN was associated with a significant reduction of the diarrhea incidence (RR 0.70, 95% CI 0.51, 0.96, *p* = 0.03, I^2^ = 51%; 11 studies) (Fig. 3) and no evidence of funnel plot asymmetry was detected in the overall analysis (*p* = 0.41, Additional file 1: Fig. S10a).

Additionally, a significant benefit of fiber-supplemented EN in meta-analysis of diarrhea scores according to the Hart and Dobb diarrhea scale was found (MD -2.77, 95% CI − 4.10, − 1.45, *p* < 0.0001, I^2^ = 0%; 3 studies; Additional file 1: Fig. S4). Visual inspection of the funnel plot found no evidence of asymmetry (Additional file 1: Fig. S10b).

In the subgroup analyses, studies published after 2000 indicated a significant reduction of diarrhea events through fiber-supplemented EN (RR 0.59, 95% CI 0.40, 0.85, *p* = 0.005, I^2^ = 43%; 9 studies), a result that was not supported by studies published before 2000 (RR 1.04, 95% CI 0.73, 1.46, *p* = 0.84, I^2^ = 0%; 2 studies) (test for subgroup differences: *p* = 0.03, I^2^ = 79.4%). Providing fiber-supplemented EN in sicker patients (APACHE II ≥ 17 compared to APACHE < 17 and unclear APACHE score) and in medical ICUs (compared to surgical ICUs, mixed ICUs and unclear admission type) seemed to be associated with a significant reduction of diarrhea incidence (tests for subgroup differences: *p* = 0.01 and *p* = 0.02, respectively). No evidence for subgroup differences was found in other subgroup analyses, as summarized in Additional file 1: Table S11 and visualized in Additional file 1: Fig. S3.

### Other gastrointestinal complications

Four studies reported the overall incidence of GI complications (n = 315). No significant difference was found between groups (RR 0.75, 95% CI 0.49, 1.15, *p* = 0.19, I^2^ = 58%) (Additional file 1: Fig. S5a). There was also no significant difference between groups for the incidence of abdominal distension, vomiting, regurgitation and GI bleeding (Additional file 1: Fig. S5b–S5e). However, pooled data from six studies showed a significant benefit of fiber-supplemented EN for the incidence of constipation (RR 0.33, 95% CI 0.19, 0.58, *p* = 0.0001, I^2^ = 0%) (Additional file 1: Fig. S5f). Visual inspection of the funnel plots found no evidence of asymmetry (Additional file 1: Fig. S11).

### Length of ICU and hospital stay

**Fig. 4:**
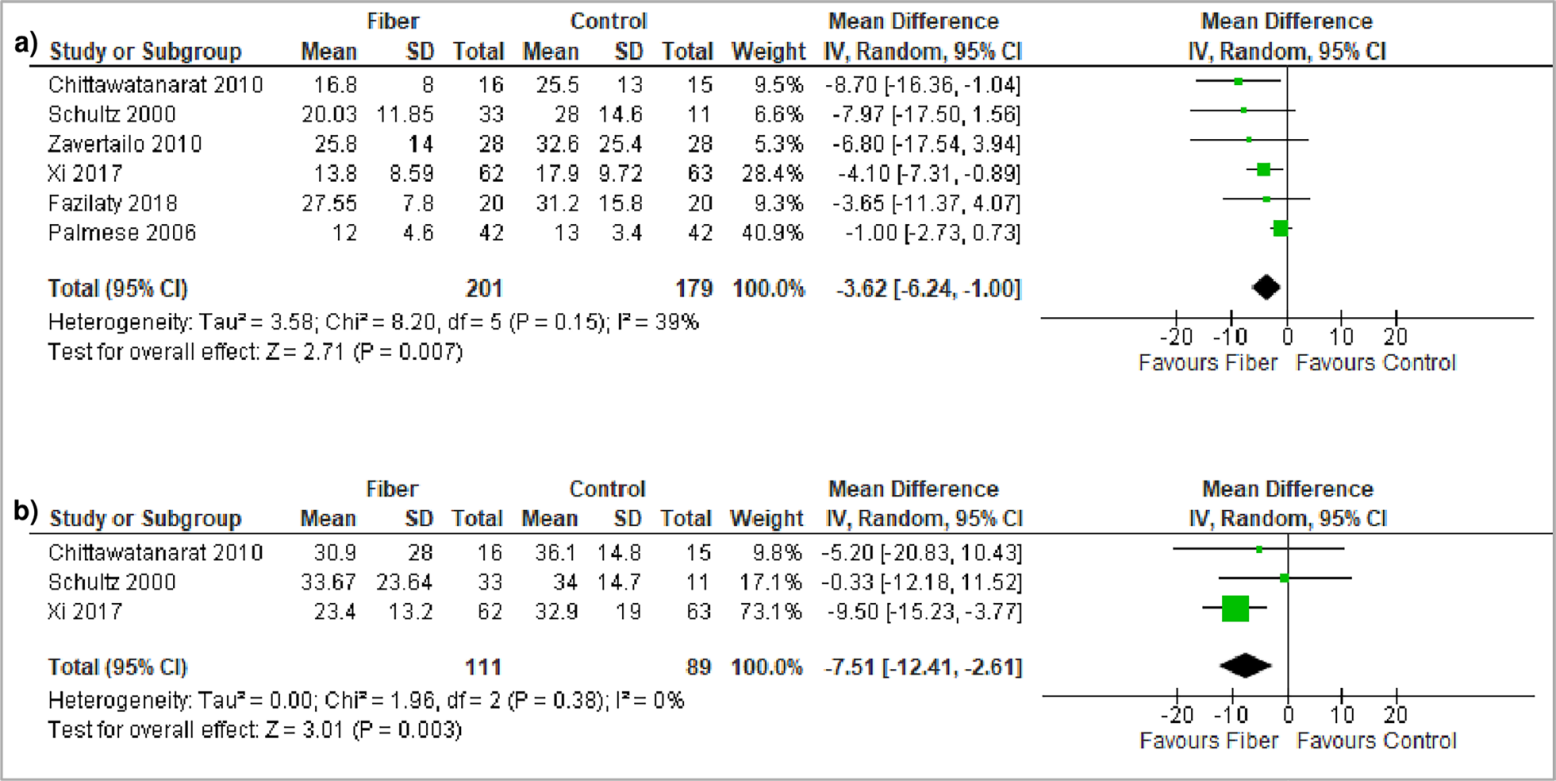
Meta-analysis of **a** ICU LOS and **b** Hospital LOS

Fiber-supplemented EN was associated with a significantly reduced ICU (MD -3.62, 95% CI − 6.24, − 1.00, *p* = 0.007, I^2^ = 39%; 6 studies) and hospital LOS (MD − 7.51, 95% CI − 12.41, − 2.61, *p* = 0.003, I^2^ = 0%; 3 studies) (Fig. 4). Visual inspection of the funnel plots found no evidence of asymmetry (Additional file 1: Fig. S12 and S13).

### Infectious complications

No association was observed between fiber-supplemented EN and the overall incidence of infectious complications (RR 0.65, 95% CI 0.37, 1.14, *p* = 0.13, I^2^ = 0%; 3 studies) (Additional file 1: Fig. S6a). There was no significant evidence for influence on the incidence of pneumonia, urinary tract infection, intra-abdominal infection, sepsis, vascular infection, wound infection and bacteremia (Additional file 1: Fig. S6b–S6h). Visual inspection of the funnel plots found no evidence of asymmetry (Additional file 1: Fig. S14).

### Duration of mechanical ventilation

In three studies reporting the duration of MV, there was no significant difference between groups (MD 0.02, 95% CI − 2.30, 2.34, *p* = 0.98, I^2^ = 39%) (Additional file 1: Fig. S7) and visual inspection of the funnel plot found no evidence of asymmetry (Additional file 1: Fig. S15).

### Metabolic outcomes

One study reported episodes of hypoglycemia, finding a significant benefit of fiber-supplemented EN [54]. Two studies presented blood glucose [47, 48] and one study serum triglyceride levels [47] (Additional file 1: Table S5). Due to heterogeneous timing and units of measurement, the data were unsuitable for a pooled meta-analysis.

### Nutritional outcomes

Gastric residual volume, assessed in three studies, showed no significant differences at various timepoints [44, 46, 53]. Five studies [35, 36, 48, 50, 52] measured the administered caloric intake, with one indicating a benefit for fiber supplementation on the mean overall energy intake [48] and one on the intake on specific days [52]. Tolerated feeding volumes were investigated in five studies [38–40, 44, 46], with one revealing significantly greater volumes for the intervention group on specific days [40] and one for the mean daily volume ratio [44] (Additional file 1: Table S5). Due to variability in timing and units of measurement, data was not aggregated for these outcomes.

For meta-analysis, the time to reach energy targets was pooled from two studies, revealing a beneficial effect of fiber-supplemented EN (MD − 2.25, 95% CI − 4.16, − 0.33, *p* = 0.02, I^2^ = 53%) (Additional file 1: Fig. S8). Visual inspection of the funnel plot found no evidence of asymmetry (Additional file 1: Fig. S16).

### Adverse events

Three studies reported the incidence of adverse events, but they were not pooled due to inconsistency in definitions. One study investigated GI adverse events and observed no significant difference between groups [51]. Another study found no differences between groups for the incidence or severity of adverse health events [42]. One study found no adverse events related to DF but did not provide a specific definition and did not compare these findings to the control group [48] (Additional file 1: Table S7).

### Trial sequential analysis

**Fig. 5:**
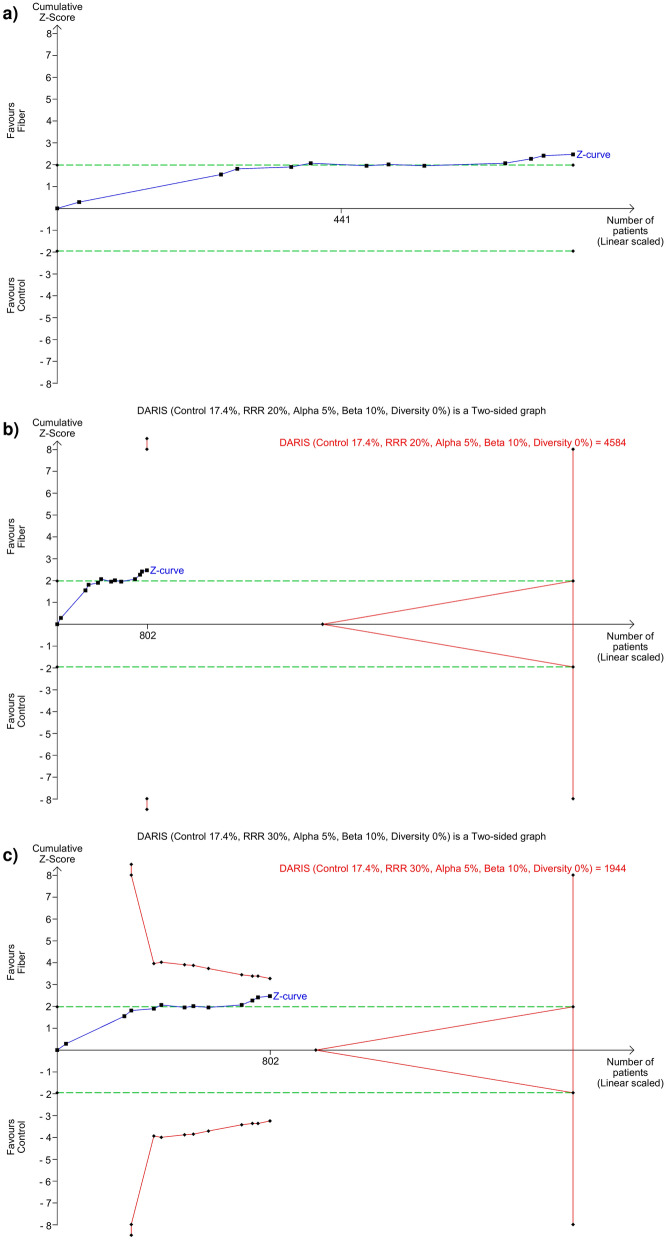
Trial Sequential Analysis (TSA) for overall mortality. **a** RRR = 10%, **b** RRR = 20%, **c** RRR = 30%. *DARIS* diversity-adjusted required information size; *RRR* relative risk reduction. The Z curve in blue measures the treatment effect (pooled relative risk). The parallel lines in green are the boundaries of conventional meta-analysis (alpha 5%). The red lines, located outside the parallel lines, are the boundaries of benefit and harm. These are boundaries of conventional meta-analysis adjusted for between-trial heterogeneity and multiple statistical testing (TSA boundaries). A treatment effect outside the TSA boundaries of benefit/harm indicates reliable evidence for a pre-defined magnitude of treatment effect, and a treatment effect within the futility zone (the triangle between the parallel lines) indicates that there is reliable evidence of an absence of a pre-defined magnitude of treatment effect

Results of TSA are summarized in Table 1 and presented in Fig. 5 and Additional file 1: Fig. S17–S19. TSA revealed that the current systematic review did not achieve the required information size (RIS) to detect the pre-specified effect sizes for overall mortality, diarrhea incidence, ICU and hospital LOS. In addition, for all outcomes, the pooled RR crossed the boundaries of conventional meta-analysis (i.e. significant) but did not cross (i.e. not significant) the trial sequential boundaries or the futility boundaries. This suggests the possibility of false positive results, indicating that more adequately designed studies are required to accrue sufficient information to confirm any benefits and justify the routine use of fiber-supplemented EN in critically ill patients. Post-hoc, additional plausible larger effect sizes were tested, and the interpretations were similar (Table 1).

**Table 1:** Summary of results of TSA

Effect size	Incidence, or variance	I^2^ (%)	D^2^ (%)	RIS	% of RIS attained	Pooled effect (TSA adjusted 95% CI)	Z-curve passed the conventional boundaries?	Z-curve passed the TSA boundaries?	Z-curve passed the futility boundaries?
*Overall mortality (12 studies, n* = *802)*									
RRR: 10.0%	17.4%	0	0	19,155	4.19	NA	Yes	No	No
RRR: 20.0%*	17.4%	0	0	4584	17.5	0.66 (0.17, 2.55)	Yes	No	No
RRR: 30.0%*	17.4%	0	0	1944	41.3	0.66 (0.38, 1.14)	Yes	No	No
*Intensive care unit length of stay (6 studies, n* = *380)*									
MIREDIF 1 day	51.1	39	69.9	7119	18.7	− 3.62 (− 14.30, 7.06)	Yes	No	No
MIREDIF 2 days*	51.1	39	69.9	1781	21.3	− 3.62 (− 9.80, 2.56)	Yes	No	No
MIREDIF 3 days*	51.1	39	69.9	792	48.0	− 3.62 (− 7.73, 0.49)	Yes	No	No
*Hospital length of stay (3 studies, n* = *200)*									
MIREDIF 1 day	312.2	0	0	13,122	1.5	NA	Yes	No	No
MIREDIF 2 days*	312.2	0	0	3281	3.1	− 7.51 (− 27.50, 12.48)	Yes	No	No
MIREDIF 3 days*	312.2	0	0	1459	13.7	− 7.51 (− 27.50, 12.48)	Yes	No	No
*Diarrhea incidence (11 studies, n* = *765)*									
RRR: 15.0%*	37.4	51	60.5	7644	10.0	0.70 (0.20, 2.49)	Yes	No	No
RRR: 25.0%	37.4	51	60.5	2678	28.6	0.70 (0.38, 1.29)	Yes	No	No
RRR: 35.0%*	37.4	51	60.5	1325	57.7	0.70 (0.45, 1.09)	Yes	No	No

*TSA* trial sequential analysis, *I*^2^ Between-trial heterogeneity, *D*^2^ diversity-estimate, *RRR* relative risk reduction, *MIREDIF* minimally relevant difference, *RIS* required information size, *NA* not applicable, *CI* confidence interval

*Post-hoc sensitivity analyses

### GRADE certainty of the evidence

The overall certainty of evidence using GRADE was rated as very low for all examined outcomes, implicating that the true effect is likely to be substantially different from the estimated effect (Table 2). The level of evidence was mainly downgraded due to very serious risk of bias and serious imprecision.

**Table 2:** GRADE certainty assessment and summary of findings table

Certainty assessment							Summary of findings				
Participants (studies) follow-up	Risk of bias	Inconsistency	Indirectness	Imprecision	Publication bias	Overall certainty of evidence	Study event rates (%)		Relative effect (95% CI)	Anticipated absolute effects	
							With control	With fiber		Risk with control	Risk difference with fiber
*Overall mortality*											
802 (12 RCTs)	Very serious^a^	Not serious	Not serious	Serious^b^	None	⨁◯◯◯ Very low	70/403 (17.4%)	48/399 (12.0%)	RR 0.66 (0.47, 0.92)	174 per 1000	59 fewer per 1000 (from 92 to 14 fewer)
*Diarrhea incidence*											
765 (11 RCTs)	Very serious^c^	Serious^d^	Not serious	Serious^b^	None	⨁◯◯◯ Very low	138/369 (37.4%)	105/396 (26.5%)	RR 0.70 (0.51, 0.96)	374 per 1000	112 fewer per 1000 (from 183 to 15 fewer)
*ICU LOS*											
380 (6 RCTs)	Very serious^e^	Not serious	Not serious	Serious^b^	None	⨁◯◯◯ Very low	179	201	–	The mean ICU LOS was 0	MD 3.62 lower (6.24 lower to 1 lower)
*Hospital LOS*											
200 (3 RCTs)	Very serious^f^	Not serious	Not serious	Serious^b^	None	⨁◯◯◯ Very low	89	111	–	The mean hospital LOS was 0	MD 7.51 lower (12.41 lower to 2.61 lower)

*CI* confidence interval, *MD* mean difference, *RR* risk ratio

^a^3/12 studies had some concerns and 9/12 studies had high risk of bias

^b^Wide trial sequential analysis adjusted confidence interval

^c^1/11 studies had some concerns and 10/11 studies had high risk of bias

^d^The overall heterogeneity is I^2 =^ 51%

^e^1/6 studies had some concerns and 5/6 studies had high risk of bias

^f^All included trials had high risk of bias

## Discussion

### Summary of main findings

Overall, this SRMA of 20 RCTs found very low-certainty evidence suggesting the benefits of fiber-supplemented EN in critically ill patients. Although the latter indicated a potential improvement in clinical and diarrheal outcomes, TSA suggested that the accrued information size is insufficient, and more trials are needed to confirm these benefits. Furthermore, the overall certainty of evidence was compromised by a serious risk of bias in the trials.

### Interpretation of the results in the context of other evidence

One SRMA by Cara et al. in 2021 assessed the safety of EN with DF based on 19 studies, including RCTs, retrospective cohort studies, case reports and case series [17]. They found no significant effects on diarrheal events, other GI complications, mortality, or ICU and hospital LOS.

Another SRMA by Liu et al. from 2022 included 20 RCTs and one cohort study, investigating interventions with fiber, probiotics or synbiotics. Liu et al. revealed no significant impact of DF on all clinical outcomes in fiber-only studies [18].

The most recent SRMA from the same group from 2023 included 13 RCTs [19], although one of the included studies was a pseudorandomized trial [55]. They concluded that DF might (or might not) reduce mortality, diarrhea, other GI complications, ICU and hospital LOS and the time to reach full EN.

Given our meticulous search strategy, which was significantly more thorough in both scope and detail, we were able to include a larger number of RCTs than previous SRMAs [36, 37, 47, 50, 52, 53]. Nevertheless, all studies were of low quality and the information size is insufficient to draw definitive conclusions regarding the benefits (or harms) of fiber-supplemented EN.

### Impact of the results on clinical practice and future research

The complexity of clinical decision-making regarding the routine administration of DF in critically ill patients is reflected by the absence of clear guidance in major nutritional guidelines. ESPEN guidelines do not address the use of DF in the ICU at all [16]. In contrast, ASPEN recommends caution with the use of insoluble fiber [15] due to historical concerns about bowel obstruction, a potential adverse event documented in two case reports from 1990 that investigated five surgical and trauma patients receiving insoluble fiber [23, 24]. The studies in our meta-analyses are small and of low quality, and adverse effects of DF, especially bowel obstruction, were rarely reported outcomes. This is making it difficult to confirm or dismiss ASPEN’s cautious approach.

Generally, classifying fiber only by its solubility is increasingly recognized as outdated, and recent expert opinion papers highlight the importance of considering additional physicochemical characteristics such as viscosity and fermentability [9, 10]. A nuanced understanding of fiber's properties and biological mechanisms could further inform the design of future clinical trials, increasing the possibility of detecting a true clinically significant benefit and potential adverse effects.

Most importantly, our GRADE assessments implicate that the true effect of fiber-supplemented EN is likely to be substantially different from the estimated effect. The TSA results suggest that the findings of our meta-analyses may be at risk of type-1 errors, and more robust studies are needed to validate whether a real difference exists. The high risk of bias among the included studies underscores the potential for overestimation of benefits. Overall, given the very low-certainty of evidence, no strong recommendations can be made regarding the routine use of fiber-supplemented EN in critically ill patients. Although diarrhea is a common and relevant symptom in critically ill patients and fiber-supplemented EN is relatively inexpensive, its potential benefits should be approached with caution. Given the potential type-1 errors and overestimations of effect, the possibility that DF could even be harmful cannot be excluded. Therefore, high-quality RCTs are needed to accumulate sufficient evidence and substantiate the efficacy and safety of fiber-supplemented EN. Until such evidence is available, clinicians should consider individual patient circumstances when deciding on the use of DF supplementation.

The results of our TSA further suggest that future trials should not be powered for mortality unless the expected effect size is large (e.g. a RRR of 30%), a magnitude more commonly observed in pharmaceutical trials. Instead, future studies should be powered for diarrhea incidence due to its high prevalence among critically ill patients and because the required sample size is relatively more achievable compared to the other outcomes.

### Strengths and limitations

Our SRMA has numerous strengths. We conducted a meticulous systematic search and performed robust quality and GRADE assessments. In addition, the meta-analysis of accurately selected RCTs enhances the overall quality of evidence of our SRMA compared to previous SRMAs. Including non-RCTs in a meta-analysis can lead to reduced reliability due to higher susceptibility to biases, increased methodological variability, and higher heterogeneity [26]. Finally, our SRMA is the first to explore the effects of fiber-supplemented EN through TSA, allowing us to estimate the required sample size for future trials. Overall, our SRMA provides the highest quality of evidence available on fiber-supplemented EN in critically ill patients.

Our SRMA also faces important limitations. Firstly, the studies included were predominantly single-centered and all had small sample sizes. None of the studies had a low risk of bias and most had a high risk of bias, which can lead to overestimations of benefits and underestimations of harm. Secondly, despite our efforts to obtain missing data from the authors, not all studies could be aggregated in the statistical analyses due to the diverse ways outcomes were measured and reported. For most outcomes, fewer than ten studies provided data for the meta-analysis, resulting in low patient numbers. Thirdly, as we excluded trials that only reported on metabolic or nutritional outcomes, our analysis provides an incomplete view of the evidence regarding these outcomes. Finally, in most subgroup analyses, not all studies from the overall analyses could be categorized into a defined subgroup due to missing data on subgroup characteristics. We also observed an uneven distribution of studies and population sizes across most of the subgroup analyses. Generally, the validity of our subgroup analyses is very low due to the very low certainty of evidence characterizing the overall results, which limits the interpretability and significance of these findings. Also, the multiplicity of subgroup analyses was not corrected for, and the findings from these analyses should be considered hypothesis-generating rather than confirmatory. Nonetheless, our subgroup analyses suggest several areas for future trials on tailored enteral nutrition, including the optimal type, dosage, start timing, and treatment duration of DF, as well as the patient populations that benefit most from DF supplementation.

## Conclusion

This SRMA with TSA shows very low-certainty evidence suggesting that fiber-supplemented EN has clinical benefits, and future trials should explore diarrheal incidence as a primary outcome. Overall, given the very low-certainty of evidence, current findings should not be considered definitive to guide clinical practice. Large-scale, high-quality RCTs are required to accumulate sufficient evidence to justify recommendations for the routine use of fiber-supplemented EN in critically ill patients. Additionally, the optimal DF type, dosage, start timing, and treatment duration, as well as the critically ill patient population that benefits the most from fiber-supplemented EN should be explored.

## Supplementary Information

Supplementary Material 1.

## Data Availability

All generated data are presented within the manuscript or the additional file.

## Abbreviations

ASPEN: American Society for Parenteral and Enteral Nutrition
CCN: Critical care nutrition
D^2^: Diversity estimate
DARIS: Diversity adjusted required information size
DF: Dietary fiber
EN: Enteral nutrition
ESPEN: European Society for Clinical Nutrition and Metabolism
ICU: Intensive care unit
LOS: Length of stay
MD: Mean difference
MIREDIF: Minimally relevant difference
MV: Mechanical ventilation
PRISMA: Preferred Reporting Items for Systematic Reviews and Meta-Analyses
RCT: Randomized controlled trial
RIS: Required information size
ROB2: Cochrane Risk of Bias 2
RR: Risk ratio
RRR: Relative risk reduction
SCCM: Society of Critical Care Medicine
SD: Standard deviation
TSA: Trial sequential analysis
