# Supplementary material for: The efficacy of fiber-supplemented enteral nutrition in critically ill patients: a systematic review and meta-analysis of randomized controlled trials with trial sequential analysis
Source: Crit Care. 2024 Nov 7;28:359. doi: 10.1186/s13054-024-05128-2 (PMC11545523; doi:10.1186/s13054-024-05128-2)
Supplement: Supplementary file 1 — Supplementary Material 1. [file 13054_2024_5128_MOESM1_ESM.docx]

**Additional file**

**The efficacy of fiber-supplemented enteral nutrition in critically ill patients: a systematic review and meta-analysis of randomized controlled trials with trial sequential analysis**

Jana Larissa Koch^1, 2^, Charles Chin Han Lew, PhD^3, 4^, PD Dr. med. Felix Kork^2^, Prof. Dr. med. Alexander Koch^5^, Prof. Dr. med. Christian Stoppe^6, 7^, Prof. Daren K. Heyland^8^, Dr. troph. Ellen Dresen^6^, Zheng-Yii Lee, PhD, MSc^7, 9, a^, Dr. med. Aileen Hill^2, 10, a, b^

^1^ Medical Faculty RWTH Aachen University, Aachen, Germany

^2^ Department of Anaesthesiology, University Hospital RWTH Aachen, Aachen, Germany

^3^ Department of Dietetics & Nutrition, Ng Teng Fong General Hospital, Singapore

^4^ Faculty of Health and Social Sciences, Singapore Institute of Technology, Singapore, Singapore

^5^ Department of Gastroenterology, Metabolic Diseases and Internal Intensive Care Medicine, University Hospital RWTH Aachen, Aachen, Germany

^6^ University Hospital Würzburg, Department of Anaesthesiology, Intensive Care, Emergency and Pain Medicine, Würzburg, Germany

^7^ Department of Cardiac Anaesthesiology and Intensive Care Medicine, Charité Berlin, Berlin, Germany

^8^ Department of Critical Care Medicine, Queen's University, Kingston, Ontario, Canada

^9^ Department of Anaesthesiology, University of Malaya, Kuala Lumpur, Malaysia

^10^ Department of Intensive Care Medicine, University Hospital RWTH Aachen, Aachen, Germany

^a^ Joint last author

^b^ Corresponding author:

Dr. med. Aileen Hill

University Hospital RWTH Aachen

Table of contents

[PART 1: Methods (additional description) 1](#_Toc177401243)

[1. Search strategies 1](#_Toc177401244)

[2. Critical Care Nutrition (CCN) methodological quality scoring system 11](#_Toc177401245)

[3. Trial Sequential Analysis 12](#_Toc177401246)

[3. PRISMA 2020 Checklist 13](#_Toc177401247)

[PART 2: Details of the characteristics, intervention, outcomes, and quality scoring of the included studies, and the list of excluded studies 16](#_Toc177401248)

[Table S1: Characteristics of included studies and patients 17](#_Toc177401249)

[Table S2: Funding source 20](#_Toc177401250)

[Table S3: Feeding information 21](#_Toc177401251)

[Table S4: Clinical outcomes 25](#_Toc177401252)

[Table S5: Metabolic and nutritional outcomes 29](#_Toc177401253)

[Table S6: Diarrheal outcome 31](#_Toc177401254)

[Table S7: Adverse events and serious adverse events 32](#_Toc177401255)

[Table S8: Clinical Trial Registry of ongoing or unpublished studies 32](#_Toc177401256)

[Table S9: List of excluded studies after full-text review with reasons 33](#_Toc177401257)

[Table S10: Canadian Critical Care Nutrition Methodological Scoring 37](#_Toc177401258)

[Figure S1: ROB2 traffic light and summary plots 38](#_Toc177401259)

[1a) Overall mortality 38](#_Toc177401260)

[1b) Diarrhea 39](#_Toc177401261)

[1c) Other GI complications 40](#_Toc177401262)

[1d) ICU LOS 40](#_Toc177401263)

[1e) Hospital LOS 41](#_Toc177401264)

[1f) Infectious complications 41](#_Toc177401265)

[1g) Duration of mechanical ventilation 42](#_Toc177401266)

[1h) Time to reach target energy needs 42](#_Toc177401267)

[PART 3: Results of the meta-analyses and subgroup analyses 43](#_Toc177401268)

[PART 3A: Subgroup analyses 44](#_Toc177401269)

[Table S11: Summary of the results of subgroup analyses 44](#_Toc177401270)

[Table S12: Calculation of daily fiber doses 46](#_Toc177401271)

[Figure S2: Overall mortality (subgroup analyses) 47](#_Toc177401272)

[2a) Publication date 47](#_Toc177401273)

[2b) Fermentability 47](#_Toc177401274)

[2c) Viscosity 48](#_Toc177401275)

[2d) Solubility 48](#_Toc177401276)

[2e) Daily fiber dose 49](#_Toc177401277)

[2f) Age 49](#_Toc177401278)

[2g) Disease severity 50](#_Toc177401279)

[2h) ICU admission type 50](#_Toc177401280)

[2i) Intervention start 51](#_Toc177401281)

[2j) Minimum duration of intervention 51](#_Toc177401282)

[2k) Co-intervention with immunonutrition 52](#_Toc177401283)

[2l) Funding source 52](#_Toc177401284)

[2m) Type of control group 53](#_Toc177401285)

[Figure S3: Diarrhea incidence (subgroup analyses) 54](#_Toc177401286)

[3a) Publication date 54](#_Toc177401287)

[3b) Fermentability 54](#_Toc177401288)

[3c) Viscosity 55](#_Toc177401289)

[3d) Solubility 55](#_Toc177401290)

[3e) Daily fiber dose 56](#_Toc177401291)

[3f) Age 56](#_Toc177401292)

[3g) Disease severity 57](#_Toc177401293)

[3h) ICU admission type 57](#_Toc177401294)

[3i) Intervention start 58](#_Toc177401295)

[3j) Minimum duration of intervention 58](#_Toc177401296)

[3k) Co-intervention with immunonutrition 59](#_Toc177401297)

[3l) Funding source 59](#_Toc177401298)

[3m) Type of control group 60](#_Toc177401299)

[PART 3B: Overall meta-analyses 61](#_Toc177401300)

[Figure S4: Diarrhea score 61](#_Toc177401301)

[Figure S5: Other GI complications 61](#_Toc177401302)

[5a) Patients with at least one GI complication 61](#_Toc177401303)

[5b) Abdominal distension 61](#_Toc177401304)

[5c) Vomiting 61](#_Toc177401305)

[5d) Regurgitation 62](#_Toc177401306)

[5e) GI bleeding 62](#_Toc177401307)

[5f) Constipation 62](#_Toc177401308)

[Figure S6: Infectious complications 62](#_Toc177401309)

[6a) Patients with at least 1 infectious complication 62](#_Toc177401310)

[6b) Pneumonia 62](#_Toc177401311)

[6c) Urinary tract infection 63](#_Toc177401312)

[6d) Intra-abdominal infection 63](#_Toc177401313)

[6e) Sepsis 63](#_Toc177401314)

[6f) Vascular infection 63](#_Toc177401315)

[6g) Wound infection 63](#_Toc177401316)

[6h) Bacteremia 63](#_Toc177401317)

[Figure S7: Duration of mechanical ventilation 64](#_Toc177401318)

[Figure S8: Time to reach target energy needs 64](#_Toc177401319)

[PART 3C: Funnel plots 65](#_Toc177401320)

[Figure S9: Mortality 65](#_Toc177401321)

[Figure S10: Diarrhea 65](#_Toc177401322)

[10a) Diarrhea incidence 65](#_Toc177401323)

[10b) Diarrhea score 66](#_Toc177401324)

[Figure S11: Other GI complications 66](#_Toc177401325)

[11a) Patients with at least 1 GI complication 66](#_Toc177401326)

[11b) Abdominal distension 67](#_Toc177401327)

[11c) Vomiting 67](#_Toc177401328)

[11d) Constipation 68](#_Toc177401329)

[11e) GI bleeding 68](#_Toc177401330)

[11f) Regurgitation 69](#_Toc177401331)

[Figure S12: ICU length of stay 69](#_Toc177401332)

[Figure S13: Hospital length of stay 70](#_Toc177401333)

[Figure S14: Infectious complications 70](#_Toc177401334)

[14a) Patients with at least 1 infectious complication 70](#_Toc177401335)

[14b) Pneumonia 71](#_Toc177401336)

[14c) Urinary tract infection 71](#_Toc177401337)

[14d) Intra-abdominal infection 72](#_Toc177401338)

[14e) Sepsis 72](#_Toc177401339)

[14f) Vascular infection 73](#_Toc177401340)

[14g) Wound infection 73](#_Toc177401341)

[14h) Bacteremia 74](#_Toc177401342)

[Figure S15: Duration of mechanical ventilation 74](#_Toc177401343)

[Figure S16: Time to reach target energy needs 75](#_Toc177401344)

[PART 4: Trial Sequential Analysis 76](#_Toc177401345)

[Fig. S17: Diarrhea incidence 76](#_Toc177401346)

[a) RRR = 25% 76](#_Toc177401347)

[b) sensitivity analysis – RRR = 15% 77](#_Toc177401348)

[c) sensitivity analysis – RRR = 35% 77](#_Toc177401349)

[Fig. S18: ICU LOS with sensitivity analyses 78](#_Toc177401350)

[a) MIREDIF = 1 day 78](#_Toc177401351)

[b) sensitivity analysis – MIREDIF = 2 days 78](#_Toc177401352)

[c) sensitivity analysis – MIREDIF = 3 days 79](#_Toc177401353)

[Fig. S19: Hospital LOS with sensitivity analyses 79](#_Toc177401354)

[a) MIREDIF = 1 day 79](#_Toc177401355)

[b) sensitivity analysis – MIREDIF = 2 days 80](#_Toc177401356)

[c) sensitivity analysis – MIREDIF = 3 days 80](#_Toc177401357)

[References 81](#_Toc177401358)

# PART 1: Methods (additional description)

### **1.** Search strategies

1. **Embase Classic+Embase <1947 to 2024 January 09>**

| **#** | **Query** | **Results from 11 Jan 2024** |
| --- | --- | --- |
| 1 | Critical illness/ | 36,387 |
| 2 | Intensive care/ | 151,692 |
| 3 | Artificial ventilation/ | 194,908 |
| 4 | Invasive ventilation/ | 9,292 |
| 5 | Invasive positive pressure ventilation/ | 99 |
| 6 | Intensive care medicine/ | 1,643 |
| 7 | Critically ill patient/ | 68,222 |
| 8 | Intensive care unit/ | 236,159 |
| 9 | Medical intensive care unit/ | 4,345 |
| 10 | Surgical Intensive care unit/ | 2,935 |
| 11 | Endotracheal intubation/ | 67,086 |
| 12 | Multiple organ failure/ | 51,168 |
| 13 | Sepsis/ | 211,264 |
| 14 | Bacteremia/ | 54,074 |
| 15 | Anaerobic bacteremia/ | 21 |
| 16 | Catheter-related bacteremia/ | 106 |
| 17 | Gram negative sepsis/ | 4,397 |
| 18 | Gram positive sepsis/ | 69 |
| 19 | Polymicrobial bacteremia/ | 28 |
| 20 | Fungemia/ | 4,411 |
| 21 | Candidemia/ | 5,518 |
| 22 | Cryptococcemia/ | 22 |
| 23 | Septic complication/ | 41 |
| 24 | Sepsis-associated encephalopathy/ | 669 |
| 25 | Sepsis-associated coagulopathy/ | 253 |
| 26 | Sepsis-induced acute lung injury/ | 86 |
| 27 | Sepsis-induced myocardial injury/ | 88 |
| 28 | Septicemia/ | 24,466 |
| 29 | Severe sepsis/ | 213 |
| 30 | Urosepsis/ | 4,404 |
| 31 | Hemorrhagic septicemia/ | 401 |
| 32 | Endotoxemia/ | 12,461 |
| 33 | shock/ | 43,744 |
| 34 | Septic shock/ | 70,100 |
| 35 | Toxic shock syndrome/ | 4,436 |
| 36 | Endotoxic shock/ | 122 |
| 37 | staphylococcal toxic shock syndrome/ | 43 |
| 38 | streptococcal toxic shock syndrome/ | 179 |
| 39 | capillary leak syndrome/ | 3,834 |
| 40 | cardiogenic shock/ | 39,631 |
| 41 | dengue shock syndrome/ | 559 |
| 42 | hemorrhagic shock/ | 21,780 |
| 43 | hypovolemic shock/ | 6,420 |
| 44 | ischemic shock/ | 12 |
| 45 | traumatic shock/ | 4,351 |
| 46 | vasodilatory shock/ | 526 |
| 47 | Systemic inflammatory response syndrome/ | 16,617 |
| 48 | Hyperinflammatory syndrome/ | 377 |
| 49 | Cytokine storm/ | 8,743 |
| 50 | Cytokine release syndrome/ | 9,005 |
| 51 | Adult respiratory distress syndrome/ | 57,372 |
| 52 | Acute lung injury/ | 18,588 |
| 53 | Transfusion related acute lung injury/ | 2,661 |
| 54 | Severe acute respiratory syndrome/ | 11,639 |
| 55 | Heart infarction/ | 343,148 |
| 56 | Heart arrest/ | 95,583 |
| 57 | cardiopulmonary arrest/ | 6,989 |
| 58 | out of hospital cardiac arrest/ | 15,331 |
| 59 | Extracorporeal oxygenation/ | 39,557 |
| 60 | arterio-venous ECMO/ | 126 |
| 61 | veno-arterial ECMO/ | 4,869 |
| 62 | veno-venous ECMO/ | 3,419 |
| 63 | Multiple trauma/ | 17,967 |
| 64 | brain injury/ | 104,039 |
| 65 | acquired brain injury/ | 2,830 |
| 66 | brain concussion/ | 8,491 |
| 67 | brain contusion/ | 4,704 |
| 68 | brain damage/ | 48,276 |
| 69 | brain stem injury/ | 2,959 |
| 70 | cerebellum injury/ | 2,701 |
| 71 | diffuse brain injury/ | 208 |
| 72 | traumatic brain injury/ | 67,978 |
| 73 | Acute liver failure/ | 11,894 |
| 74 | Acute on chronic liver failure/ | 5,691 |
| 75 | Fulminant hepatic failure/ | 2,536 |
| 76 | Acute pancreatitis/ | 40,439 |
| 77 | Acute hemorrhagic pancreatitis/ | 6,095 |
| 78 | Hemorrhagic pancreatitis/ | 618 |
| 79 | Acute kidney failure/ | 132,446 |
| 80 | Continuous renal replacement therapy/ | 9,018 |
| 81 | continuous hemodiafiltration/ | 2,761 |
| 82 | continuous hemodiafiltration/ | 2,761 |
| 83 | continuous hemodialysis/ | 1,713 |
| 84 | continuous hemofiltration/ | 3,108 |
| 85 | modified ultrafiltration/ | 280 |
| 86 | slow continuous ultrafiltration/ | 120 |
| 87 | intermittent renal replacement therapy/ | 120 |
| 88 | Burn/ | 73,321 |
| 89 | Burn shock/ | 702 |
| 90 | Electric burn/ | 2,876 |
| 91 | Burn unit/ | 3,287 |
| 92 | critical care.mp. | 76,744 |
| 93 | intensive care.mp. | 499,435 |
| 94 | critical illness.mp. | 45,368 |
| 95 | critically ill.mp. | 108,501 |
| 96 | ICU.ti,ab,kw. | 173,590 |
| 97 | Mechanical*2 ventilat*3.ti,ab,kw. | 116,741 |
| 98 | intubat*3.ti,kw. | 26,472 |
| 99 | Sepsis.mp. | 285,388 |
| 100 | Septicemia.mp. | 35,967 |
| 101 | Septic shock.mp. | 78,867 |
| 102 | Shock.ti,ab,kw. | 298,762 |
| 103 | Multiple organ failure.ti,ab,kw. | 12,493 |
| 104 | Multiple organ dysfunction syndrome.ti,ab,kw. | 3,852 |
| 105 | MODS.ti,ab,kw. | 3,908 |
| 106 | Systemic inflammatory response syndrome.ti,ab,kw. | 9,414 |
| 107 | Acute respiratory distress syndrome.mp. | 33,813 |
| 108 | ARDS.ti,ab,kw. | 33,137 |
| 109 | Acute lung injury.ti,ab,kw. | 25,550 |
| 110 | Myocardial infarction.ti,ab,kw. | 336,929 |
| 111 | Cardiac arrest.ti,ab,kw. | 75,336 |
| 112 | Extracorporeal Membrane Oxygenation.ti,ab,kw. | 26,316 |
| 113 | ECMO.ti,ab,kw. | 27,600 |
| 114 | Multiple trauma.ti,ab,kw. | 4,598 |
| 115 | Polytrauma.ti,ab,kw. | 6,472 |
| 116 | Multitrauma.ti,ab,kw. | 431 |
| 117 | Brain injur*3.ti,ab,kw. | 123,488 |
| 118 | Traumatic brain injury.ti,ab,kw. | 70,031 |
| 119 | TBI.ti,ab,kw. | 53,313 |
| 120 | Acute Liver failure.ti,ab,kw. | 14,286 |
| 121 | Acute pancreatitis.ti,ab,kw. | 43,792 |
| 122 | Acute kidney injury.ti,ab,kw. | 67,785 |
| 123 | AKI.ti,ab,kw. | 42,930 |
| 124 | Continuous renal replacement therapy.ti,ab,kw. | 6,656 |
| 125 | CRRT.ti,ab,kw. | 5,614 |
| 126 | Sustained low efficiency dialysis.ti,ab,kw. | 335 |
| 127 | SLED.ti,ab,kw. | 1,624 |
| 128 | Intermittent h?emodialysis.ti,ab,kw. | 2,638 |
| 129 | Burn unit.ti,ab,kw. | 2,104 |
| 130 | Burn patient.ti,ab,kw. | 1,725 |
| 131 | dietary fiber/ | 27,476 |
| 132 | prebiotic agent/ | 13,199 |
| 133 | resistant starch/ | 653 |
| 134 | arabinoxylan/ | 1,508 |
| 135 | beta glucan/ | 7,356 |
| 136 | cellulose/ | 46,566 |
| 137 | dextrin/ | 2,462 |
| 138 | guar gum/ | 3,640 |
| 139 | Hemicellulose/ | 7,849 |
| 140 | Inulin/ | 13,163 |
| 141 | Pectin/ | 12,511 |
| 142 | galactomannan/ | 4,815 |
| 143 | fructose oligosaccharide/ | 3,036 |
| 144 | galactose oligosaccharide/ | 1,399 |
| 145 | Lignin/ | 19,805 |
| 146 | methylcellulose/ | 8,896 |
| 147 | alginic acid/ | 31,199 |
| 148 | ispagula/ | 3,354 |
| 149 | Gum Arabic/ | 3,036 |
| 150 | Fiber.mp. | 499,870 |
| 151 | Fibre.mp. | 61,625 |
| 152 | Prebiotic.ti,ab,kw. | 10,404 |
| 153 | Resistant starch.ti,ab,kw. | 3,009 |
| 154 | arabinoxylan.ti,ab,kw. | 1,563 |
| 155 | Beta glucan.ti,ab,kw. | 6,523 |
| 156 | cellulose.ti,ab,kw. | 86,776 |
| 157 | dextrin.ti,ab,kw. | 2,386 |
| 158 | Guar gum.ti,ab,kw. | 2,883 |
| 159 | hemicellulose.ti,ab,kw. | 6,363 |
| 160 | inulin.ti,ab,kw. | 13,358 |
| 161 | pectin.ti,ab,kw. | 12,317 |
| 162 | galactomannan.ti,ab,kw. | 4,210 |
| 163 | fructose oligosaccharide.ti,ab,kw. | 10 |
| 164 | Fructooligosaccharide.ti,ab,kw. | 625 |
| 165 | FOS.ti,ab,kw. | 39,750 |
| 166 | galactose oligosaccharide.ti,ab,kw. | 2 |
| 167 | galactooligosaccharide.ti,ab,kw. | 261 |
| 168 | GOS.ti,ab,kw. | 6,939 |
| 169 | lignin.ti,ab,kw. | 20,891 |
| 170 | methylcellulose.ti,ab,kw. | 9,093 |
| 171 | Alginic acid.ti,ab,kw. | 890 |
| 172 | alginate.ti,ab,kw. | 31,140 |
| 173 | ispagula.ti,ab,kw. | 8 |
| 174 | ispaghula.ti,ab,kw. | 227 |
| 175 | Psyllium.ti,ab,kw. | 1,312 |
| 176 | Acacia gum.ti,ab,kw. | 242 |
| 177 | Gum Arabic.ti,ab,kw. | 1,808 |
| 178 | Artificial feeding/ | 3,277 |
| 179 | Enteric feeding/ | 41,731 |
| 180 | Nose feeding/ | 4,627 |
| 181 | Digestive tract intubation/ | 4,499 |
| 182 | Duodenum intubation/ | 487 |
| 183 | Stomach intubation/ | 1,805 |
| 184 | Dietary supplement/ | 24,558 |
| 185 | Nutrition supplement/ | 6,620 |
| 186 | Enteral nutrition.mp. | 19,790 |
| 187 | Enteral feeding.mp. | 9,973 |
| 188 | Enteric feeding.mp. | 41,802 |
| 189 | Enteral formula.mp. | 728 |
| 190 | Enteral product.mp. | 34 |
| 191 | (nutrition*2 adj3 support).mp. | 35,410 |
| 192 | EN.ti,ab,kw. | 114,739 |
| 193 | EN modular product.ti,ab,kw. | 0 |
| 194 | Dietary supplement.ti,ab,kw. | 9,922 |
| 195 | Nutrition supplement.ti,ab,kw. | 383 |
| 196 | or/1-130 | 2,433,924 |
| 197 | or/131-177 | 798,780 |
| 198 | or/178-195 | 233,695 |
| 199 | 196 and 197 and 198 | 687 |
| 200 | Clinical trial/ or Randomized controlled trial/ or Randomization/ or Single blind procedure/ or Double blind procedure/ or Crossover procedure/ or Placebo/ or Randomi?ed controlled trial$.tw. or Rct.tw. or Random allocation.tw. or Randomly allocated.tw. or Allocated randomly.tw. or (allocated adj2 random).tw. or Single blind$.tw. or Double blind$.tw. or ((treble or triple) adj blind$).tw. or Placebo$.tw. or Prospective study/ | 2,855,272 |
| 201 | Case study/ or Case report.tw. or Abstract report/ or letter/ | 1,987,705 |
| 202 | 200 not 201 | 2,788,218 |
| 203 | 199 and 202 | 201 |
| 204 | (exp adolescence/ or exp adolescent/ or exp child/ or exp childhood disease/ or exp infant disease/ or (adolescen* or babies or baby or boy? or boyfriend or boyhood or girlfriend or girlhood or child* or girl? or infan* or juvenil* or juvenile* or kid? or minors or minors* or neonat* or neonat* or neo-nata* or newborn* or new-born* or paediatric* or peadiatric* or pediatric* or perinat* or preschool* or puber* or pubescen* or school or school child* or school* or schoolchild* or schoolchild* or teen* or toddler? or underage? or under-age? or youth*).ti,ab,kw.) not exp adult/ | 4,601,691 |
| 205 | 203 not 204 | 161 |
| 206 | (exp animal/ or exp invertebrate/ or nonhuman/ or animal experiment/ or animal tissue/ or animal model/ or exp plant/ or fungus/) not (exp human/ or human tissue/) | 8,589,070 |
| 207 | 205 not 206 | 158 |

1. **Ovid MEDLINE(R) and Epub Ahead of Print, In-Process, In-Data-Review & Other Non-Indexed Citations and Daily <1946 to January 09, 2024>**

| **#** | **Query** | **Results from 11 Jan 2024** |
| --- | --- | --- |
| 1 | Critical Care/ | 61,453 |
| 2 | Critical Illness/ | 39,846 |
| 3 | Intensive Care Units/ | 71,957 |
| 4 | intubation, intratracheal/ | 40,081 |
| 5 | respiration, artificial/ | 57,535 |
| 6 | sepsis/ | 72,677 |
| 7 | Bacteremia/ | 28,273 |
| 8 | Endotoxemia/ | 4,811 |
| 9 | Hemorrhagic Septicemia/ | 241 |
| 10 | Fungemia/ | 3,176 |
| 11 | Candidemia/ | 1,595 |
| 12 | shock/ | 18,583 |
| 13 | multiple organ failure/ | 12,155 |
| 14 | shock, cardiogenic/ | 11,018 |
| 15 | shock, hemorrhagic/ | 12,389 |
| 16 | shock, surgical/ | 1,061 |
| 17 | shock, traumatic/ | 4,385 |
| 18 | systemic inflammatory response syndrome/ | 7,597 |
| 19 | cytokine release syndrome/ | 2,284 |
| 20 | shock, septic/ | 25,294 |
| 21 | Respiratory Distress Syndrome/ | 25,264 |
| 22 | Acute lung injury/ | 8,771 |
| 23 | Transfusion-related acute lung injury/ | 141 |
| 24 | Myocardial infarction/ | 181,165 |
| 25 | Heart Arrest/ | 32,851 |
| 26 | Out-of-Hospital Cardiac Arrest/ | 7,373 |
| 27 | Extracorporeal Membrane Oxygenation/ | 15,766 |
| 28 | Multiple Trauma/ | 13,535 |
| 29 | Brain Injuries/ | 56,872 |
| 30 | Brain Hemorrhage, Traumatic/ | 240 |
| 31 | Brain Injuries, Diffuse/ | 62 |
| 32 | Brain Injuries, Traumatic/ | 13,680 |
| 33 | Liver Failure, Acute/ | 6,226 |
| 34 | Acute-On-Chronic Liver Failure/ | 1,437 |
| 35 | Pancreatitis, Acute Hemorrhagic/ | 5 |
| 36 | Pancreatitis, Acute Necrotizing/ | 3,872 |
| 37 | Acute Kidney Injury/ | 55,763 |
| 38 | Continuous Renal Replacement Therapy/ | 872 |
| 39 | Hemofiltration/ | 4,816 |
| 40 | Hemodiafiltration/ | 2,642 |
| 41 | Hybrid renal replacement therapy/ | 33 |
| 42 | Intermittent renal replacement therapy/ | 33 |
| 43 | Burns/ | 49,129 |
| 44 | Burns, Chemical/ | 6,767 |
| 45 | Burns, Electric/ | 2,492 |
| 46 | Burn units/ | 2,994 |
| 47 | critical care.mp. | 91,150 |
| 48 | intensive care.mp. | 236,527 |
| 49 | critical illness.mp. | 46,916 |
| 50 | critically ill.mp. | 62,163 |
| 51 | ICU.ti,ab,kw. | 87,343 |
| 52 | Mechanical*2 ventilat*3.ti,ab,kw. | 71,312 |
| 53 | intubat*3.ti,kw. | 20,153 |
| 54 | Sepsis.mp. | 154,159 |
| 55 | Septicemia.mp. | 16,126 |
| 56 | Septic shock.mp. | 28,400 |
| 57 | Shock.ti,ab,kw. | 208,885 |
| 58 | Multiple organ failure.ti,ab,kw. | 8,481 |
| 59 | Multiple organ dysfunction syndrome.ti,ab,kw. | 2,784 |
| 60 | MODS.ti,ab,kw. | 2,473 |
| 61 | Systemic inflammatory response syndrome.ti,ab,kw. | 6,133 |
| 62 | Acute respiratory distress syndrome.mp. | 23,764 |
| 63 | ARDS.ti,ab,kw. | 19,926 |
| 64 | Acute lung injury.ti,ab,kw. | 17,503 |
| 65 | Myocardial infarction.ti,ab,kw. | 213,853 |
| 66 | Cardiac arrest.ti,ab,kw. | 44,349 |
| 67 | Extracorporeal Membrane Oxygenation.ti,ab,kw. | 17,678 |
| 68 | ECMO.ti,ab,kw. | 13,126 |
| 69 | Multiple trauma.ti,ab,kw. | 3,475 |
| 70 | Polytrauma.ti,ab,kw. | 4,564 |
| 71 | Multitrauma.ti,ab,kw. | 323 |
| 72 | Brain injur*3.ti,ab,kw. | 85,960 |
| 73 | Traumatic brain injury.ti,ab,kw. | 48,183 |
| 74 | TBI.ti,ab,kw. | 32,291 |
| 75 | Acute Liver failure.ti,ab,kw. | 8,245 |
| 76 | Acute pancreatitis.ti,ab,kw. | 28,166 |
| 77 | Acute kidney injury.ti,ab,kw. | 39,623 |
| 78 | Acute kidney injury.ti,ab,kw. | 39,623 |
| 79 | AKI.ti,ab,kw. | 22,295 |
| 80 | Continuous renal replacement therapy.ti,ab,kw. | 3,777 |
| 81 | CRRT.ti,ab,kw. | 2,586 |
| 82 | Sustained low efficiency dialysis.ti,ab,kw. | 178 |
| 83 | SLED.ti,ab,kw. | 1,294 |
| 84 | Intermittent h?emodialysis.ti,ab,kw. | 1,549 |
| 85 | Burn unit.ti,ab,kw. | 1,399 |
| 86 | Burn patient.ti,ab,kw. | 1,153 |
| 87 | dietary fiber/ | 19,863 |
| 88 | prebiotics/ | 4,378 |
| 89 | resistant starch/ | 309 |
| 90 | Alginates/ | 15,109 |
| 91 | Alginic acid/ | 96 |
| 92 | beta glucan/ | 5,134 |
| 93 | Cellulose/ | 34,569 |
| 94 | Methylcellulose/ | 4,837 |
| 95 | Lignin/ | 14,042 |
| 96 | Dextrin/ | 1,114 |
| 97 | Inulin/ | 7,510 |
| 98 | Pectin/ | 7,172 |
| 99 | Mannans/ | 6,538 |
| 100 | Oligosaccharides, Branched-Chain/ | 134 |
| 101 | Xylans/ | 3,742 |
| 102 | Psyllium/ | 744 |
| 103 | Gum Arabic/ | 998 |
| 104 | Fiber.mp. | 218,188 |
| 105 | Fibre.mp. | 45,548 |
| 106 | Prebiotic.ti,ab,kw. | 9,306 |
| 107 | Resistant starch.ti,ab,kw. | 2,718 |
| 108 | Alginic acid.ti,ab,kw. | 691 |
| 109 | alginate.ti,ab,kw. | 25,714 |
| 110 | Beta glucan.ti,ab,kw. | 5,795 |
| 111 | cellulose.ti,ab,kw. | 75,359 |
| 112 | dextrin.ti,ab,kw. | 1,654 |
| 113 | Guar gum.ti,ab,kw. | 2,120 |
| 114 | hemicellulose.ti,ab,kw. | 6,504 |
| 115 | inulin.ti,ab,kw. | 9,793 |
| 116 | pectin.ti,ab,kw. | 11,372 |
| 117 | galactomannan.ti,ab,kw. | 2,728 |
| 118 | lignin.ti,ab,kw. | 21,529 |
| 119 | methylcellulose.ti,ab,kw. | 6,223 |
| 120 | fructose oligosaccharide.ti,ab,kw. | 5 |
| 121 | Fructooligosaccharide.ti,ab,kw. | 512 |
| 122 | FOS.ti,ab,kw. | 33,130 |
| 123 | galactose oligosaccharide.ti,ab,kw. | 4 |
| 124 | galactooligosaccharide.ti,ab,kw. | 207 |
| 125 | GOS.ti,ab,kw. | 4,668 |
| 126 | arabinoxylan.ti,ab,kw. | 1,513 |
| 127 | ispagula.ti,ab,kw. | 5 |
| 128 | ispaghula.ti,ab,kw. | 152 |
| 129 | Psyllium.ti,ab,kw. | 913 |
| 130 | Acacia gum.ti,ab,kw. | 174 |
| 131 | Gum Arabic.ti,ab,kw. | 1,616 |
| 132 | Nutritional support/ | 7,222 |
| 133 | Enteral nutrition/ | 22,146 |
| 134 | Intubation, Gastrointestinal/ | 10,297 |
| 135 | Food, formulated/ | 6,225 |
| 136 | Dietary Supplements/ | 76,010 |
| 137 | Enteral nutrition.mp. | 26,633 |
| 138 | Enteral feeding.mp. | 6,373 |
| 139 | Enteric feeding.mp. | 90 |
| 140 | Enteral formula.mp. | 506 |
| 141 | Enteral product.mp. | 22 |
| 142 | (nutrition*2 adj3 support).mp. | 19,595 |
| 143 | EN.ti,ab,kw. | 113,624 |
| 144 | EN modular product.ti,ab,kw. | 0 |
| 145 | Dietary supplement.ti,ab,kw. | 7,584 |
| 146 | Nutrition supplement.ti,ab,kw. | 235 |
| 147 | or/1-86 | 1,397,472 |
| 148 | or/87-131 | 475,490 |
| 149 | or/132-146 | 245,516 |
| 150 | 147 and 148 and 149 | 322 |
| 151 | Randomized controlled trials as Topic/ or Randomized controlled trial/ or Random allocation/ or Double blind method/ or Single blind method/ or Clinical trial/ or exp Clinical Trials as Topic/ | 1,336,810 |
| 152 | ((clinic$ adj trial$1) or ((singl$ or doubl$ or treb$ or tripl$) adj (blind$3 or mask$3))).tw. or Placebos/ or Placebo$.tw. or Randomly allocated.tw. or (allocated adj2 random).tw. | 805,409 |
| 153 | 151 or 152 | 1,712,825 |
| 154 | Case report.tw. or Letter/ or Historical article/ or Review of reported cases.pt. or Review, multicase.pt. | 2,003,756 |
| 155 | 153 not 154 | 1,673,140 |
| 156 | 150 and 155 | 96 |
| 157 | (exp adolescent/ or exp child/ or exp infant/ or (infant disease* or childhood disease*).ti,ab,kf. or (adolescen* or babies or baby or boy? or boyfriend or boyhood or girlfriend or girlhood or child* or girl? or infan* or juvenil* or kid? or minors or minors* or neonat* or neo-nat* or newborn* or new-born* or paediatric* or peadiatric* or pediatric* or perinat* or preschool* or puber* or pubescen* or school* or teen* or toddler? or underage? or under-age? or youth*).ti,ab,kf. or (pediatric* or paediatric* or infan* or child* or adolescen* or young).jn,jw. or (pediatric* or paediatric* or infan* or child* or adolescen* or young).in.) not exp adult/ | 3,696,273 |
| 158 | 156 not 157 | 71 |
| 159 | (exp animal/ or exp invertebrate/ or animal experiment/ or animal model/ or exp plant/ or exp fungus/) not exp human/ | 5,619,584 |
| 160 | 158 not 159 | 66 |

1. **EBM Reviews - Cochrane Central Register of Controlled Trials <December 2023>**

| **#** | **Query** | **Results from 11 Jan 2024** |
| --- | --- | --- |
| 1 | Critical Care/ | 2,269 |
| 2 | Critical Illness/ | 3,254 |
| 3 | Intensive Care Units/ | 3,302 |
| 4 | intubation, intratracheal/ | 4,459 |
| 5 | respiration, artificial/ | 4,297 |
| 6 | sepsis/ | 3,423 |
| 7 | Bacteremia/ | 962 |
| 8 | Endotoxemia/ | 335 |
| 9 | Hemorrhagic Septicemia/ | 0 |
| 10 | Fungemia/ | 78 |
| 11 | Candidemia/ | 49 |
| 12 | shock/ | 759 |
| 13 | multiple organ failure/ | 629 |
| 14 | shock, cardiogenic/ | 413 |
| 15 | shock, hemorrhagic/ | 158 |
| 16 | shock, surgical/ | 10 |
| 17 | shock, traumatic/ | 61 |
| 18 | systemic inflammatory response syndrome/ | 541 |
| 19 | cytokine release syndrome/ | 114 |
| 20 | shock, septic/ | 1,269 |
| 21 | Respiratory Distress Syndrome/ | 1,941 |
| 22 | Acute lung injury/ | 713 |
| 23 | Transfusion-related acute lung injury/ | 8 |
| 24 | Myocardial infarction/ | 13,057 |
| 25 | Heart Arrest/ | 1,581 |
| 26 | Out-of-Hospital Cardiac Arrest/ | 719 |
| 27 | Extracorporeal Membrane Oxygenation/ | 300 |
| 28 | Multiple Trauma/ | 292 |
| 29 | Brain Injuries/ | 2,301 |
| 30 | Brain Hemorrhage, Traumatic/ | 7 |
| 31 | Brain Injuries, Diffuse/ | 3 |
| 32 | Brain Injuries, Traumatic/ | 1,020 |
| 33 | Liver Failure, Acute/ | 103 |
| 34 | Acute-On-Chronic Liver Failure/ | 129 |
| 35 | Pancreatitis, Acute Hemorrhagic/ | 0 |
| 36 | Pancreatitis, Acute Necrotizing/ | 168 |
| 37 | Acute Kidney Injury/ | 2,006 |
| 38 | Continuous Renal Replacement Therapy/ | 95 |
| 39 | Hemofiltration/ | 441 |
| 40 | Hemodiafiltration/ | 356 |
| 41 | Hybrid renal replacement therapy/ | 0 |
| 42 | Intermittent renal replacement therapy/ | 0 |
| 43 | Burns/ | 1,775 |
| 44 | Burns, Chemical/ | 46 |
| 45 | Burns, Electric/ | 15 |
| 46 | Burn units/ | 49 |
| 47 | critical care.mp. | 5,044 |
| 48 | intensive care.mp. | 30,611 |
| 49 | critical illness.mp. | 4,572 |
| 50 | critically ill.mp. | 8,729 |
| 51 | ICU.ti,ab,kw. | 17,802 |
| 52 | Mechanical*2 ventilat*3.ti,ab,kw. | 15,121 |
| 53 | intubat*3.ti,kw. | 11,227 |
| 54 | Sepsis.mp. | 13,430 |
| 55 | Septicemia.mp. | 713 |
| 56 | Septic shock.mp. | 3,745 |
| 57 | Shock.ti,ab,kw. | 12,562 |
| 58 | Multiple organ failure.ti,ab,kw. | 1,077 |
| 59 | Multiple organ dysfunction syndrome.ti,ab,kw. | 278 |
| 60 | MODS.ti,ab,kw. | 333 |
| 61 | Systemic inflammatory response syndrome.ti,ab,kw. | 916 |
| 62 | Acute respiratory distress syndrome.mp. | 2,397 |
| 63 | ARDS.ti,ab,kw. | 2,676 |
| 64 | Acute lung injury.ti,ab,kw. | 1,058 |
| 65 | Myocardial infarction.ti,ab,kw. | 32,885 |
| 66 | Cardiac arrest.ti,ab,kw. | 4,456 |
| 67 | Extracorporeal Membrane Oxygenation.ti,ab,kw. | 735 |
| 68 | ECMO.ti,ab,kw. | 932 |
| 69 | Multiple trauma.ti,ab,kw. | 432 |
| 70 | Polytrauma.ti,ab,kw. | 191 |
| 71 | Multitrauma.ti,ab,kw. | 19 |
| 72 | Brain injur*3.ti,ab,kw. | 7,379 |
| 73 | Traumatic brain injury.ti,ab,kw. | 4,422 |
| 74 | TBI.ti,ab,kw. | 3,084 |
| 75 | Acute Liver failure.ti,ab,kw. | 254 |
| 76 | Acute pancreatitis.ti,ab,kw. | 2,183 |
| 77 | Acute kidney injury.ti,ab,kw. | 3,908 |
| 78 | Acute kidney injury.ti,ab,kw. | 3,908 |
| 79 | AKI.ti,ab,kw. | 2,338 |
| 80 | Continuous renal replacement therapy.ti,ab,kw. | 529 |
| 81 | CRRT.ti,ab,kw. | 480 |
| 82 | Sustained low efficiency dialysis.ti,ab,kw. | 35 |
| 83 | SLED.ti,ab,kw. | 124 |
| 84 | Intermittent h?emodialysis.ti,ab,kw. | 177 |
| 85 | Burn unit.ti,ab,kw. | 123 |
| 86 | Burn patient.ti,ab,kw. | 279 |
| 87 | dietary fiber/ | 2,130 |
| 88 | prebiotics/ | 409 |
| 89 | resistant starch/ | 32 |
| 90 | Alginates/ | 295 |
| 91 | Alginic acid/ | 40 |
| 92 | beta glucan/ | 194 |
| 93 | Cellulose/ | 481 |
| 94 | Methylcellulose/ | 141 |
| 95 | Lignin/ | 12 |
| 96 | Dextrin/ | 42 |
| 97 | Inulin/ | 290 |
| 98 | Pectin/ | 19 |
| 99 | Mannans/ | 286 |
| 100 | Oligosaccharides, Branched-Chain/ | 0 |
| 101 | Xylans/ | 43 |
| 102 | Psyllium/ | 210 |
| 103 | Gum Arabic/ | 35 |
| 104 | Fiber.mp. | 11,226 |
| 105 | Fibre.mp. | 3,228 |
| 106 | Prebiotic.ti,ab,kw. | 1,427 |
| 107 | Resistant starch.ti,ab,kw. | 472 |
| 108 | Alginic acid.ti,ab,kw. | 67 |
| 109 | alginate.ti,ab,kw. | 878 |
| 110 | Beta glucan.ti,ab,kw. | 488 |
| 111 | cellulose.ti,ab,kw. | 1,942 |
| 112 | dextrin.ti,ab,kw. | 292 |
| 113 | Guar gum.ti,ab,kw. | 282 |
| 114 | hemicellulose.ti,ab,kw. | 32 |
| 115 | inulin.ti,ab,kw. | 1,216 |
| 116 | pectin.ti,ab,kw. | 349 |
| 117 | galactomannan.ti,ab,kw. | 103 |
| 118 | lignin.ti,ab,kw. | 43 |
| 119 | methylcellulose.ti,ab,kw. | 310 |
| 120 | fructose oligosaccharide.ti,ab,kw. | 54 |
| 121 | Fructooligosaccharide.ti,ab,kw. | 131 |
| 122 | FOS.ti,ab,kw. | 489 |
| 123 | galactose oligosaccharide.ti,ab,kw. | 26 |
| 124 | galactooligosaccharide.ti,ab,kw. | 62 |
| 125 | GOS.ti,ab,kw. | 884 |
| 126 | arabinoxylan.ti,ab,kw. | 96 |
| 127 | ispagula.ti,ab,kw. | 18 |
| 128 | ispaghula.ti,ab,kw. | 72 |
| 129 | Psyllium.ti,ab,kw. | 395 |
| 130 | Acacia gum.ti,ab,kw. | 24 |
| 131 | Gum Arabic.ti,ab,kw. | 57 |
| 132 | Nutritional support/ | 494 |
| 133 | Enteral nutrition/ | 2,136 |
| 134 | Intubation, Gastrointestinal/ | 743 |
| 135 | Food, formulated/ | 815 |
| 136 | Dietary Supplements/ | 14,102 |
| 137 | Enteral nutrition.mp. | 5,080 |
| 138 | Enteral feeding.mp. | 1,690 |
| 139 | Enteric feeding.mp. | 1,852 |
| 140 | Enteral formula.mp. | 255 |
| 141 | Enteral product.mp. | 11 |
| 142 | (nutrition*2 adj3 support).mp. | 3,155 |
| 143 | EN.ti,ab,kw. | 8,445 |
| 144 | EN modular product.ti,ab,kw. | 0 |
| 145 | Dietary supplement.ti,ab,kw. | 3,777 |
| 146 | Nutrition supplement.ti,ab,kw. | 232 |
| 147 | or/1-86 | 133,355 |
| 148 | or/87-131 | 21,064 |
| 149 | or/132-146 | 34,115 |
| 150 | 147 and 148 and 149 | 139 |

### 2. Critical Care Nutrition (CCN) methodological quality scoring system

|  | **Score** | | | | | | |
| --- | --- | --- | --- | --- | --- | --- | --- |
|  | **0** | | **1** | | | **2** | |
| Randomization |  | | Not concealed or not sure |  | | Concealment of allocation* |  |
| Analysis | Other |  |  | | | Intention to treat |  |
| Blinding | Not blinded |  | Single blinded  *Check who was blinded:*  Health Care Professionals  Outcomes Assessors | |  | Double blinded |  |
| Patient selection | Selected patients or unable to tell |  | Consecutive eligible patients | |  |  | |
| Comparability of groups at baseline | No or not sure |  | Yes | |  |  | |
| Extent of follow-up | < 100% |  | 100% | |  |  | |
| Treatment protocol | Poorly described |  | Reproducibly described | |  |  | |
| Co-interventions** | Not described |  | Described but not equal or not sure | |  | Well described and all equal |  |
| Outcomes | Not described |  | Partially described | |  | Objectively defined |  |

* Concealment of allocation means the person enrolling the patients is unaware of the next treatment assignment (e.g. phone in randomization, computer generated).

** Extent to which antibiotics, ventilation, oxygen, transfusions, etc were applied equally across groups

### 3. Trial Sequential Analysis

Type I and type II errors can influence a SRMA, resulting in incorrect conclusions and inappropriate clinical practices. In conventional meta-analyses, the practice of maintaining a universal statistical threshold at p < 0.05 regardless of the total sample size and number of multiple testing can lead to false-positive results. To minimize type I error, a TSA imposes a high threshold for statistical significance (p-value much smaller than 0.05) in earlier trials and becomes less restrictive as the sample size increases with each additional trial. To achieve this, TSA boundaries are constructed. Crossing these boundaries indicates a high certainty of the presence of either a positive or negative effect. Futility boundaries are also constructed to control type II error. Crossing the futility boundaries indicates evidence of an absence of effect, suggesting that continuing to collect data might be futile in terms of finding a significant effect. Not crossing both the futility and TSA boundaries implies that the evidence is inconclusive and that more trials are needed to confirm the presence or absence of an effect [1].

We performed TSA with the following parameters: alpha 5%, beta 10%, and the DerSimonian-Laird random-effect model with a constant continuity correction of 0.5 per group for zero-event studies. Between-trial heterogeneity was adjusted by the diversity-estimate (D^2^). For overall mortality and diarrhea incidence, event rates in the control group were estimated from the pooled observed event rate in the current meta-analysis. For ICU and hospital LOS the variance was based on the pooled observed standard deviation in the current meta-analysis. We pre-specified the relative risk reduction (RRR) for overall mortality (10%) and diarrhea incidence (25%), and the minimally relevant difference (MIREDIF) for ICU and hospital LOS (both 1 day) on a clinically meaningful magnitude. Post-hoc, we performed sensitivity analyses for a RRR of 20% and 30% for mortality and 15% and 35% for diarrhea incidence. Additionally, we conducted sensitivity analyses for a MIREDIF of 2 and 3 days for ICU and hospital LOS.

### 3. PRISMA 2020 Checklist

| **Section and Topic** | **Item #** | **Checklist item** | **Location where item is reported** |
| --- | --- | --- | --- |
| **TITLE** | | |  |
| Title | 1 | Identify the report as a systematic review. | Page 1 |
| **ABSTRACT** | | |  |
| Abstract | 2 | See the PRISMA 2020 for Abstracts checklist. | Page 2 |
| **INTRODUCTION** | | |  |
| Rationale | 3 | Describe the rationale for the review in the context of existing knowledge. | Page 4-5 |
| Objectives | 4 | Provide an explicit statement of the objective(s) or question(s) the review addresses. | Page 5 |
| **METHODS** | | |  |
| Eligibility criteria | 5 | Specify the inclusion and exclusion criteria for the review and how studies were grouped for the syntheses. | Page 5-6 |
| Information sources | 6 | Specify all databases, registers, websites, organisations, reference lists and other sources searched or consulted to identify studies. Specify the date when each source was last searched or consulted. | Page 6 |
| Search strategy | 7 | Present the full search strategies for all databases, registers and websites, including any filters and limits used. | Suppl. file, Part 1 |
| Selection process | 8 | Specify the methods used to decide whether a study met the inclusion criteria of the review, including how many reviewers screened each record and each report retrieved, whether they worked independently, and if applicable, details of automation tools used in the process. | Page 6 |
| Data collection process | 9 | Specify the methods used to collect data from reports, including how many reviewers collected data from each report, whether they worked independently, any processes for obtaining or confirming data from study investigators, and if applicable, details of automation tools used in the process. | Page 6-7 |
| Data items | 10a | List and define all outcomes for which data were sought. Specify whether all results that were compatible with each outcome domain in each study were sought (e.g. for all measures, time points, analyses), and if not, the methods used to decide which results to collect. | Page 6 |
|  | 10b | List and define all other variables for which data were sought (e.g. participant and intervention characteristics, funding sources). Describe any assumptions made about any missing or unclear information. | Page 6-7 |
| Study risk of bias assessment | 11 | Specify the methods used to assess risk of bias in the included studies, including details of the tool(s) used, how many reviewers assessed each study and whether they worked independently, and if applicable, details of automation tools used in the process. | Page 7 |
| Effect measures | 12 | Specify for each outcome the effect measure(s) (e.g. risk ratio, mean difference) used in the synthesis or presentation of results. | Page 7 |
| Synthesis methods | 13a | Describe the processes used to decide which studies were eligible for each synthesis (e.g. tabulating the study intervention characteristics and comparing against the planned groups for each synthesis (item #5)). | Page 6 |
|  | 13b | Describe any methods required to prepare the data for presentation or synthesis, such as handling of missing summary statistics, or data conversions. | Page 6-7 |
|  | 13c | Describe any methods used to tabulate or visually display results of individual studies and syntheses. | Page 7 |
|  | 13d | Describe any methods used to synthesize results and provide a rationale for the choice(s). If meta-analysis was performed, describe the model(s), method(s) to identify the presence and extent of statistical heterogeneity, and software package(s) used. | Page 7-8 |
|  | 13e | Describe any methods used to explore possible causes of heterogeneity among study results (e.g. subgroup analysis, meta-regression). | Page 7-8 |
|  | 13f | Describe any sensitivity analyses conducted to assess robustness of the synthesized results. | N/A |
| Reporting bias assessment | 14 | Describe any methods used to assess risk of bias due to missing results in a synthesis (arising from reporting biases). | Page 7 |
| Certainty assessment | 15 | Describe any methods used to assess certainty (or confidence) in the body of evidence for an outcome. | Page 8 |
| **RESULTS** | | |  |
| Study selection | 16a | Describe the results of the search and selection process, from the number of records identified in the search to the number of studies included in the review, ideally using a flow diagram. | Page 9-10 |
|  | 16b | Cite studies that might appear to meet the inclusion criteria, but which were excluded, and explain why they were excluded. | Page 9; Suppl. file, Table S9 |
| Study characteristics | 17 | Cite each included study and present its characteristics. | Page 11; Suppl. file, Table S1 |
| Risk of bias in studies | 18 | Present assessments of risk of bias for each included study. | Page 10; Suppl. file, Table S9 and Fig. S1 |
| Results of individual studies | 19 | For all outcomes, present, for each study: (a) summary statistics for each group (where appropriate) and (b) an effect estimate and its precision (e.g. confidence/credible interval), ideally using structured tables or plots. | Fig. 2 – 5; Suppl. file, Fig. S2-S8, Table S11 |
| Results of syntheses | 20a | For each synthesis, briefly summarise the characteristics and risk of bias among contributing studies. | Fig. S1 |
|  | 20b | Present results of all statistical syntheses conducted. If meta-analysis was done, present for each the summary estimate and its precision (e.g. confidence/credible interval) and measures of statistical heterogeneity. If comparing groups, describe the direction of the effect. | Page 11-19; Fig. 2 – 5; Suppl. file, Fig. S2-S8, Table S11 |
|  | 20c | Present results of all investigations of possible causes of heterogeneity among study results. | Page 11-13 |
|  | 20d | Present results of all sensitivity analyses conducted to assess the robustness of the synthesized results. | 15-16 |
| Reporting biases | 21 | Present assessments of risk of bias due to missing results (arising from reporting biases) for each synthesis assessed. | Page 11-15; Fig. S9-S16 |
| Certainty of evidence | 22 | Present assessments of certainty (or confidence) in the body of evidence for each outcome assessed. | Page 18-19; Table 2 |
| **DISCUSSION** | | |  |
| Discussion | 23a | Provide a general interpretation of the results in the context of other evidence. | Page 19 |
|  | 23b | Discuss any limitations of the evidence included in the review. | Page 21 |
|  | 23c | Discuss any limitations of the review processes used. | Page 21 |
|  | 23d | Discuss implications of the results for practice, policy, and future research. | Page 20-21 |
| **OTHER INFORMATION** | | |  |
| Registration and protocol | 24a | Provide registration information for the review, including register name and registration number, or state that the review was not registered. | Page 5 |
|  | 24b | Indicate where the review protocol can be accessed, or state that a protocol was not prepared. | Page 5 |
|  | 24c | Describe and explain any amendments to information provided at registration or in the protocol. | N/A |
| Support | 25 | Describe sources of financial or non-financial support for the review, and the role of the funders or sponsors in the review. | Page 3 |
| Competing interests | 26 | Declare any competing interests of review authors. | Page 3 |
| Availability of data, code and other materials | 27 | Report which of the following are publicly available and where they can be found: template data collection forms; data extracted from included studies; data used for all analyses; analytic code; any other materials used in the review. | Page 3 |

# PART 2: Details of the characteristics, intervention, outcomes, and quality scoring of the included studies, and the list of excluded studies

### Table S1: Characteristics of included studies and patients

| **Reference No** | **Author, year (country)** | **Single- or Multicenter; N** | **n; Patient Population** | **Age** | | **Sex (Male/Female)** | | **Disease severity** | | **MV n (%)** | | **Admission category n (%)** | | **Weight, kg or BMI, kg/m²** | |
| --- | --- | --- | --- | --- | --- | --- | --- | --- | --- | --- | --- | --- | --- | --- | --- |
|  |  |  |  | **Fiber** | **Control** | **Fiber** | **Control** | **Fiber** | **Control** | **Fiber** | **Control** | **Fiber** | **Control** | **Fiber** | **Control** |
| [38] | Hart, 1988 (Australia) | Singlecenter | 68 (90 randomized); ICU, starting on enteral tube feeds | 47.1 ± 20.8 | 48.5 ± 18.6 | 22/13 | 21/12 | NR | NR | NR | NR | NR | NR | NR | NR |
| [39] | Dobb, 1990 (Australia) | Singlecenter | 91 (137 randomized); adult patients in the ICU | 47 ± 19 | 45 ± 19 | 29/16 | 32/14 | NR | NR | NR | NR | NR | NR | NR | NR |
| [47] | Celaya, 1992 (Spain) | Singlecenter | 35; ICU, with trauma or sepsis and stress diabetes, expected EN ≥ 14d | 48 ± 15 | 43 ± 12 | 5/12 | 7/11 | APACHE II: 23.8 ± 5.6 | APACHE II: 21.5 ± 4.7 | 9/17 (52.9) | 8/18 (44.4) | NR | NR | NR | NR |
| [35] | Caparrós, 2000 (Spain) | Multicenter; 15 | 220 (237 randomized); age ≥ 18, ICU, APACHE II ≥ 8, MOD ≤ 5, expected EN ≥ 7d | 51 (34-69) | 58 (37-69) | 91/31 | 92/30 | APACHE II: 17 (13-20) | APACHE II: 16 (13-21) | 118/122 (96.7) | 96/98 (98) | Medical 63/122 (52) Surgical 11/122 (9) Trauma 48/122 (39) | Medical 50/98 (51) Surgical 12/98 (12) Trauma 36/98 (37) | NR | NR |
| [41] | Schultz, 2000 (USA) | Singlecenter | 44 (80 randomized); age ≥ 18, ICU, antibiotics administration, need of EN | fiber + placebo 62.6 ± 14.9 fiber-free + pectin 72.8 ± 13.1 fiber + pectin 60.4 ± 16.1  pooled: 65.27 ± 14.98 | 66.5 ± 19.2 | 17/16  [fiber + placebo 6/5 fiber-free + pectin 5/6 fiber + pectin 6/5] | 7/4 | APACHE II:  fiber + placebo 17.3 ± 6.12 fiber-free + pectin 17.8 ± 4 fiber + pectin 15.4 ± 5.8 pooled: 16.83 ± 5.47  Acuity score: fiber + placebo 5.6 ± 0.52 fiber-free + pectin 5.5 ± 0.97 fiber + pectin 5.5 ± 0.69 pooled: 5.53 ± 0.76 | APACHE II: 17.9 ± 6.33  Acuity score: 5.4 ± 0.52 | NR | NR | Surgical 5/33 (15.2) Medical 16/33 (48.5) Trauma 4/33 (12.1) Neurological 7/33 (21.2) | Surgical 4/11 (36.4) Medical 2/11 (18.2) Trauma 2/11 (18.2) Neurological 2/11 (18.2) Other 1/11 (9.09) | NR | NR |
| [43] | Spapen, 2001 (Belgium) | Singlecenter | 25 (35 randomized); adult medical ICU, severe sepsis or septic shock | 68 ± 11 | 69 ± 15 | 7/6 | 6/6 | APACHE II: 26 ± 7 | APACHE II: 24 ± 8 | 13/13 (100) | 12/12 (100) | 100% medical | 100% medical | NR | NR |
| [40] | Rushdi, 2004 (Egypt) | Singlecenter | 20 (30 randomized); ICU, age ≥ 20, on EN with ≥ 3 liquid stools/day, APACHE II 16-22 | 53 ± 14 | 62 ± 12 | 6/4 | 5/5 | APACHE II: 18 ± 2 Sickness score: 7 ± 2 | APACHE II: 18 ± 2 Sickness score: 7 ± 2 | NR | NR | NR | NR | 70 ± 13 kg25.1 ± 3.1 kg/m² | 74 ± 11 kg 26 ± 2.5 kg/m² |
| [37] | Palmese, 2006 (Italy) | Singlecenter | 84; 18-65 years, ICU, MV, with central venous catheter and foley catheter in the bladder, antibiotics administration, expected survival > 48h (APACHE < 20) | 51 ± 13 | 50 ± 12 | 23/19 | 21/21 | APACHE II: 17.2 ± 5 | APACHE II: 16.9 ± 4 | 42/42 (100) | 42/42 (100) | Medical 29/42 (69) Surgical 9/42 (21.4) Trauma 3/42 (7.14) Intoxication 1/42 (2.38) | Medical 30/42 (71.4) Surgical 10/42 (23.8) Trauma 2/42 (4.76) | NR | NR |
| [49] | Karakan, 2007 (Turkey) | Singlecenter | 30; with severe acute pancreatitis | 47.3 ± 16.8 | 44.9 ± 11.2 | 6/9 | 8/7 | APACHE II: 9.4 ± 3.7 Balthazar CT score: 8.5 ± 4.6 | APACHE II: 9.6 ± 3.8 Balthazar CT score: 9.1 ± 5.2 | NR | NR | NR | NR | 24.7 ± 7.8 kg/m² | 27.1 ± 9.5 kg/m² |
| [46] | Spindler-Vesel, 2007 (Slovenia) | Singlecenter | 113 (132 randomized); surgical ICU, multiple injured patients with Injury Severity Score (ISS) > 18, ICU stay ≥ 4 days | 36 (22-51) | group 1: 31 (23-50) group 2: 41 (26-54) | NR | NR | APACHE II: 11 (4.5 - 18) | APACHE II:  group 1: 14 (11-18) group 2: 8 (4-15) | NR | NR | 100% surgical | 100% surgical | NR | NR |
| [45] | Chittawatanarat, 2010 (Thailand) | Singlecenter | 34; surgical ICU; septic patients with broad spectrum antibiotics and total EN | 49.2 ± 20.5 | 51.9 ± 17.4 | 6/11 | 6/11 | APACHE II: 19.8 ± 4.2 SAP II: 39.9 ± 8.5 | APACHE II: 20.2 ± 6.6 SAP II: 40.1 ± 8.6 | NR | NR | 100% surgical | 100% surgical | 55.1 ± 9.5 kg | 56.9 ± 15.3 kg |
| [52] | Zavertailo, 2010 (Russia) | Singlecenter | 56; 18-60 years, ICU, traumatic brain injury and haemorrhagic stroke, MV, expected MV duration ≥ 5 days | 45.4 ± 10.7 | 40.2 ± 12.2 | 25/3 | 21/7 | APACHE II: 7.5 (14-21) GCS: 7 (7-9) | APACHE II: 19 (11-20) GCS: 0 (7-13) | 28/28 (100) | 28/28 (100) | NR | NR | NR | NR |
| [53] | Aytünür, 2012 (Turkey) | Singlecenter | 60; ICU, MV, expected MV ≥ 10d | 44.63 ± 20.52 | 46.13 ± 28.50 | 9/21 | 16/14 | APACHE II: 19 (19-21) | APACHE II: 20 (17-26) | 30/30 (100) | 30/30 (100) | NR | NR | 62.23 ± 4 kg 23.43 ± 2.43 kg/m² | 65.17 ± 14.9 kg 22.73 ± 5.53 kg/m² |
| [51] | Wang, 2014 (China) | Singlecenter | 86; critically ill trauma patients | 40.12 ± 8.13 | 41.57 ± 8.76 | 30/13 | 28/15 | NR | NR | NR | NR | NR | NR | NR | NR |
| [36] | Kamarul Zaman, 2016 (Malaysia) | Singlecenter | 88; age ≥ 18, critically ill patients, ICU, expected EN ≥ 5 days | 56 (33) | 54.5 (28) | 21/14 | 23/10 | SOFA: 8 ± 6.5 SAPS2: 37.8 ± 16.4 | SOFA: 9 ± 4.5 SAPS2: 45.3 ± 16.2 | NR | NR | NR | NR | 23.9 (10.8) kg/m² | 24.3 (10.39) kg/m² |
| [44] | Yagmurdur, 2016 (Turkey) | Singlecenter | 120; 35-90 years, adult medical ICU, with acute cerebrovascular disease, MV, EN | 71 ± 14 | 70 ± 15 | 24/36 | 26/34 | APACHE II: 15.7 ± 2.9 | APACHE II: 15.7 ± 2.6 | 60/60 (100) | 60/60 (100) | 100% medical | 100% medical | 76 ± 10.6 kg 27 ± 4.5 kg/m² | 76.7 ± 9.8 kg 27.2 ± 3.7 kg/m² |
| [54] | Xi, 2017 (China) | Singlecenter | 125 (166 randomized); age ≥ 18, ICU | 48.2 ± 13.7 | 48.7 ± 10.7 | 35/28 | 36/26 | APACHE II: 12 ± 2.36 SOFA: 8.5 ± 2.8 | APACHE II: 12.3 ± 2.75 8.4 ± 3.0 | 53/63 (84.1) | 50/62 (80.7) | NR | NR | 22 ± 2.28 kg/m² | 22.1 ± 1.58 kg/m² |
| [50] | Fazilaty, 2018 (Iran) | Singlecenter | 40 (68 randomized); age ≥ 18, MV, ≥ 2 organ-system traumas | 43 (29-53) | 32 (25.5-43) | 18/2 | 18/2 | APACHE III: 62 (56.25-67) GCS: 7 (5-7) | APACHE III: 62 (53.25-64.75) GCS 6 (5-7) | 20/20 (100) | 20/20 (100) | NR | NR | NR | NR |
| [42] | Freedberg, 2020 (USA) | Singlecenter | 20 (22 randomized); age ≥ 18, medical ICU, with sepsis, received a broad-spectrum IV antibiotics within the previous 24h, expected EN duration ≥ 3d | < 50 years: 4/10 (40) 50-70 years 4/10 (40) > 70 years 2/10 (20) | < 50 years: 3/10 (30) 50-70 years 3/10 (30) > 70 years 4/10 (40) | 5/5 | 7/3 | SOFA: ≤ 6 points: 2/10 (20) 7-10 points: 5/10 (50) ≥ 11 points: 3/10 (30)  GCS: 15 points 3/10 (30) 10-14 points 3/10 (30) < 10 points 4/10 (40) | SOFA: ≤ 6 points: 5/10 (50) 7-10 points: 3/10 (30) ≥ 11 points: 2/10 (20)  GCS: 15 points 5/10 (50) 10-14 points 4/10 (40) < 10 points 1/10 (10) | NR | NR | 100% medical | 100% medical | NR | NR |
| [48] | Chen, 2021 (China) | Singlecenter | 46 (49 randomized); age 18-70, ICU, with severe acute pancreatitis | 45 ± 9.07 | 51.1 ± 12 | 11/11 | 11/13 | APACHE II: 18.5 ± 7.71 Balthazar CT Score: 6 (4.5-8) Modified Marshall score: 3 (2-3) SOFA: 4.5 (4-6.75) | APACHE II: 18.6 ± 5.65 Balthazar CT Score: 6 (5.75-8) Modified Marshall score: 3 (2-5) SOFA: 6 (4-8) | NR | NR | NR | NR | 70 (63.8-80) kg | 70 (65-80) kg |

APACHE II: acute physiology and chronic health evaluation II, BMI: body mass index, ICU: intensive care unit, Med: medical, MV: mechanical ventilation, N: number of center, n: sample size, SAPS II: simplified acute physiology score II, GCS: Glasgow Coma Scale, SOFA: sequential organ failure assessment

### Table S2: Funding source

| **Reference No** | **Author, year** | **industry/Non-industry/Unclear/None** | **Grant/Company name** | **additional information** |
| --- | --- | --- | --- | --- |
| [38] | Hart, 1988 | Industry | Reckitt & Coleman Australia | supplied Fybogel and placebo |
| [39] | Dobb, 1990 | Unclear |  |  |
| [47] | Celaya, 1992 | Unclear |  |  |
| [35] | Caparrós, 2000 | Industry | Nutricia Spain, S.A |  |
| [41] | Schultz, 2000 | Industry | 1. Sigma Theta Tau International 2. Glaxo-Wellcome |  |
| [43] | Spapen, 2001 | Industry | Novartis Nutrition, the Netherlands | provided enteral feeds |
| [40] | Rushdi, 2004 | Industry | Novartis Nutrition GmbH, München, Germany |  |
| [37] | Palmese, 2006 | Unclear |  |  |
| [49] | Karakan, 2007 | Unclear |  |  |
| [46] | Spindler-Vesel, 2007 | Non-industry | Ministry of Science of Republic of Slovenia |  |
| [45] | Chittawatanarat, 2010 | Industry | Nestlé Ltd. | donated both enteral diet formulas free of charge, no financial support |
| [52] | Zavertailo, 2010 | Unclear |  |  |
| [53] | Aytünür, 2012 | Unclear |  |  |
| [51] | Wang, 2014 | Unclear |  |  |
| [36] | Kamarul Zaman, 2016 | Non-industry | University of Malaya |  |
| [44] | Yagmurdur, 2016 | Unclear |  |  |
| [54] | Xi, 2017 | Non-industry | 1. National Natural Science Foundation of China 2. Jiangsu Province Special Program of Medical Science 3. Jiangsu Province's Key Medical Talent Program 4. Scientific Research Foundation of Graduate School of Nanjing University |  |
| [50] | Fazilaty, 2018 | Non-industry | National Nutrition and Food Technology Research Institute |  |
| [42] | Freedberg, 2020 | Non-industry | Feldstein Medical Foundation and Columbia University's Irving Institute |  |
| [48] | Chen, 2021 | Unclear |  |  |

### Table S3: Feeding information

| **Reference No** | **Author, year** | **EN formula in intervention group** | | | | **EN formula in control group** | | **dosage and frequency of EN** | **Route of administration** | **Timing of start of EN/intervention** | **Duration of EN/intervention** |
| --- | --- | --- | --- | --- | --- | --- | --- | --- | --- | --- | --- |
|  |  | **Brand and company** | **description** | **Type and content of fiber(s)** | **co-intervention (if any)** | **Brand and company** | **description** |  |  |  |  |
| [38] | Hart, 1988 | Osmolite (Abbott Laboratories, North Chicago, USA); "Fybogel" (Reckitt & Colman) | standard formula + fiber | Plantago ovata (Ispaghula husk): 7g/d | No | Osmolite (Abbott Laboratories, North Chicago, USA); "Weetbix" (Sanitarium, Australia) | standard formula + placebo (wheatbased breakfast cereal) | 1. day: 40ml/h of half-strength feed 2. day: 40ml/h of full-strength feed ≥ 3. day: increased by 20-40ml/h/d depending on clinical assessment, to a maximum of 120ml/h or more | nasogastric tube | NR | ≥ 3 days |
| [39] | Dobb, 1990 | Enrich (Abbott Australia Pty Ltd.) | iso-osmolar, lactose-free, fiber-enriched | soy polysaccharide: 21g/l | No | Ensure (Abbott Australia Ptd Ltd.) | standard formula, iso-osmolar, lactose-free (fiber-free) | 1. day: 40ml/h of half-strength feed 2. day: 40ml/h of full-strength feed ≥ 3. day: increased by 20-40ml/h/day depending on clinical assessment, to a maximum of 120ml/h | nasogastric tube | NR | ≥ 3 days until max. 18 days or discharge from ICU |
| [47] | Celaya, 1992 | Glucerna (Abbott Laboratories) | diet specific for glucose intolerance | soy polysaccharides: 14.4g/1000cal | No | NR | hyperproteic diet without fiber | NR | fine caliber tube (not specified) | NR | ≤ 14 days |
| [35] | Caparrós, 2000 | Stresson Multifibre (Nutricia Spain S.A., Madrid, Spain) | high-protein formula with fiber, arginine, medium-chain triglycerides, antioxidants | soy polysacharides: 4.2g/1500ml cellulose: 1.65g/1500ml resistant starch: 1.2g/1500ml inuline: 1.65g/1500ml oligofructose: 1.5g/1500ml arabic gum: 3.3g/1500ml | arginine: 11.8%; 10.05g/1500ml medium-chain triglycerides: 40%; 25.8g/1500ml Vitamin A: 1995µg/1500ml Vitamin C: 200mg/1500ml Vitamin E: 73.8mg/1500ml | Nutrison Protein Plus (Nutricia Spain S.A., Madrid, Spain) | high-protein standard diet (fiber-free) | 24h at a constant rate by infusion pump; 42ml/h the first day; increase of volume of 20ml/h every 12 hours until caloric goal (25kcal/kg/day) was achieved | Enteral-gastric or enteral-jejunal tube | within 48 hours of admission | 10 (6-18) days |
| [41] | Schultz, 2000 | fiber-enriched: Jevity Plus (NR)   pectin: NR | fiber-enriched formula + placebo fiber-free formula + pectin fiber-enriched formula + pectin | fiber-enriched formula: 25% soluble fiber (not specified)  pectin (100% soluble fiber): 1.07g/d  mean daily fiber in fiber/placebo group: 17.3g mean daily fiber in fiber/pectin group: 15.8g | No | Osmolite (Abbott, Illinois, USA) or Promote (Abbott Nutrition, Chicago, USA) | standard fiber-free formula + placebo | NR | feeding tube (not specified) | 5.8 ± 4.4; 4.6 ± 1.7; 4.7 ± 2.2 days | 6 days |
| [43] | Spapen, 2001 | Benefiber (Novartis Nutrition, the Netherlands) | isocaloric, isonitrogenous EN with fiber | partially hydrolyzed guar gum: 22g/l | No | NR | standard isocaloric, isonitrogenous control formula without fiber | 25ml/h for the first 24h, then increased by 25-35ml/h until at least 80% of individual energy needs were reached | nasogastric tube | within 24h after randomization | 11 ± 4 (≥ 6 days, max. 21 days or withdrawal of EN) |
| [40] | Rushdi, 2004 | Sandosource GI Control (Novartis Nutrition GmbH, München, Germany) + Benefiber (NR) | fiber-enriched feed | soluble guar gum: 2%, 22g/l (22-24g/day) | No | Propeptide (Prime, Nutrition Medical, Inc. USA) | standard fiber-free feed | energy administration (25-23kcal/kg/day) during 18-24h per 24h at a constant rate through pump-assisted system; 50% of required energy intake on first day, 75% on second day, 100% on third + fourth day | nasojejunal tube | NR | 4 days |
| [37] | Palmese, 2006 | Jevity (Abbott, Zwolle, The Netherlands) | isocaloric, isonitrogenous EN with fiber | Soluble and insoluble dietary fiber: 10.6g/l (fructooligosaccharides: 7g/l) | i.v. glutamine (both groups): 10g/day | NR | isocaloric, isonitrogenous EN without fiber + i.v. glutamine administration | all patients: bolus of 50ml of EN, both EN and PN were administered to reach target caloric intake (25-23kcal/kg/day) intervention: 2x for 7x hours/day with 5 hours break, overall 1000ml/day | nasogastric tube | within 24h of admission | duration of ICU stay |
| [49] | Karakan, 2007 | NR | calorie-, lipid- and protein-identical to control formula + multifibers | soluble fibers (not specified): 0.7g/100ml insoluble fibers (not specified): 0.8/100ml total: 1.5g/100ml, 24g/d | No | NR | standard calorie-, lipid- and protein-identical without fiber | 30ml/h at full strength, increasing to 100ml/h over 24-48h, caloric goal 2000kcal/d all patients received adjuvant peripheral parenteral nutrition with standard solution | nasojejunal tube | within 24h of admission | 8 ± 4 (6-12) |
| [46] | Spindler-Vesel, 2007 | Nova Source (Novartis Medical Nutrition, Basel, Switzerland) | EN with fiber | fermentable guar gum: 22g/l | No | group A: Alitraq (Abbott-Ross, Abott Park, IL, USA) group C: Nutricomp peptide (B. Braun, Melsungen, Germany) | group A: EN with glutamine, arginine, linolenic acid group C: peptide diet | 30ml/h for 4 hours, interruption for 2 hours to assess gastric intolerance, if volume < 200ml the rate was increased by 50-100%, maximum volume 160ml/h, EN was stopped during 6 night hours, target value: 0.2-0.3 gN/kg/d and 25 nonprotein kcal/kg/day at 72h of admission | intragastric tube | within 24h after injury; intervention: 12.5 (9.6-15); control group A: 15.5 (13-20.3); control group C: 12.8 (10-18) | NR |
| [45] | Chittawatanarat, 2010 | Nutren Fibre (Nestlé Suisse S.A., Switzerland) | mixed fiber formula | overall dietary fiber, produced by yellow pea fiber + fructo-oligosaccharide: 15.1 g/l; soluble:insoluble fiber 1:1;  soluble fiber: 35% fructo-oligosaccharide, 15% pectin; insoluble fiber: 30% cellulose, 5% lignin, 15% hemicellulose | No | Nutren Optimum (Nestlé Suisse S.A., Switzerland) | standard formula (fiber-free) | NR | feeding tube (not specified) | NR | all patients ≥ 5 days, 8/17 vs. 10/17 14 days (≥ 5 days until max. 14 days or change to normal oral diet) |
| [52] | Zavertailo, 2010 | Nutricomp Intensive (Braun, Germany) + Nutricomp ADN Braun Fiber (Braun, Germany) | fluid-restricted formula + fiber-formula | NR | Erythromycin: 300mg first 3 days | NR | standard formula (isocaloric, no fiber), no erythromycin | 4x/day with 1h breaks, total feeding duration 18-20h/day; first day: 50ml/h, increase of rate each subsequent day by 25ml/h, maximum rate 125ml/h | nasogastric tube | NR | NR |
| [53] | Aytünür, 2012 | Jevity (Abbott, Zwolle, The Netherlands) | standard fiber iso-osmolar formula | Soluble and insoluble dietary fiber: 10.6g/l (fructooligosaccharides: 7g/l) | No | Osmolite (Abbott, Illinois, USA) | standard iso-osmolar formula (fiber-free) | initial 30ml/h; increase by 20ml/h every 8 hours in patients without complications; no enteral feeding between 24:00 and 08:00 | nasogastric tube | NR | 3 days |
| [51] | Wang, 2014 | fiber: (Haiyishengyuan Biological Engineering Co., Ltd.) | standard EN + fiber + glutamine | soluble dietary fiber (not specified): 3g/1000kcal | glutamine: 0.5g/kg/day | NR | standard EN | daily energy supply was calculated using a formula | nasogastric tube | NR | NR |
| [36] | Kamarul Zaman, 2016 | Ensure FOS (NR) | EN with fiber | fructooligosaccharide: 10g/l; 14.8g/d | No | Osmolite (Abbott, Illinois, USA) | standard EN | volumes of EN were based on each patient's total energy requirement, which was calculated by attending dietitian | nasogastric tube | 0 ± 1 vs. 0 ± 1.5 days | 14 days |
| [44] | Yagmurdur, 2016 | Nutrison multifibre (500ml, Nutricia Advanced Medical Nutrition, Netherlands) | fiber-enriched solution | soluble fibers: 7g/l, insoluble fibers: 8g/l soy polysaccharides: 35% arabic gum: 24% inulin: 12.5% alpha-cellulose: 12% oligofructose: 10.5% resistant starch: 9% daily fiber dose: 28g/d | No | Nutrison (500ml, Nutricia Advanced Medical Nutrition, Netherlans) | standard fiber-free solution | target value: 25-35kcal/kg/day; EN 18-24h/day at constant rate; first day: 50% of required energy intake, second day: 75%, 3. day: 100%; EN startes at 20ml/h, increased at six-hour intervals to 40ml/h, 80ml/h,100-120ml/h | nasogastric tube | within 48h after admission | NR |
| [54] | Xi, 2017 | Peptisorb (NR) | control formula + pectin | pectin: 6g each time, 24g/day | No | Peptisorb (NR) | standard EN | both groups: 1. day 5% glucose at 25ml/; 2. day EN (31.3g Peptisorb dissolved in 250ml water) at 12.5ml/h; 3.-6. day EN (62.5g Peptisorb dissolved in 250ml) at a rate of 12.5ml/h; from 7. day EN was advanced to the goal energy target (25kcal/kg/day) as quickly as possible fiber group: additional amount of pectin was administrated once 4 hours ahead of EN from day 2-6 (24g every day) | nasojejunal tube | within 36h after admission | ≥ 6 days |
| [50] | Fazilaty, 2018 | ß-glucan: (Arian Salamat Sina Company, Teheran, Iran) | hospital-prepared standard kitchen formula + ß-glucan | oat ß-glucan: 3g/d | No | None (hospital-prepared) | hospital-prepared standard kitchen formula + placebo (3g/d maltodextrin) | NR | feeding tube (not specified) | within 24-48h of admission | ≥ 10 days until 21 days or discharge from ICU |
| [42] | Freedberg, 2020 | Promote 1.0 with Fiber (Abbott Nutrition, Chicago, USA) | mixed fiber formula, calorie- and micronutrient-identical to control formula | oat and soy derived dietary fiber (not specified): 14.3g/l  median dose: 11g/d | No | Promote 1.0 (Abbott Nutrition, Chicago, USA) | standard formula, calorie- and micronutrient identical | NR | feeding tube (not specified) | NR | ≥ 3 days until withdrawal from study or hospital discharge or day 30 or death |
| [48] | Chen, 2021 | polydextrose: (Tailijie Biotech Co, Ltd, Henan, China) | Control formula + polydextrose | polydextrose (soluble dietary fiber): 20g/day | No | NR | Control formula without fiber | 1. day (first 24 hours): EN volume 250ml (= 250kcal), speed 30 ml/h  2. day (24-48 hours): EN volume 500-750ml (=500-750 kcal), speed 40-50ml/h  3. day (48-72 hours): EN volume 750-1250ml (=750-1250 kcal), speed 60-80ml/h  > 72 hours: EN volume 1250-2250ml (= 1250-2250 kcal = caloric goal), speed 80-120ml/h | nasojejunal tube | NR | NR |

### Table S4: Clinical outcomes

| **Reference No** | **Author, year** | **Mortality n (%)** | | **Gastrointestinal complications (not including diarrhea)** | | **Length of stays (days, mean ± SD)** | | **Duration of Ventilation (days, mean ± SD)** | | **Infectious complications n (%)** | |
| --- | --- | --- | --- | --- | --- | --- | --- | --- | --- | --- | --- |
|  |  | **Fiber** | **Control** | **Fiber** | **Control** | **Fiber** | **Control** | **Fiber** | **Control** | **Fiber** | **Control** |
| [38] | Hart, 1988 | NR | NR | **abdominal distension** 2/35 (5.71)  **ileus or gastric stasis** 4/35 11.4) | **abdominal distension** 0/33  **ileus or gastric stasis** 3/33 (9.09) | NR | NR | NR | NR | NR | NR |
| [39] | Dobb, 1990 | NR | NR | NR | NR | NR | NR | NR | NR | NR | NR |
| [47] | Celaya, 1992 | **Unspecified** 4/17 (22.2) | **Unspecified** 5/18 (29.4) | **gastric distension** 2/17 (11.8) **constipation** 3/17 (17.6) | NR | NR | NR | NR | NR | NR | NR |
| [35] | Caparrós, 2000 | **ICU** 19/122 (15.6)  **In-hospital** 25/122 (20.5)  **6-month** 30/122 (24.6) Medical 17/63 (27) Surgical 4/11 (36.4) Trauma 9/48 (18.8) | **ICU** 21/98 (21.4)  **In-hospital** 29/98 (29.6)  **6-month** 31/98 (31.6) Medical 19/63 (38) Surgical 5/11 (41.7) Trauma 7/48 (19.4) | *incidence density rates: number of episodes per 1000 days of enteral nutrition*  **Bronchial aspiration of gastric contents** 0.7 **Increased gastric residual** 100.1 **Vomiting** 8.7 **Constipation** 4.7 **Abdominal distension** 9.3 | *incidence density rates: number of episodes per 1000 days of enteral nutrition*  **Bronchial aspiration of gastric contents** 0.9 **Increased gastric residual** 53.7 **Vomiting** 11.4 **Constipation** 15.9 **Abdominal distension** 11.4 | **ICU** 15 (9.8-25) **Hospital** 29 (16.8-51) | **ICU** 13 (8.8-20.3) **Hospital** 26 (17.8-42) | 10 (5-18) | 9 (5-14) | *Infection density rates: number of episodes per 1000 days of ICU LOS* **Overall infections** 37.1 **Bacteremia** 6.8 **Catheter-realted sepsis** 0.4 **Surgical infections** 1.9 **Urinary tract infections** 6.8  *Infection density rate: number of episodes per 1000 days of mechanical ventilation* **Nosocomial pneumonia** 32.2 | *Infection density rates: number of episodes per 1000 days of ICU LOS* **Overall infections** 35.1 **Bacteremia** 6.7 **Catheter-realted sepsis** 5.5 **Surgical infections** 1.8 **Urinary tract infections** 3.0  *Infection density rate: number of episodes per 1000 days of mechanical ventilation* **Nosocomial pneumonia** 24.7 |
| [41] | Schultz, 2000 | NR | NR | NR | NR | **ICU**: **fiber + placebo** 20.7 ± 8.5 **fiber-free + pectin** 17.3 ± 8.2 **fiber + pectin** 22.1 ± 16.4 **Pooled:** 20.03 ± 11.85  **Hospital**: **fiber + placebo** 42.8 ± 32.5 **fiber-free + pectin** 24.4 ± 9 **fiber + pectin** 33.8 ± 22.1 **Pooled**: 33.67 ± 23.64 | **ICU** 28 ± 14.6 **Hospital** 34 ± 14.7 | NR | NR | NR | NR |
| [43] | Spapen, 2001 | **Hospital** 1/13 (7.7) | **Hospital** 4/12 (33.3) | NR | NR | 19 (11-51) | 17 (10-30) | 11 ± 4 | 12 ± 5 | NR | NR |
| [40] | Rushdi, 2004 | NR | NR | **number of liquid stools:** first day 2 ± 0.9 fourth day 1 ± 0.7  **severe nausea** 0/10 **vomiting** 0/10 **flatulence** 2/10 (20) **constipation** 0/10 | **number of liquid stools:** first day 1.2 ± 0.7 fourth day 2.1 ± 0.8  **severe nausea** 0/10 **vomiting** 2/10 (20) **flatulence** 4/10 (40) **constipation** 1/10 (10) | NR | NR | NR | NR | NR | NR |
| [37] | Palmese, 2006 | **Unspecified** 6/42 (14.2) | **Unspecified** 8/42 (19.0) | **patients with gastrointestinal complications:** 11/42 (26.2)   **Increase in gastric residua**l 5/42 (11.9) **Regurgitation** 2/42 (4.76) **Vomiting** 5/42 (11.9) **Constipation** 1/42 (2.38) **Abdominal distension** 5/42 (11.9) | **patients with gastrointestinal complications:** 15/42 (35.7)   **Increase in gastric residual** 6/42 (14.3) **Regurgitation** 3/42 (7.14) **Vomiting** 4/42 (9.52) **Constipation** 1/42 (2.38) **Abdominal distension** 6/42 (14.3) | **ICU** 12 ± 4.6 | **ICU** 13 ± 3.4 | 6 ± 1.7 | 5 ± 2.5 | **Bacteremia** 2/42 (4.76) **Pneumonia** 2/42 (4.76) **urinary tract infection** 6/42 (14.3) **central line infection** 2/42 (4.76) **soft tissue infection** 0/42 **intrathoracic and/or intra-abdominal infection** 1/42 (2.38)  > 1 infection: 3/42 (7.1) | **Bacteremia** 4/42 (9.52) **Pneumonia** 6/42 (14.3) **urinary tract infection** 5/42 (11.9) **central line infection** 5/42 (11.9) **soft tissue infection** 0/42 **intrathoracic and/or intra-abdominal infection** 1/42 (2.38)  > 1 infection: 6/42 (14.3) |
| [49] | Karakan, 2007 | **Unspecified** 2/15 (13.3) | **Unspecified** 4/15 (26.7) | **bloating and gas symptoms** 3/15 | **bloating and gas symptoms** 0/15 | **ICU** 6 ± 2 (5-8) **Hospital** 10 ± 4 (8-14) | **ICU** 6 ± 2 (5-7) **Hospital** 15 ± 6.0 (7-26) | NR | NR | **cholangitis** 1/15 (6.67) **sepsis** 1/15 (6.67) | **cholangitis** 0/15 **sepsis** 2/15 (13.3) |
| [46] | Spindler-Vesel, 2007 | **ICU** 2/29 (6.90) | **ICU** 3/58 (5.17) [group A 1/32 (3.13)] [group C 2/26 (7.69)] | NR | NR | 16 (10-21) | group A: 14 (8.3-23) group C: 11.5 (6-20) | 10 (6-16) | group A: 12 (8-15) group C: 8 (4-15) | **pneumonia** 12/29 (41.4) **urinary tract** 0/29 **vascular** 1/29 (3.45) **wound** 2/29 (6.90) **positive hemocultures** 2/29 (6.90) **others** 0/29 | **pneumonia** 22/58 (37.9) [group A 11/32 (34.4), group C 11/26 (42.3)] **urinary tract** 1/58 [group A 1/32 (3.13), group C 0/26] **vascular** 1/58 (1.72) [group A 1/32 (3.13), group C 0/26] **wound** 3/58 (5.17) [group A 1/32 (3.13), group C 2/26 (7.69) **positive hemocultures** 1/58 (1.72) [group A 1/32 (3.13), group C 0/26] **others** 1/58 [group A 1/32 (3.13, group C 0/26] |
| [45] | Chittawatanarat, 2010 | **Unspecified** 1/17 (5.88) | **Unspecified** 2/17 (11.8) | NR | NR | **surviving patients: ICU** 16.8 ± 8 (6-37) **Hospital** 30.9 ± 28 (6-120) | **surviving patients: ICU** 25.5 ± 13 (11-50) **Hospital** 36.1 ± 14.8 (15-61) | NR | NR | NR | NR |
| [52] | Zavertailo, 2010 | **30-day** 3/28 (10.7) | **30-day** 3/28 (10.7) | NR | NR | **ICU** 25.8 ± 14 | **ICU** 32.6 ± 25.4 | 20.3 ± 11 | 25.8 ± 20.2 | NR | NR |
| [53] | Aytünür, 2012 | NR | NR | **Abdominal distension** 1. day 4/30 (13.33) 2. day 7/30 (23.33) 3. day 3/30 (10)  **Vomiting** 1. day 0/30 2. day 2/30 (6.66) 3. day 3/30 (10)  **Regurgitation** 1. day 1/30 (3.33) 2. day 8/30 (26.6) 3. day 6/30 (20)  **Aspiration** 0/30  **≥ 1 complication**: 20/30 (66.66) | **Abdominal distension** 1. day 4/30 (13.33) 2. day 2/30 (6.66) 3. day 3/30 (10)  **Vomiting** 1. day 0/30 2. day 2/30 (6.66) 3. day 4/30 (13.33)  **Regurgitation** 1. day 0/30 2. day 8/30 (26.6) 3. day 6/30 (20)  **Aspiration** 0/30  **≥ 1 complication**: 17/30 (56.66) | NR | NR | NR | NR | NR | NR |
| [51] | Wang, 2014 | NR | NR | **constipation** 6/43 (13.95) **nausea** 0/43 **vomiting** 0/43 **digestive bleeding** 0/43 | **constipation** 13/43 (30.23) **nausea** 0/43 **vomiting** 0/43 **digestive bleeding** 0/43 | NR | NR | NR | NR | NR | NR |
| [36] | Kamarul Zaman, 2016 | NR | NR | NR | NR | NR | NR | NR | NR | NR | NR |
| [44] | Yagmurdur, 2016 | NR | NR | **Regurgitation** 10/60 (16.7) **Vomiting** 9/60 (15) **Distension** 25/60 (41.7) **Constipation** 2/60 (3.33) | **Regurgitation** 9/60 (15) **Vomiting** 10/60 (16.7) **Distension** 18/60 (30) **Constipation** 2/60 (3.33) | NR | NR | NR | NR | NR | NR |
| [54] | Xi, 2017 | **30-day** 1/62 (1.61) | **30-day** 3/63 (4.8) | **Gastrointestinal intolerance** 17/62 (27.4)  **vomiting** 2/62 (3.2) **abdominal distension or cramping** 4/62 (6.5) **constipation** 2/62 (3.2) **Regurgitation** 3/62 (4.8) | **Gastrointestinal intolerance** 26/63 (41.3)  **vomiting** 3/63 (4.8) **abdominal distension or cramping** 5/63 (7.9) **constipation** 7/63 (11.1) **Regurgitation** 5/63 (7.9) | **ICU** 13.8 ± 8.59 **Hospital** 23.4 ± 13.2 | **ICU** 17.9 ± 9.72 **Hospital** 32.9 ± 19 | NR | NR | 7/62 (11.3) | 9/63 (14.3) |
| [50] | Fazilaty, 2018 | **Unspecified** 1/20 (5.00) | **Unspecified** 4/20 (20.0) | NR | NR | **ICU** 27.55 ± 7.8 | **ICU** 31.2 ± 15.8 | 15 (10-22.5) | 28 (11-39.75) | **infection rate** 5/20 (25) **ventilator associated pneumonia** 4/20 (20.0) **urinary tract** 0/20 **wound** 2/20 (10.0) **sepsis** 0/20 **central nervous system** 0/20 | **infection rate** 11/20 (55) **ventilator associated pneumonia** 4/20 (20.0) **urinary tract** 4/20 (20.0) **wound** 4/20 (20.0) **sepsis** 2/20 (10.0) **central nervous system** 3/20 (15.0) |
| [42] | Freedberg, 2020 | **Unspecified** 2/10 (20.0) | **Unspecified** 4/10 (40.0) | NR | NR | NR | NR | NR | NR | **culture-proven infections 3/10 (30.0)** | **culture-proven infections** 3/10 (30.0) |
| [48] | Chen, 2021 | **28-day** 0/24 (0) | **28-day** 1/22 (4.55) | **Feeding intolerance** 6/24 (25) **Abdominal distension** 7/24 (29.2) **Vomiting** 2/24 (8.33) **Constipation** 3/24 (12.5) **Gastrointestinal bleeding** 2/24 (8.33) | **Feeding intolerance** 13/22 (59.1) **Abdominal distension** 16/22 (72.7) **Vomiting** 2/22 (9.09) **Constipation** 16/22 (72.7) **Gastrointestinal bleeding** 1/22 (4.55) | **ICU** 10 (6.25-13.8) **Hospital** 17.5 (15-26) | **ICU** 13 (10.5-16-8) **Hospital** 17.5 (15-26) | NR | NR | **urinary tract infection** 0/24 **intra-abdominal infection** 1/24 (4.17) **systemic infection** 1/24 (4.17) **intravascular catheter-related infection** 2/24 (8.33) | **urinary tract infection** 2/22 (9.09) **intra-abdominal infection** 3/22 (13.6) **systemic infection** 0/22 **intravascular catheter-related infection** 1/22 (4.55) |

### Table S5: Metabolic and nutritional outcomes

| **Reference No** | **Author, year** | **Metabolic outcomes (Blood glucose, triglyceride)** | | **Nutritional indices** | |
| --- | --- | --- | --- | --- | --- |
|  |  | **Fiber** | **Control** | **Fiber** | **Control** |
| [38] | Hart, 1988 | NR | NR | **mean daily feed volumes:** 1537ml **1. day** 688 ± 204 **10. day** 2066 ± 463 | **mean daily feed volumes**: 1605ml **1. day** 628 ± 225 **11. day** 2010 ± 536 |
| [39] | Dobb, 1990 | NR | NR | **feed volumes** 1. day 380 ± 172ml | **feed volumes** 1. day 494 ± 265ml |
| [47] | Celaya, 1992 | **blood glucose (mg/dl):** overall mean 156.34 ± 24  **serum triglyceride (mg/dl):** 1. day 154 ± 45 7. day 148 ± 36 14. day 137 ± 22 | **blood glucose (mg/dl):** overall mean 191.83 ± 46  **serum triglyceride (mg/dl):** 1. day 139 ± 28 7. day 154 ± 39 14. day 176 ± 54 | NR | NR |
| [35] | Caparrós, 2000 | NR | NR | **administered caloric intake day 3** (kcal/day): 1625 (1137-1828)   **administered caloric intake day 7** (kcal/day): 1538 (1169-1863) | **administered caloric intake day 3** (kcal/day): 1625 (1314-1897)   **administered caloric intake day 7** (kcal/day): 1750 (1259-1963) |
| [41] | Schultz, 2000 | NR | NR | NR | NR |
| [43] | Spapen, 2001 | NR | NR | **time to reach preconceived protein/caloric goals:** 5 ± 3 days | **time to reach preconceived protein/caloric goals**: 6 ± 3 days |
| [40] | Rushdi, 2004 | **plasma glucose at end of study (day 5) (mg/dl):** 126 ± 81 (vs. Day 1 333 ± 208)  **serum cholesterol (mg/dl) at end of study (day 5):** 164 ± 71 (vs. Day 1 378 ± 26) | NR | **feed volumes 1. day:** 1070 ± 221 ml  **feed volumes 4. day:** 1775 ± 450 ml | **feed volumes 4. day:** 1070 ± 604 ml |
| [37] | Palmese, 2006 | NR | NR | NR | NR |
| [49] | Karakan, 2007 | NR | NR | NR | NR |
| [46] | Spindler-Vesel, 2007 | NR | NR | **average GRV first week:** 740 (530-1510) ml  **volume of EN first 24h:** 430 (100-600) ml  **total volume of EN during first 4 days:** 3250 (2400-3700) ml | **average GRV first week**:  group A: 410 (382-1062) ml group C: 620 (337-1190) ml  **volume of EN first 24h:** group A: 250 (157-562) ml group C: 387 (87-740)  **total volume of EN during first 4 days:** group A: 2720 (2356-3700) ml group C: 2675 (1515-3908) ml |
| [45] | Chittawatanarat, 2010 | NR | NR | **mean caloric delivery of 1500kcal:** achieved at day 6 | **mean caloric delivery of 1500kcal:** achieved at day 8 |
| [52] | Zavertailo, 2010 | NR | NR | **caloric delivery (kcal/kg/day) on 10. day:** 31.8 ± 10.5  **energy balance (kcal/day) on 10. day:** -404 ± 1007  **nitrogen balance (g/day) on 10. day:** -5.55 ± 9.61 | **caloric delivery (kcal/kg/day) on 10. day:** 20.6 ± 10.1  **energy balance (kcal/day) on 10. day:** -1359 ± 1078  **nitrogen balance (g/day) on 10. day:** -10.3 ± 7.9 |
| [53] | Aytünür, 2012 | NR | NR | **time to reach target calories:** 4 (3-6) days  **Gastric residual volume** (ml): 1. day  8:00 32.17 ± 47.61 16:00 57.67 ± 85.92 24:00 58.33 ± 69.04 2. day 8:00 54.33 ± 81.32 16:00 43 ± 97.49 24:00 47.83 ± 116.07 3. day 8:00 56.83 ± 92.80 16:00 60.83 ± 88.7 24:00 85.67 ± 168.84 | **time to reach target calories:** 4 (3-6) days  **Gastric residual volume** (ml) 1. day  8:00 34.76 ± 44.16 16:00 47.5 ± 53.27 24:00 48 ± 69.74 2. day 8:00 46.33 ± 63.87 16:00 48.83 ± 69.74 24:00 45 ± 72.08 3. day 8:00 35.33 ± 67.09 16:00 44.33 ± 64.82 24:00 30.5 ± 49.49 |
| [51] | Wang, 2014 | NR | NR | NR | NR |
| [36] | Kamarul Zaman, 2016 | NR | NR | **nutritional intake (kcal/day):** 1465.2 ± 337.4 | **nutritional intake (kcal/day):** 1501.7 ± 396.7 |
| [44] | Yagmurdur, 2016 | NR | NR | **daily GRV**: 1. day 113 ± 96 2. day 128 ± 116 3. day 126 ± 98 4. day 129 ± 100 5. day 122 ± 93  **mean daily volume ratio (%):** 84.2 ± 9.8 | **daily GRV**: 1. day 138 ± 100 2. day 136 ± 115 3. day 114 ± 91 4. day 117 ± 114 5. day 97 ± 72  **mean daily volume ratio (%):** 80.8 ± 9.6 |
| [54] | Xi, 2017 | **Episodes of hypoglycemia** 13/63 (20.6) | **Episodes of hypoglycemia** 5/62 (8.06) | **time to reach full EN**: 9.99 ± 1.91 days | **time to reach full EN**: 13 ± 5.12 days |
| [50] | Fazilaty, 2018 | NR | NR | **energy intake** 1710.5 ± 117 kcal | **energy intake** 1718.2 ± 182.4 kcal |
| [42] | Freedberg, 2020 | NR | NR | **% energy requirement achieved (day 3)**: 58 (24-84) | **% energy requirement achieved (day 3):** 33 (2-52) |
| [48] | Chen, 2021 | **Blood glucose** (mmol/l):  day 1 12.1 ± 1.8 day 4 9.7 ± 0.99 day 7 9.11 ± 1.11 | **Blood glucose** (mmol/l):  day 1 12.1 ± 1.57 day 4 11.9 ± 1.17 day 7 11.5 ± 1.39 | **time to achieve the energy target with EN**: 5 (4.25-6) days  **amount of energy received (kcal/day**): day 1: 250 (mean) day 4: 1500 (1000-1500) day 7: 1750 (1625-1937) | **time to achieve the energy target with EN**: 7 (6-8.25) days  **amount of energy received (kcal):** day 1: 250 (mean) day 4: 1000 (750-1000) day 7: 1500 (1187-1750) |

### Table S6: Diarrheal outcome

| **Reference No** | **Author, year** | **Definition** | **Fiber n (%)** | **Control n (%)** |
| --- | --- | --- | --- | --- |
| [38] | Hart, 1988 | diarrhea score ≥ 12 in a 24h period *patients with diarrhea on any day % Diarrhea days per feeding days* | 19/35 (54.3) 66/287 (23) | 19/33 (57.6) 68/297 (22.9) |
| [39] | Dobb, 1990 | diarrhea score based on the frequency, volume and consistency of the stool; diarrhea = daily score > 12 | 16/45 (35.6) | 13/46 (28.2) |
| [47] | Celaya, 1992 | NR | 3/17 (17.6) | NR |
| [35] | Caparrós, 2000 | ≥ 5 liquid stools in a 24-hour period or an estimated volume ≥ 2000ml/d *number of episodes per 1000 days of enteral nutrition* | 5.8 | 50 |
| [41] | Schultz, 2000 | Hart and Dobb diarrhea scale, diarrhea was defined as 2 or more days of diarrhea scores ≥ 12 during study days 3 through 8 | 11/33 (33.3) [fiber + placebo 6/11 (54.5)] [fiber-free + pectin 4/11 (36.3)] [fiber + pectin 1/11 (9.09)] | 1/11 (9.09) |
| [43] | Spapen, 2001 | Hart and Dobb diarrhea scale, diarrhea was defined as a daily score ≥ 12 *Number of patients with at least 1 day with diarrhea Percentage of days with diarrhea per feeding days Diarrhea score* | 6/13 (46.2) 8.8 ± 10; 16/148 (10.8) 4.8 ± 6.4 | 11/12 (91.2) 32 ± 15.3; 46/146 (31.5) 9.4 ± 10.2 |
| [40] | Rushdi, 2004 | NR | NR | NR |
| [37] | Palmese, 2006 | NR | 0/42 | 5/42 (11.9) |
| [49] | Karakan, 2007 | NR | NR | NR |
| [46] | Spindler-Vesel, 2007 | NR | NR | NR |
| [45] | Chittawatanarat, 2010 | Hart and Dobb criteria: daily accumulation score ≥ 12 *number of patients with at least 1 day of diarrhea Accumulation diarrhea score  Mean diarrhea score Incidence rate of diarrhea (100 patient-fed day) Probability of first diarrhea beginning at day 14th (95% CI)* | 4/17 (23.5) 50.8 ± 32 3.6 ± 2.3 6.7 0.24 (0.1-0.5) | 8/17 (47.1) 87.7 ± 50.7 6.3 ± 3.6 14.8 0.47 (0.3-0.7) |
| [52] | Zavertailo, 2010 | NR | NR | NR |
| [53] | Aytünür, 2012 | ≥ 5 watery stools in 24 hours or ≥ 2000ml of watery stool | 0/30 (0) | 0/30 (0) |
| [51] | Wang, 2014 | NR | NR | NR |
| [36] | Kamarul Zaman, 2016 | Diarrhea is defined using the faecal score of the King's stool chart. A score of ≥ 15 was used to indicate diarrhea. *No of patients with at least one day of diarrhea* | 18/35 (55) | 18/33 (51) |
| [44] | Yagmurdur, 2016 | Hart and Dobb diarrhea scale, diarrhea was defined as a daily score ≥ 12 *Patients with at least 1 day with diarrhea Daily diarrhea score, Day 1 Daily diarrhea score, Day 2 Daily diarrhea score, Day 3 Daily diarrhea score, Day 4 Daily diarrhea score, Day 5 Diarrhea score for five days* | 22/60 (36.7) 7.1 ± 7.1 7.1 ± 5.6 7.6 ± 6.1 8.6 ± 6.6 7.3 ± 5.9 7.5 ± 4.7 | 38/60 (63.3) 8.5 ± 6.9 8.7 ± 7.5 10.2 ± 8 11.7 ± 7.8 12 ± 8.4 10.2 ± 5.4 |
| [54] | Xi, 2017 | NR | 7/62 (11.3) | 16/63 (25.4) |
| [50] | Fazilaty, 2018 | NR | NR | NR |
| [42] | Freedberg, 2020 | NR | NR | NR |
| [48] | Chen, 2021 | >3 loose or liquid stools per day or an estimated volume > 2000ml/d | 2/24 (8.33) | 9/22 (40.9) |

### Table S7: Adverse events and serious adverse events

| **Reference No** | **Author, year (Country)** | **Adverse events** | | | **Serious adverse events** | | |
| --- | --- | --- | --- | --- | --- | --- | --- |
|  |  | **Definition** | **Fiber** | **Control** | **Definition** | **Fiber** | **Control** |
| [38] | Hart, 1988 (Australia) | NR | - | - | NR | - | - |
| [39] | Dobb, 1990 (Australia) | NR | - | - | NR | - | - |
| [47] | Celaya, 1992 (Spain) | NR | - | - | NR | - | - |
| [35] | Caparros, 2000 (Spain) | NR | - | - | NR | - | - |
| [41] | Schultz, 2000 (USA) | NR | - | - | NR | - | - |
| [43] | Spapen, 2001 (Belgium) | NR | - | - | NR | - | - |
| [40] | Rushdi, 2004 (Egypt) | NR | - | - | NR | - | - |
| [37] | Palmese, 2006 (Italy) | NR | - | - | NR | - | - |
| [49] | Karakan, 2007 (Turkey) | NR | - | - | NR | - | - |
| [46] | Spindler-Vesel, 2007 (Slovenia) | NR | - | - | NR | - | - |
| [45] | Chittawatanarat, 2010 (Thailand) | NR | - | - | NR | - | - |
| [52] | Zavertailo, 2010 (Russia) | NR | - | - | NR |  |  |
| [53] | Aytünür, 2012 (Turkey) | NR | - | - | NR | - | - |
| [51] | Wang, 2014 (China) | gastrointestinal adverse reactions: nausea, vomiting, digestive bleeding, abdominal distension, diarrhea, constipation | 6/43 (13.95) | 13/43 (30.23) | NR |  |  |
| [36] | Kamarul Zaman, 2016 (Malaysia) | NR | - | - | NR | - | - |
| [44] | Yagmurdur, 2016 (Turkey) | NR | - | - | NR | - | - |
| [54] | Xi, 2017 (China) | NR | - | - | NR | - | - |
| [50] | Fazilaty, 2018 (Iran) | NR | - | - | NR | - | - |
| [42] | Freedberg, 2020 (USA) | ascertain untoward health events, graded in terms of severity and relatedness to the study intervention | mild: 10/25 (5 definitely unrelated, 5 unlikely related)  moderate: 8/25 (7 definitely unrelated, 1 unlikely related) | mild: 4/20 (2 definitely unrelated, 2 unlikely related) moderate: 7/20 (7 definitely unrelated) | ascertain untoward health events, graded in terms of severity and relatedness to the study intervention | severe: 5/25 (5 definitely unrelated) life threatening: 2/25 (2 definitely unrelated) | severe: 7/20 (7 definitely unrelated) life threatening: 3/20 (3 definitely unrelated) |
| [48] | Chen, 2021 (China) | No definition | 0/24 | - | NR | - | - |

### Table S8: Clinical Trial Registry of ongoing or unpublished studies

| **No** | **Trial Registration Number** | **Country** | **Title** | **Last update** | **Status** |
| --- | --- | --- | --- | --- | --- |
| 1 | ChiCTR-INR-17012709 | China | The effect of early enteral nutrition intervention on patients with severe infections after cardiac surgery to intestinal microflora and metabolize | October 2, 2017 | Recruiting |
| 2 | ChiCTR1900021972 | China | Multicenter clinical study on the effects of enteral nutrition with pectin dietary fiber solution in ICU patients. | March 25, 2019 | Not yet recruiting |
| 3 | NCT03153397 | USA | Effect of Prebiotic Fiber- Enriched (scFOS) Enteral Feeding on the Microbiome in Neurological Injury Trauma Patients (PreFEED Microbiome Trial) | June 4, 2021 | Completed, data not published yet |
| 4 | NCT04438473 | China | Pectin Supplemented Enteral Feedings in Critically Ill Patients | July 15, 2021 | Withdrawn |
| 5 | ChiCTR2300072211 | China | A clinical controlled study of pectin "semi-solidification" for improving tolerance to enteral nutrition | July 25, 2023 | Recruitment completed |

### Table S9: List of excluded studies after full-text review with reasons

| **No** | **Reason for exclusion** | **Reference** |
| --- | --- | --- |
| 1 | Wrong study population (Patients < 16 years of age included) | Olguin F, Araya M, Hirsch S, Brunser O, Ayala V, Rivera R, Gotteland M. Prebiotic ingestion does not improve gastrointestinal barrier function in burn patients. Burns. 2005 Jun;31(4):482-8. doi: 10.1016/j.burns.2004.11.017 |
| 2 | Wrong intervention/control group (enteral vs. combined vs. parenteral nutrition) | Elke G, Kuhnt E, Ragaller M, Schadler D, Frerichs I, Brunkhorst FM, et al. Enteral nutrition is associated with improved outcome in patients with severe sepsis: a secondary analysis of the VISEP trial. Med Klin Intensivmed Notfallmed. (2013) 108:223–33. doi: 10.1007/s00063-013-0224-4 |
| 3 | Wrong intervention/control group (enteral vs. combined vs. parenteral nutrition) | Fan M-C,Qiao-ling W, Wei F, Yun-xia J, Lian-di L, Sun P, et al. Early enteral combined with parenteral nutrition treatment for severe traumatic brain injury: effects on immune function, nutritional status and outcomes. Chin MedSci J. (2016) 31:213–20. doi: 10.1016/S1001-9294(17)30003-2 |
| 4 | Wrong intervention and control group (many combined interventions vs. Total parenteral nutrition) | Sun B, Gao Y, Xu J, Zhou XL, Zhou ZQ, Liu C, et al. Role of individually staged nutritional support in the management of severe acute pancreatitis. Hepatobil Pancreat Dis Int. (2004) 3:458–63 |
| 5 | Wrong intervention (synbiotics) | López de Toro Martín-Consuegra I, et al. Influencia de los simbióticos en la disfunción multiorgánica: ensayo aleatorizado y controlado. Med Clin (Barc). 2014. http://dx.doi.org/10.1016/j.medcli.2013.09.046 |
| 6 | Wrong intervention (synbiotic) | Jain PK, McNaught CE, Anderson AD, MacFie J, Mitchell CJ. Influence of synbiotic containing Lactobacillus acidophilus La5, Bifidobacterium lactis Bb 12, Streptococcus thermophilus, Lactobacillus bulgaricus and oligofructose on gut barrier function and sepsis in critically ill patients: a randomised controlled trial. Clin Nutr 2004;23:467–75 |
| 7 | Wrong intervention (synbiotic) | Kanazawa H, Nagino M, Kamiya S, et al. Synbiotics reduce postoperative infectious complications: a randomized con trolled trial in biliary cancer patients undergoing hepatectomy. Langenbecks Arch Surg 2005;390:104–13 |
| 8 | Wrong intervention (synbiotic) | Rayes N, Hansen S, Seehofer D, et al. Early enteral supply of f iber and Lactobacilli versus conventional nutrition: a con trolled trial in patients with major abdominal surgery. Nutrition 2002;18:609–15 |
| 9 | Wrong intervention (synbiotic) | Rayes N, Seehofer D, Theruvath T, et al. Supply of pre- and probiotics reduces bacterial infection rates after liver trans plantation—a randomized, double-blind trial. Am J Transplant 1995;5:125–30 |
| 10 | Wrong intervention (synbiotic) | Kotzampassi K, Giamarellos-Bourboulis EJ, Voudouris A, Kazamias P, Eleftheriadis E. Benefits of a synbiotic formula (Synbiotic 2000Forte) in critically ill trauma patients: earlyresults of a randomized controlled trial. World J Surg. (2006) 30:1848–55. doi: 10.1007/s00268-005-0653-1 |
| 11 | Wrong intervention (synbiotic) | Sramek V, Dadak L, Stouracova M, Stetka P, Kyr M, Ticha A, et al. Impact of addition of synbiotics (Synbiotic (2000). Forte) to enteral nutrition on the course of MODS, occurrence of sepsis, immune status and gut function in long-term critically ill patients. Anesteziol Intenzivni Med. (2007) 18:157–63 |
| 12 | Wrong intervention (synbiotic) | Giamarellos-Bourboulis EJ, Bengmark S, Kanellakopoulou K, Kotzampassi K. Pro- and synbiotics to control inflammation and infection in patients with multiple injuries. J Trauma. (2009) 67:815–21. doi: 10.1097/TA.0b013e31819d979e |
| 13 | Wrong intervention (synbiotic) | Hayakawa M, Asahara T, Ishitani T, Okamura A, Nomoto K, Gando S. Synbiotic therapy reduces the pathological gram-negative rods caused by an increased acetic acid concentration in the gut. Digest Dis Sci. (2012) 57:2642–9. doi: 10.1007/s10620-012-2201-9 |
| 14 | Wrong intervention (probiotic), Abstract only | Gomersall CD, Joynt GM, Leung P, Tan P, Bengmark S. Does routine administration of probiotics improve outcome of critically ill patients? ANZCA ASM 2006. |
| 15 | Wrong intervention (probiotic), Abstract only | Malian M, Reichenbach R, Peck A, Pamukov N. Probiotic supplementation in critical care. Crit Care Med. (2012) 40:1–328. doi: 10.1097/01.ccm.0000425300.89190.4b |
| 16 | Wrong intervention (Probiotic) | Bleichner G, Blehaut H, Mentec H, Moyse D. Saccharomyces boulardii prevents diarrhea in critically ill tube-fed patients. Intensive care medicine. 1997;23(5):517–23. https://doi.org/10.1007/s001340050367 |
| 17 | Wrong intervention (Probiotic) | Frohmader TJ, Chaboyer WP, Robertson IK, Gowardman J. Decrease in frequency of liquid stool in enterally fed critically ill patients given the multispecies probiotic VSL#3: a pilot trial. Am J Crit Care. 2010;19(3):e1–11. https://doi.org/10.4037/ajcc2010976 |
| 18 | Wrong intervention (Probiotic) | Barraud D, Blard C, Hein F, Marcon O, Cravoisy A, Nace L, et al. Probiotics in the critically ill patient: a double blind, randomized, placebo-controlled trial. Intensive Care Med. 2010;36(9):1540–7. https://doi.org/10.1007/s00134-010-1 927-0 |
| 19 | Wrong intervention (Probiotic) | Morrow LE, Kollef MH, Casale TB. Probiotic prophylaxis of ventilator associated pneumonia: a blinded, randomized, controlled trial. Am J Respir Crit Care Med. 2010;182(8):1058–64. https://doi.org/10.1164/rccm.200912-1 853OC |
| 20 | Wrong intervention (Probiotic) | Sanaie S, Ebrahimi-Mameghani M, Hamishehkar H, Mojtahedzadeh M, Mahmoodpoor A. Effect of a multispecies Probiotic on inflammatory markers in critically ill patients: a randomized, double-blind, placebo controlled trial. J Res Med Sci. 2014;19(9):827–33 |
| 21 | Wrong intervention (Probiotic) | Malik AA, Rajandram R, Tah PC, Hakumat-Rai VR, Chin KF. Microbial cell preparation in enteral feeding in critically ill patients: A randomized, double blind, placebo-controlled clinical trial. J Crit Care. 2016;32:182–8. https://doi. org/10.1016/j.jcrc.2015.12.008 |
| 22 | Wrong intervention (Probiotic) | Shimizu K, Yamada T, Ogura H, Mohri T, Kiguchi T, Fujimi S, Asahara T, Yamada T, Ojima M, Ikeda M, Shimazu T. Synbiotics modulate gut microbiota and reduce enteritis and ventilator-associated pneumonia in patients with sepsis: a randomized controlled trial. Crit Care. 2018;22(1):239. https://doi.org/10.1186/s13054-018-2167-x |
| 23 | Wrong intervention (probiotic) | Li, Y. M. (2007). Adjuvant therapy for probiotics in patients with severe acute pancreatitis: an analysis of 14 cases. World Chin. J. Digestol. 15, 302–304. doi: 10.3969/j.issn.1009-3079.2007.03.019 |
| 24 | Wrong intervention (probiotic) | Besselink, M. G., van Santvoort, H. C., Buskens, E., Boermeester, M. A., van Goor, H., Timmerman, H. M., et al. (2008). Probiotic prophylaxis in predicted severe acute pancreatitis: a randomised, double-blind, placebo-controlled trial. Lancet 371, 651–659. doi: 10.1016/S0140-6736(08)60207-X |
| 25 | Wrong intervention (probiotic) | Wu, X. G., and Zhang, Q. C. (2009). Adjuvant therapy for probiotics in patients with severe acute pancreatitis with hepatic lesion: an analysis of 27 cases. Clin. Med. 29, 51–52 |
| 26 | Wrong intervention (probiotic) | Lata, J., Jurankova, J., Stiburek, O., Pribramska, V., Senkyrik, M., and Vanasek, T. (2010). Probiotics in acute pancreatitis- A randomised, placebo-controlled, double-blind study. Vnitr. Lek. 56, 111–114 |
| 27 | Wrong intervention (probiotic) | Cui, L.-H., Wang, X.-H., Peng, L.-H., Yu, L., and Yang, Y.-S. (2013). The effects of early enteral nutrition with addition of probiotics on the prognosis of patients suffering from severe acute pancreatitis, Chinese critical care medicine. Zhonghua Wei Zhong Bing Ji Jiu Yi Xue. 25, 224–228. doi: 10.3760/cma.j.issn.2095-4352.2013.04.011 |
| 28 | Wrong intervention (probiotic) | Wang, G., Wen, J., Xu, L., Zhou, S., Gong, M., Wen, P., et al. (2013). Effect of enteral nutrition and ecoimmunonutrition on bacterial translocation and cytokine production in patients with severe acute pancreatitis. J. Surg. Res. 183, 592–597. doi: 10.1016/j.jss.2012.12.010 |
| 29 | Wrong intervention (probiotic) | Zhu, Y. M., Lin, S., Dang, X. W., Wang, M., Li, L., Sun, R. Q., et al. (2014). Effects of probiotics in treatment of severe acute pancreatitis. World Chin. J. Digestol. 22, 5013–5017. doi: 10.11569/wcjd.v22.i32.5013 |
| 30 | Wrong intervention (probiotic) | Li, J., Wang, J., and Xu, Y. Q. (2014). Effect of early enteral nutrition with Bifico on levels of inflammatory mediators in plasma of patients with severe acute pancreatitis. World Chin. J. Digestol. 22, 5609–5614. doi: 10.11569/wcjd.v22.i36.5609 |
| 31 | Wrong intervention (probiotic) | Wu,P., Yu, Y., Li, L., and Sun, W. (2017). Effect and safety of probiotics combined early enteral nutrition on severe acute pancreatitis patients. Biomed. Res. 28, 1403–1407 |
| 32 | Wrong intervention (probiotic) | Klarin B, Johansson ML, Molin G, Larsson A, Jeppsson B. Adhesion of the probiotic bacterium Lactobacillus plantarum 299v onto the gut mucosa in critically ill patients: a randomised open trial. Crit Care 2005;9:R285–93 |
| 33 | Wrong intervention (probiotic) | McNaught CE, Woodcock NP, Anderson AD, MacFie J. A prospective randomised trial of probiotics in critically ill patients. Clin Nutr 2005;24:211–9 |
| 34 | Wrong intervention (probiotic) | Morrow LE, Kollef MH, Bowers JB, Casale TB. Probiotic manipulation of the native flora in critically ill patients: an opportunity for ventilator-associated pneumonia prophylaxis? Chest. (2005) 128:144S. doi: 10.1378/chest.128.4_MeetingAbstracts.144S |
| 35 | Wrong intervention (probiotic) | Alberda C, Gramlich L, Meddings J, Field C, McCargar L, Kutsogiannis D, et al. Effects of probiotic therapy in critically ill patients: a randomized, double-blind, placebo-controlled trial. Am J Clin Nutr. (2007) 85:816–23. doi: 10.1093/ajcn/85.3.816 |
| 36 | Wrong intervention (probiotic) | Forestier C, Guelon D, Cluytens V, Gillart T, Sirot J, De Champs C. Oral probiotic and prevention of Pseudomonas aeruginosa infections: a randomized, double-blind, placebo-controlled pilot study in intensive care unit patients. Critical care. (2008) 12:R69. doi: 10.1186/cc6907 |
| 37 | Wrong intervention (probiotic) | Klarin B, Wullt M, Palmquist I, Molin G, Larsson A, Jeppsson B. Lactobacillus plantarum 299v reduces colonisation of Clostridium difficile in critically ill patients treated with antibiotics. Acta Anaesthesiol Scand. (2008) 52:1096–102. doi: 10.1111/j.1399-6576.2008.01748.x |
| 38 | Wrong intervention (probiotic) | Tan M, Zhu JC, Du J, Zhang LM, Yin HH. Effects of probiotics on serum levels of Th1/Th2 cytokine and clinical outcomes in severe traumatic brain injured patients: a prospective randomized pilot study. Crit Care. (2011) 15:R290. doi: 10.1186/cc10579 |
| 39 | Wrong intervention (probiotic) | Tan M, Xiao-lan L, Jun-wei D, Hua P, Jing-ci Z. Effects of probiotics on blood glucose levels and clinical outcomes in patients witn severe craniocerebral trauma. Chin Crit Care Med. (2013) 25:627–30. doi: 10.3760/cma.j.issn.2095-4352.2013.10.012 |
| 40 | Wrong intervention (probiotic) | Rongrungruang Y, Krajangwittaya D, Pholtawornkulchai K, Tiengrim S, Thamlikitkul V. Randomized controlled study of probiotics containing Lactobacillus casei (Shirota strain) for prevention of ventilator-associated pneumonia. J Med Assoc Thai. (2015) 98:253–9 |
| 41 | Wrong intervention (probiotic) | Zarinfar N, Sharafkhah M, Amiri M, Rafeie M. Probiotic effects in prevention from ventilator-associated pneumonia. Koomesh. (2016) 7:803–13 |
| 42 | Wrong intervention (probiotic) | Zeng J, Wang CT, Zhang FS, Qi F, Wang SF, Ma S, et al. Effect of probiotics on the incidence of ventilator-associated pneumonia in critically ill patients: a randomized controlled multicenter trial. Intens Care Med. (2016) 42:1018 28. doi: 10.1007/s00134-016-4303-x |
| 43 | Wrong intervention (probiotic) | Alberda C, Marcushamer S, Hewer T, Journault N, Kutsogiannis D. Feasibility of a Lactobacillus casei drink in the intensive care unit for prevention of antibiotic associated diarrhea and Clostridium difficile. Nutrients. (2018) 10:539. doi: 10.3390/nu10050539 |
| 44 | Wrong intervention (probiotic) | Mahmoodpoor A, Hamishehkar H, Asghari R, Abri R, Shadvar K, Sanaie S. Effect of a probiotic preparation on ventilator-associated pneumonia in critically ill patients admitted to the intensive care unit: a prospective double-blind randomized controlled trial. Nutr Clin Pract. (2019) 34:156–62. doi: 10.1002/ncp.10191 |
| 45 | Wrong intervention (probiotic) | Li YM. Adjuvant therapy for probiotics in patients with se vere acute pancreatitis: An analysis of 14 cases. Shijie Huaren Xiaohua Zazhi 2007; 15: 302-304 |
| 46 | Wrong intervention (probiotic) | Wu XG, Zhang QC. Adjuvant therapy for probiotics in pa tients with severe acute pancreatitis with hepatic lesion: an analysis of 27 cases. Clin Med 2009; 29: 51-52 |
| 47 | Wrong intervention (many combined interventions but no fiber) | Kudsk KA, Minard G, Croce MA, Brown RO, Lowrey TS, Pritchard FE, et al. A randomized trial of isonitrogenous enteral diets after severe trauma: an immune-enhancing diet reduces septic complications. Ann Surg. (1996) 224:531–43. doi: 10.1097/00000658-199610000-00011 |
| 48 | Wrong intervention (glutamine + probiotics) | Falcão De Arruda IS, De Aguilar-Nascimento JE. Benefits of early enteral nutrition with glutamine and probiotics in brain injury patients. Clin Sci. (2004) 106:287–92. doi: 10.1042/CS20030251 |
| 49 | Wrong control group (fiber-containing formula vs. fiber-containing formula) | van Steen SC, Rijkenberg S, Sechterberger MK, DeVries JH, van der Voort PHJ. Glycemic Effects of a Low-Carbohydrate Enteral Formula Compared With an Enteral Formula of Standard Composition in Critically Ill Patients: An Open-Label Randomized Controlled Clinical Trial. JPEN J Parenter Enteral Nutr. 2018 Aug;42(6):1035-1045. doi: 10.1002/jpen.1045 |
| 50 | Wrong control group (fiber-containing formula + fiber vs. fiber-containing formula + placebo) | Majid HA, Cole J, Emery PW, Whelan K. Additional oligofructose/inulin does not increase faecal bifidobacteria in critically ill patients receiving enteral nutrition: a randomised controlled trial. Clin Nutr. 2014 Dec;33(6):966-72. doi: 10.1016/j.clnu.2013.11.008 |
| 51 | Wrong control group (enteral vs. parenteral nutrition) | Petrov MS, Kukosh MV, Emelyanov NV. A randomized controlled trial of enteral versus parenteral feeding in patients with predicted severe acute pancreatitis shows a significant reduction in mortality and in infected pancreatic complications with total enteral nutrition. Digesti Surg. (2006) 23:336–44; discussion 344–335. doi: 10.1159/000097949 |
| 52 | Wrong control group (enteral vs. parenteral nutrition) | Abdulmeguid AM, Hassan A. Enteral versus parenteral nutrition in mechanically ventilated patients. Neurol Croatica. (2007) 56:15–24 |
| 53 | Wrong control group (enteral vs. parenteral nutrition) | Casas M, Mora J, Fort E, Farré A, Aracil C, Busquets D, et al. Total enteral nutrition vs. total parenteral nutrition in patients with severe acute pancreatitis. Rev Esp Enferm Dig. (2007) 99:264–9. doi: 10.4321/S1130-01082007000500004 |
| 54 | Wrong control group (enteral vs. parenteral nutrition) | Doley RP, Wig TDYJ, Kochhar R, Singh G, Bharathy KGS, Kudari A, et al. Enteral nutrition in severe acute pancreatitis. J Pancreas. (2009) 10:157–62. doi: 10.1115/1.1456090 |
| 55 | Wrong control group (enteral vs. parenteral nutrition) | Moses V, Mahendri NV, John G, Peter JV, Ganesh A. Early hypocaloric enteral nutritional supplementation in acute organophosphate poisoning a prospective randomized trial. Clin Toxicol. (2009) 47:419–24. doi: 10.1080/15563650902936664 |
| 56 | Wrong control group (enteral vs. parenteral nutrition) | Fu Y-H, Jian-Bo W, Gui-Liang W, Ping W, Min G, Ming H, et al. Effect of enteral nutrition on cytokine production and plasma endotoxin in patients with severe acute pancreatitis. World Chin J Digestol. (2015) 23:1174. doi: 10.11569/wcjd.v23.i7.1174 |
| 57 | Wrong control group (enteral vs. parenteral nutrition) | Kim JM, Joh JW, Kim HJ, Kim SH, Rha M, Sinn DH, et al. Early enteral feeding after living donor liver transplantation prevents infectious complications: a prospective pilot study. Medicine. (2015) 94:e1771. doi: 10.1097/MD.0000000000001771 |
| 58 | Wrong control group (enteral vs. parenteral nutrition) | Reignier J, Boisramé-Helms J, Brisard L, Lascarrou J-B, Ait Hssain A, Anguel N, et al. Enteral versus parenteral early nutrition in ventilated adults with shock: a randomised, controlled, multicentre, open label, parallel-group study (NUTRIREA-2). Lancet. (2018) 391:133–43. doi: 10.1016/S01406736(17)32146-3 |
| 59 | Wrong control group (critically ill patients vs. healthy individuals) | Hong-Guang Lu, Yu-Be Shi, Li-Ming Zhao, Chunxue Bai & Xiangdong Wang (2008) Role of enteral ebselen and ethylhydroxyethyl cellulose in pancreatitis-associated multiple-organ dysfunction in humans, Journal of Organ Dysfunction, 4:1, 43-50, DOI: 10.1080/17471060701486209 |
| 60 | Systematic review, Not critically ill patients | Gurusamy KS, Nagendran M, Davidson BR. Methods of preventing bacterial sepsis and wound complications a#er liver transplantation. Cochrane Database of Systematic Reviews 2014, Issue 3. Art. No.: CD006660. DOI: 10.1002/14651858.CD006660.pub3 |
| 61 | Systematic review, Not critically ill patients | Poropat G, Giljaca V, Hauser G, Štimac D. Enteral nutrition formulations for acute pancreatitis. Cochrane Database of Systematic Reviews 2015, Issue 3. Art. No.: CD010605. DOI: 10.1002/14651858.CD010605.pub2 |
| 62 | Systematic review - included studies are reviewed | Hajipour A, Afsharfar M, Jonoush M, et al. The effects of dietary fiber on commoncomplications in critically ill patients; with a special focus on viral infections; a systematic reveiw. Immun Inflamm Dis. 2022;10:e613. doi:10.1002/iid3.613 |
| 63 | Systematic review - included studies are reviewed | Li C, Liu L, Gao Z, Zhang J, Chen H, Ma S, Liu A, Mo M, Wu C, Chen D, Liu S, Xie J, Huang Y, Qiu H and Yang Y (2021) Synbiotic Therapy Prevents Nosocomial Infection in Critically Ill Adult Patients: A Systematic Review and Network Meta-Analysis of Randomized Controlled Trials Based on a Bayesian Framework. Front. Med. 8:693188. doi: 10.3389/fmed.2021.693188 |
| 64 | Systematic review - included studies are reviewed | Seifi N, Jafarzadeh Esfahani A, Sedaghat A, Rezvani R, Khadem-Rezaiyan M, Nematy M, Safarian M. Effect of gut microbiota modulation on feeding tolerance of enterally fed critically ill adult patients: a systematic review. Syst Rev. 2021 Apr 2;10(1):95. doi: 10.1186/s13643-021-01633-5 |
| 65 | Systematic review - included studies are reviewed | Tian X, Pi Y-P, Liu X-L, Chen H and Chen W-Q (2018) Supplemented Use of Pre-, Pro-, and Synbiotics in Severe Acute Pancreatitis: An Updated Systematic Review and Meta-Analysis of 13 Randomized Controlled Trials. Front. Pharmacol. 9:690. doi: 10.3389/fphar.2018.00690 |
| 66 | Systematic review - included studies are reviewed | Watkinson PJ, Barber VS, Dark P, Young JD. The use of pre- pro- and synbiotics in adult intensive care unit patients: systematic review. Clin Nutr. 2007 Apr;26(2):182-92. doi: 10.1016/j.clnu.2006.07.010 |
| 67 | Systematic review - included studies are reviewed | Yang G, Wu XT, Zhou Y, Wang YL. Application of dietary fiber in clinical enteral nutrition: A meta-analysis of randomized controlled trials. World J Gastroenterol 2005; 11(25): 3935-3938 |
| 68 | Systematic review - included studies are reviewed | Zhang MM, Cheng JQ, Lu YR, Yi ZH, Yang P, Wu XT. Use of pre-, pro- and synbiotics in patients with acute pancreatitis: A meta-analysis. World J Gastroenterol 2010; 16(31): 3970-3978 |
| 70 | Not RCT, not critically ill | Buil‐Cosiales P, Zazpe I, Toledo E, et al. Fiber intake and all cause mortality in the Prevención con Dieta Mediterránea (PREDIMED) study. Am J Clin Nutr. 2014;100(6):1498‐1507 |
| 71 | Not RCT, not critically ill | Berthon BS, Macdonald‐Wicks LK, Gibson PG, Wood LG. Investigation of the association between dietary intake, disease severity and airway inflammation in asthma. Respirology. 2013;18(3):447‐454 |
| 72 | Not RCT, not critically ill | Halnes I, Baines KJ, Berthon BS, MacDonald‐Wicks LK, Gibson PG, Wood LG. Soluble fibre meal challenge reduces airway inflammation and expression of GPR43 and GPR41 in asthma. Nutrients. 2017;9(1):57 |
| 73 | Not RCT, not critically ill | Katagiri R, Goto A, Sawada N, et al. Dietary fiber intake and total and cause‐specific mortality: the Japan Public Health Center‐based prospective study. Am J Clin Nutr. 2020;111(5): 1027‐1035 |
| 74 | not RCT, not critically ill | Park Y, Subar AF, Hollenbeck A, Schatzkin A. Dietary fiber intake and mortality in the NIH‐AARP diet and health study. Arch Intern Med. 2011;171(12):1061‐1068 |
| 75 | Not RCT, not critically ill | Salmean YA, Segal MS, Langkamp‐Henken B, Canales MT, Zello GA, Dahl WJ. Foods with added fiber lower serum creatinine levels in patients with chronic kidney disease. J Ren Nutr. 2013;23(2):e29‐e32 |
| 76 | Not RCT (pseudorandomized) | Tuncay P, Arpaci F, Doganay M, Erdem D, Sahna A, Ergun H, Atabey D. Use of standard enteral formula versus enteric formula with prebiotic content in nutrition therapy: A randomized controlled study among neuro-critical care patients. Clin Nutr ESPEN. 2018 Jun;25:26-36. doi: 10.1016/j.clnesp.2018.03.123 |
| 77 | Not RCT | Chittawatanarat K, Surawang S, Simapaisan  P, Judprasong K. Jerusalem Artichoke Powder Mixed in Enteral Feeding for Patients Who have Diarrhea in Surgical Intensive Care Unit: A Method of Preparation and a Pilot Study. Indian J Crit Care Med 2020;24(11):1051–1056 |
| 78 | Not RCT | FuY,Moscoso DI, Porter J, et al. Relationship between dietary fiber intake and short‐chain fatty acid‐producing bacteria during critical illness: a prospective cohort study. JPEN J Parenter Enteral Nutr. 2020;44(3):463‐471 |
| 79 | Not RCT | Kooshki AZK, Zarghi A, Rad M, Tabaraie Y. Prebiotic prophylaxis of ventilator-associated pneumonia: a randomized clinical trial. Biomed Res Ther. (2018) 5:2287–95. doi: 10.15419/bmrat.v5i5.442 |
| 80 | Not critically ill, wrong intervention and control (probiotic vs. Fiber-containing) | Oláh A, Belágyi T, Issekutz A, Gamal ME, Bengmark S. Randomized clinical trial of specific lactobacillus and fibre supplement to early enteral nutrition in patients with acute pancreatitis. Br J Surg 2002; 89: 1103-1107 |
| 81 | Not critically ill patients | Plaudis, H., Pupelis, G., Zeiza, K., and Boka, V. (2012). Early low volume oral synbiotic/prebiotic supplemented enteral stimulation of the gut in patients with severe acute pancreatitis: a prospective feasibility study. Acta Chir. Belg. 112, 131–138. doi: 10.1080/00015458.2012.11680811 |
| 82 | Not critically ill patients | Qin HL, Zheng JJ, Tong DN, Chen WX, Fan XB, Hang XM, Jiang YQ. Effect of Lactobacillus plantarum enteral feed ing on the gut permeability and septic complications in the patients with acute pancreatitis. Eur J Clin Nutr 2008; 62: 923-930 |
| 83 | Not critically ill (both ICU and general medical care units) | Belknap D, Davidson LJ, Smith CR (1997). The effects of psyllium hydrophilic mucilloid on diarrhea in enterally fed patients. Heart & Lung, 26(3), 229–237. DOI: 10.1016/s0147-9563(97)90060-1 |
| 84 | Not critically ill | Chen C, Zeng Y, Xu J, et al. Therapeutic effects of soluble dietary fiber consumption on type 2 diabetes mellitus. Exp Ther Med. 2016;12(2):1232‐1242 |
| 85 | Not critically ill | Yasukawa Z, Inoue R, Ozeki M, Okubo T, Takagi T, Honda A, Naito Y. Effect of Repeated Consumption of Partially Hydrolyzed Guar Gum on Fecal Characteristics and Gut Microbiota: A Randomized, Double-Blind, Placebo-Controlled, and Parallel-Group Clinical Trial. Nutrients. 2019 Sep 10;11(9):2170. doi: 10.3390/nu11092170 |
| 86 | not critically ill | McLoughlin R, Berthon BS, Rogers GB, et al. Soluble fibre supplementation with and without a probiotic in adults with asthma: a 7‐day randomised, double blind, three way cross over trial. EBioMedicine. 2019;46:473‐485 |
| 87 | Not critically ill | Jakobsen LH, Wirth R, Smoliner C, Klebach M, Hofman Z, Kondrup J. Gastrointestinal tolerance and plasma status of carotenoids, EPA and DHA with a fiber‐enriched tube feed in hospitalized patients initiated on tube nutrition: randomized controlled trial. Clin Nutr. 2017;36(2):380‐388 |
| 88 | Not critically ill | Homann HH, Kemen M, Fuessenich C, Senkal M, Zumtobel V. Reduction in diarrhea incidence by soluble fiber in patients receiving total or supplemental enteral nutrition. J Parenter  Enteral Nutr 1994; 18: 486-490 |
| 89 | Not critically ill | Khalil L, Ho KH, Png D, Ong CL. The effect of enteral fibre containing feeds on stool parameters in the post-surgical period. Singapore Med J 1998; 39: 156-159 |
| 90 | No relevant outcome | Lee JG, Kim YS, Lee YJ, Ahn HY, Kim M, Kim M, et al. Effect of Immune-Enhancing Enteral Nutrition Enriched with or without Beta-Glucan on Immunomodulation in Critically Ill Patients. Nutrients. 2016;8(6) |
| 91 | Elective surgery patients, wrong intervention (many combined interventions but no fiber) | Braga M, Vignali A, Gianotti L, Cestari A, Profili M, Di Carlo V. Benefits of early postoperative enteral feeding in cancer patients. Infusionsther Transfusionsmed. (1995) 22:280–4. doi: 10.1159/000223143 |
| 92 | Elective surgery patients | Zhao R, WangY, HuangY, et al. Effects of fiber and probiotics on diarrhea associated with enteral nutrition in gastric cancer patients: a prospective randomized and controlled trial. Medicine (Baltimore). 2017;96(43):e8418 |

### Table S10: Canadian Critical Care Nutrition Methodological Scoring

| **Reference No** | **Author, year** | **Concealed Randomization** | **Intention-to-treat Analysis** | **Blinding** | **Patient Selection** | **Comparability of groups at baseline** | **Extent of Follow-up** | **Description of treatment protocol** | **Description of treatment co-interventions** | **Objectivity of the definition of outcomes** | | **Total score (max. 14)** | **Level*** |
| --- | --- | --- | --- | --- | --- | --- | --- | --- | --- | --- | --- | --- | --- |
| [38] | Hart, 1988 | 1 | 0 | 2 | 0 | 1 | 1 | 0 | 2 | 2 | | 9 | 2 |
| [39] | Dobb, 1990 | 1 | 0 | 1 | 0 | 1 | 1 | 0 | 0 | 2 | | 6 | 2 |
| [47] | Celaya, 1992 | 1 | 2 | 0 | 0 | 1 | 1 | 0 | 0 | 0 | | 5 | 2 |
| [35] | Caparrós, 2000 | 2 | 0 | 0 | 0 | 1 | 1 | 1 | 0 | 2 | | 7 | 2 |
| [41] | Schultz, 2000 | 1 | 0 | 2 | 0 | 0 | 1 | 0 | 0 | 2 | | 6 | 2 |
| [43] | Spapen, 2001 | 1 | 0 | 1 | 0 | 1 | 1 | 0 | 0 | 2 | | 6 | 2 |
| [40] | Rushdi, 2004 | 2 | 0 | 0 | 1 | 0 | 1 | 0 | 2 | 1 | | 7 | 2 |
| [37] | Palmese, 2006 | 2 | 2 | 0 | 0 | 1 | 1 | 0 | 0 | 0 | | 6 | 2 |
| [49] | Karakan, 2007 | 1 | 2 | 2 | 1 | 1 | 1 | 0 | 0 | 0 | | 8 | 2 |
| [46] | Spindler-Vesel, 2007 | 1 | 0 | 0 | 0 | 0 | 1 | 0 | 0 | 0 | | 2 | 2 |
| [45] | Chittawatanarat, 2010 | 1 | 2 | 1 | 0 | 1 | 1 | 0 | 1 | 1 | | 8 | 2 |
| [52] | Zavertailo, 2010 | 2 | 2 | 0 | 0 | 1 | 1 | 0 | 1 | 2 | | 9 | 2 |
| [53] | Aytünür, 2012 | 1 | 2 | 0 | 0 | 0 | 1 | 0 | 1 | 2 | | 7 | 2 |
| [51] | Wang, 2014 | 1 | 2 | 1 | 0 | 1 | 1 | 0 | 0 | 0 | | 6 | 2 |
| [36] | Kamarul Zaman, 2016 | 1 | 2 | 0 | 1 | 1 | 0 | 0 | 0 | 2 | | 7 | 2 |
| [44] | Yagmurdur, 2016 | 2 | 2 | 1 | 0 | 1 | 1 | 1 | 0 | 2 | | 10 | 2 |
| [54] | Xi, 2017 | 2 | 0 | 0 | 0 | 1 | 0 | 1 | 1 | 0 | | 5 | 2 |
| [50] | Fazilaty, 2018 | 2 | 0 | 2 | 0 | 0 | 1 | 0 | 0 | 1 | | 6 | 2 |
| [42] | Freedberg, 2020 | 1 | 0 | 0 | 0 | 1 | 1 | 0 | 1 | 1 | | 5 | 2 |
| [48] | Chen, 2021 | 2 | 0 | 1 | 0 | 0 | 1 | 0 | 0 | 2 | | 6 | 2 |
| **Median** | **6** |  |  |  |  |  |  |  |  |  |  |  |  |

* A trial was considered a level 1 study if all the following criteria were fulfilled: 1) concealed randomization, 2) double-blinded and 3) conduction of intention-to-treat-analysis. If any one of the above characteristics was unfulfilled, it was considered as a level 2 study.

### Figure S1: ROB2 traffic light and summary plots

#### 1a) Overall mortality

| 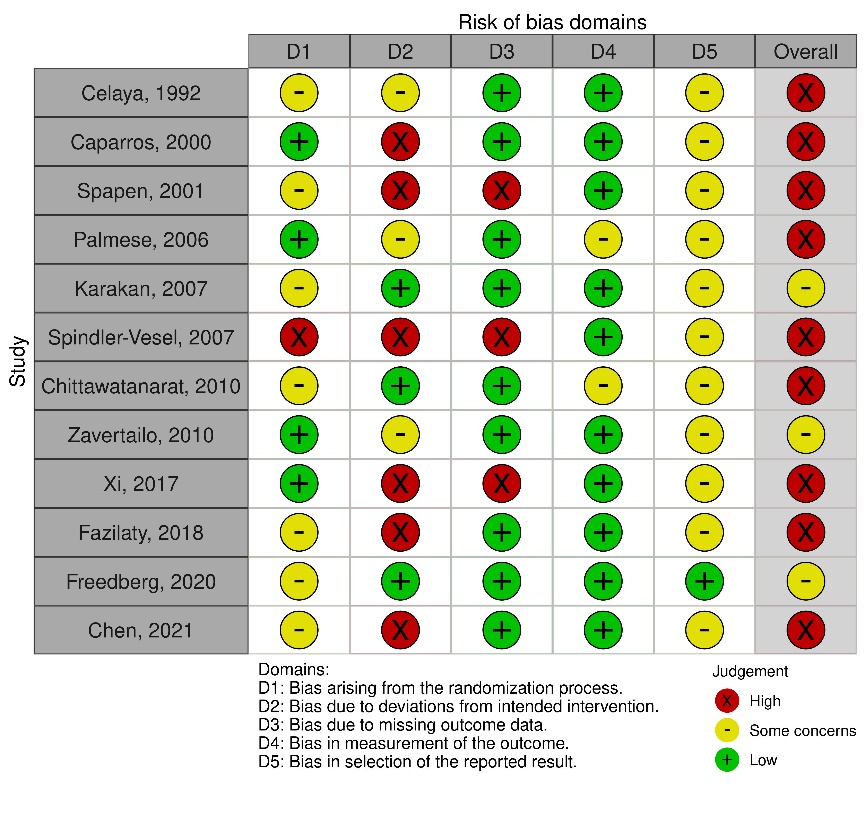 | 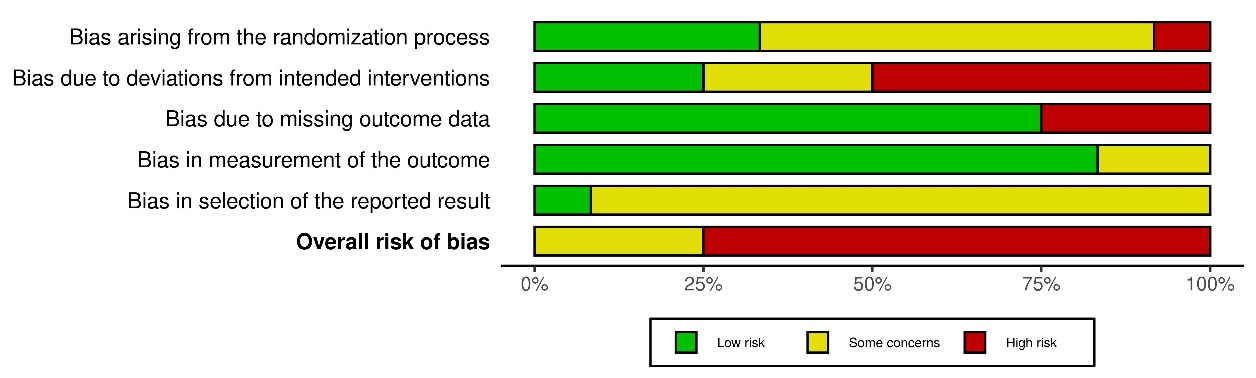 |
| --- | --- |

#### 1b) Diarrhea

| 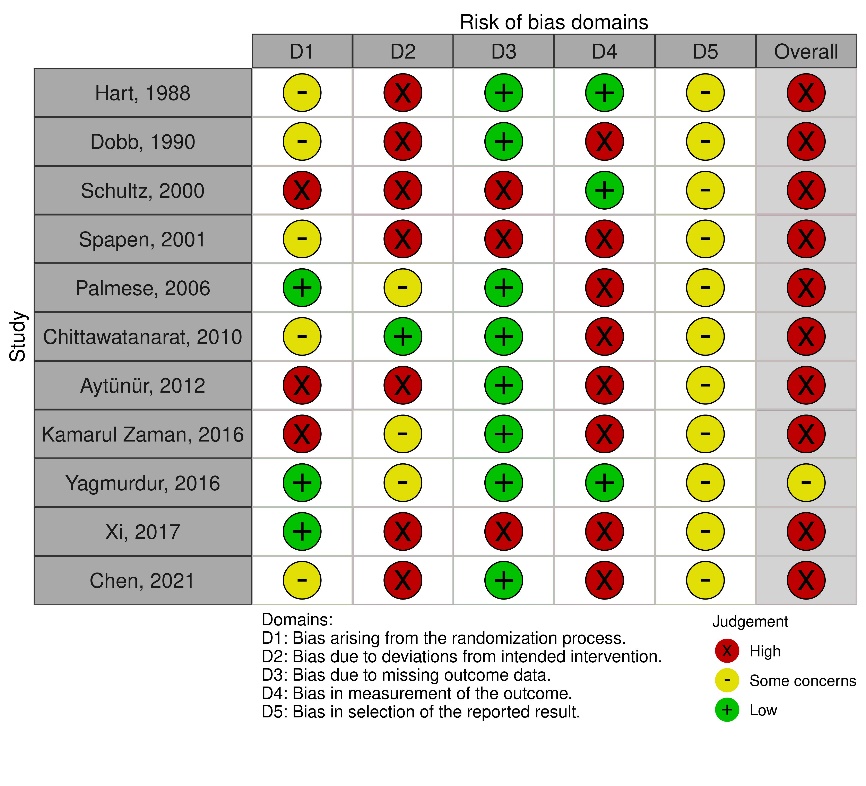 | 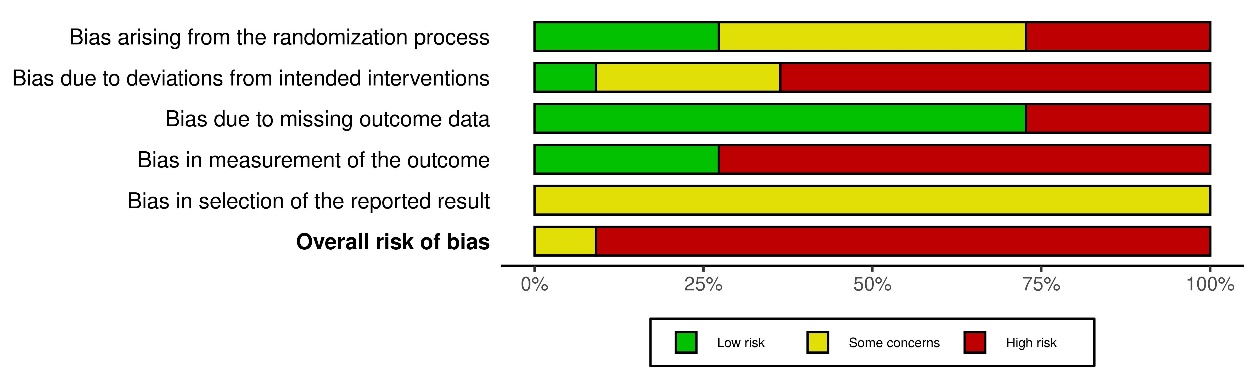 |
| --- | --- |

#### 1c) Other GI complications

| 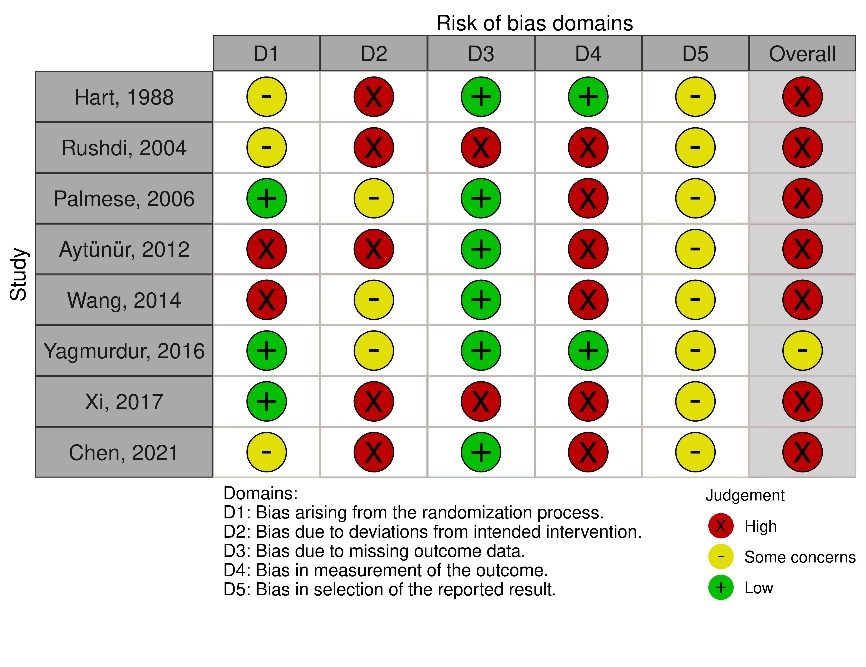 | 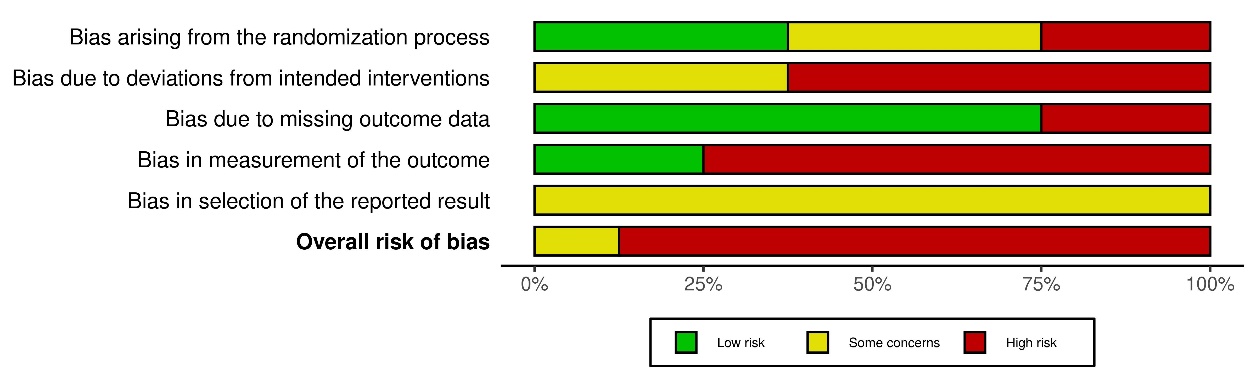 |
| --- | --- |

#### 1d) ICU LOS

| 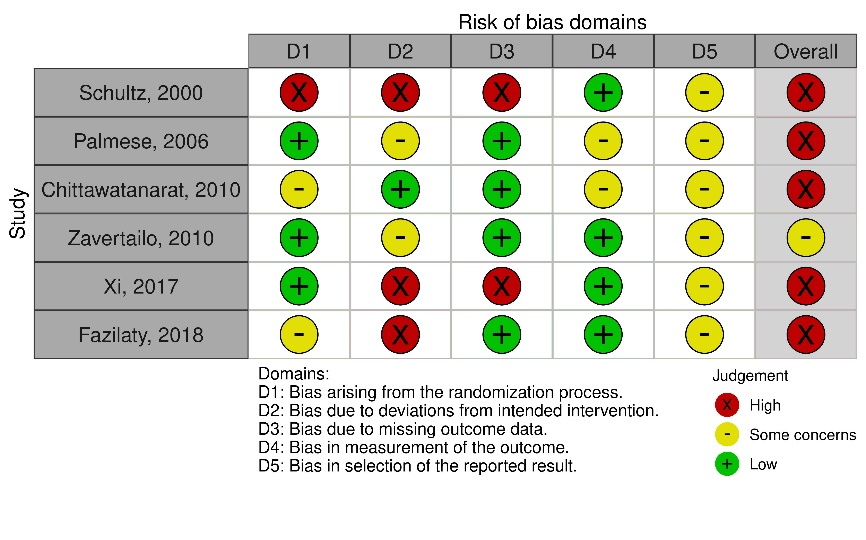 | 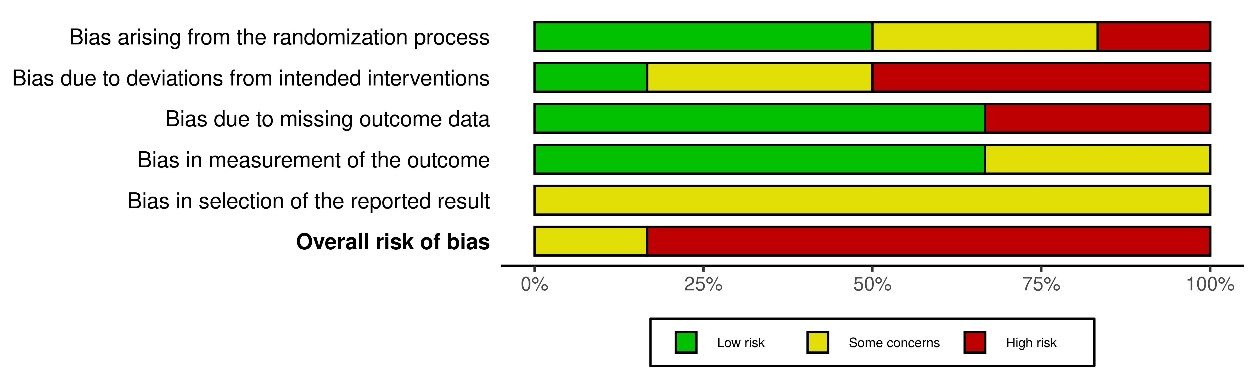 |
| --- | --- |

#### 1e) Hospital LOS

| 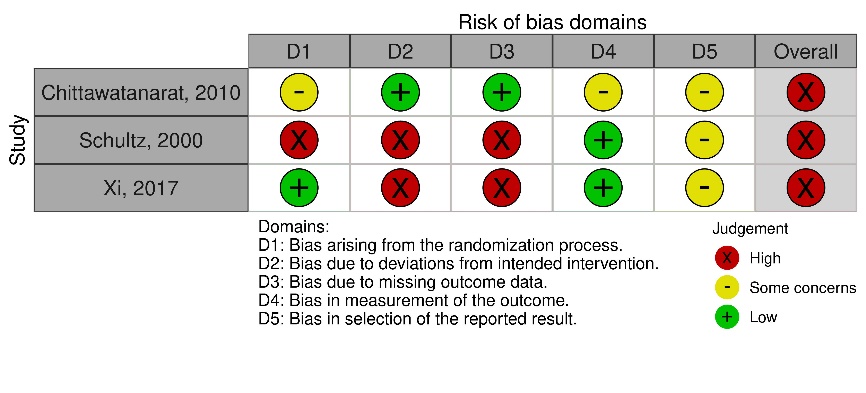 | 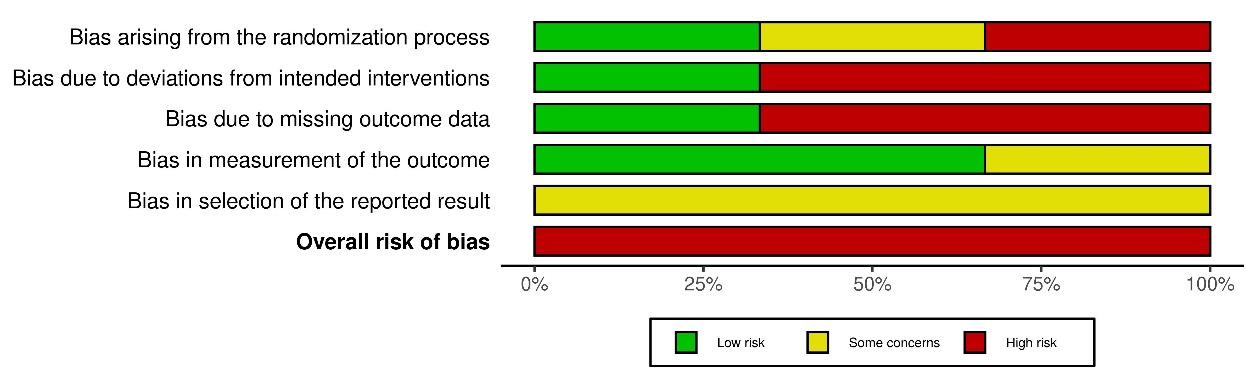 |
| --- | --- |

#### 1f) Infectious complications

| 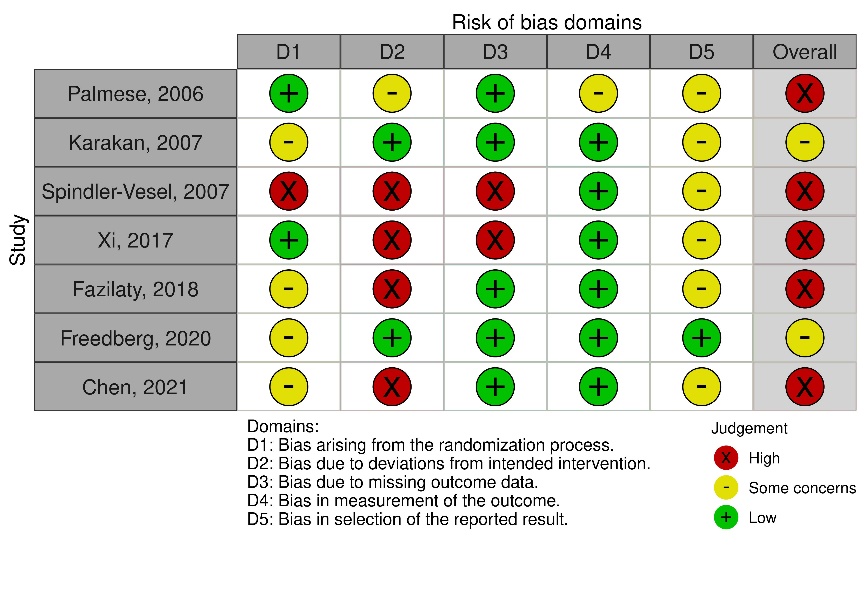 | 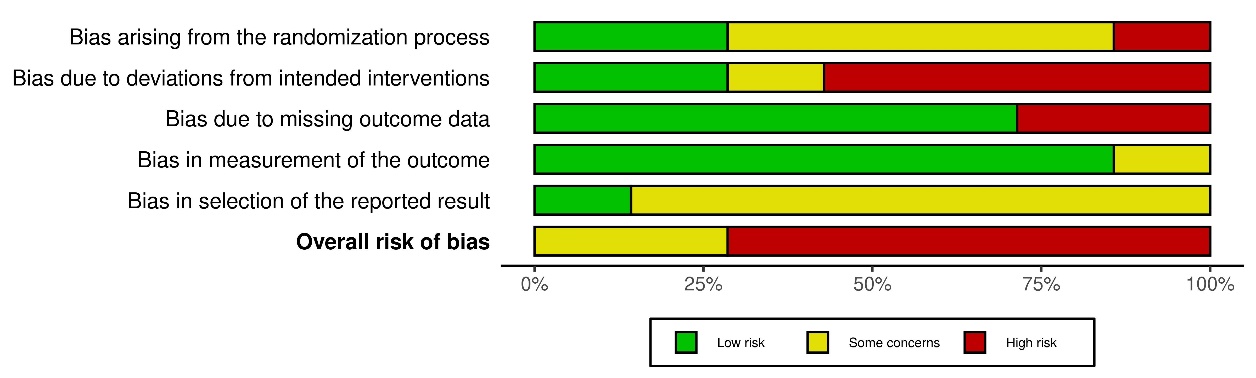 |
| --- | --- |

#### 1g) Duration of mechanical ventilation

| 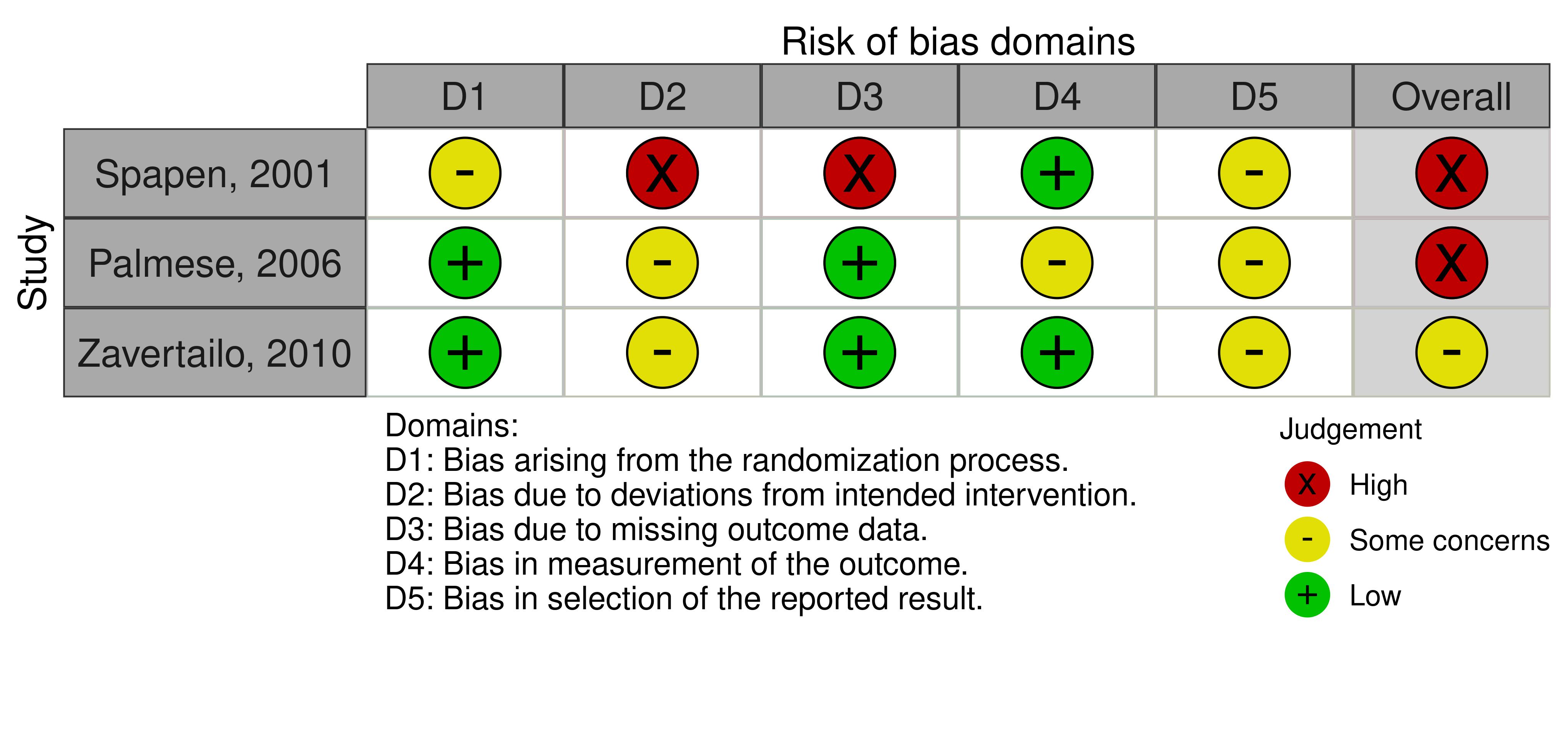 | 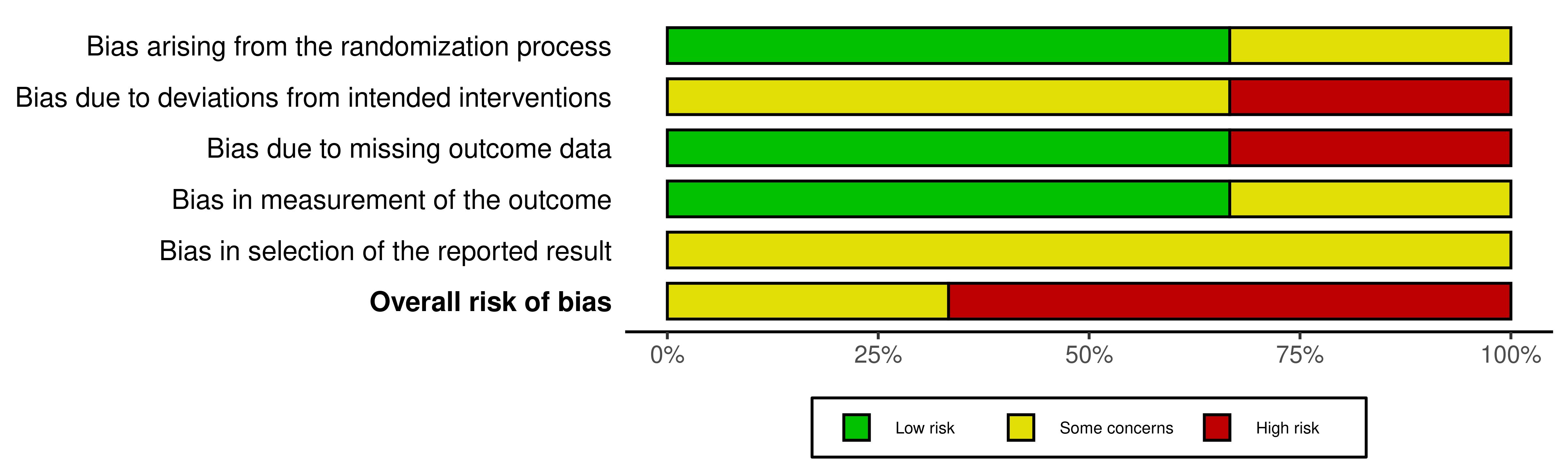 |
| --- | --- |

#### 1h) Time to reach target energy needs

| 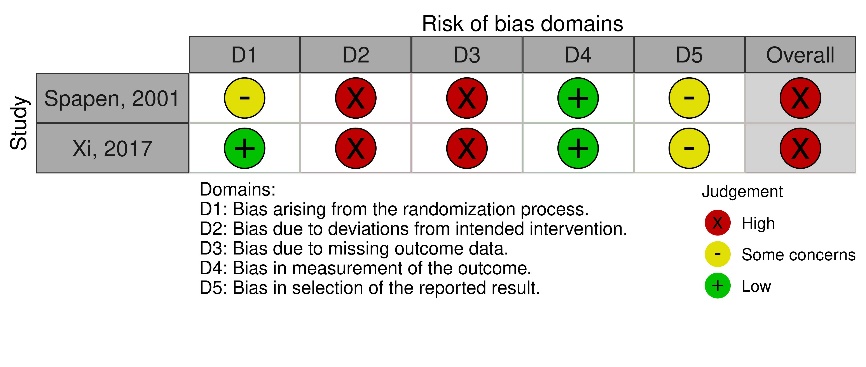 | 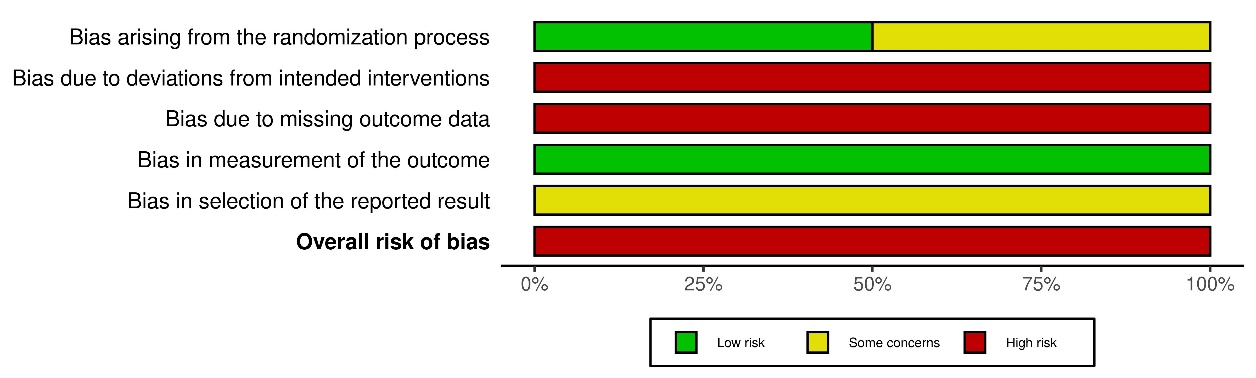 |
| --- | --- |

# PART 3: Results of the meta-analyses and subgroup analyses

# PART 3A: Subgroup analyses

### Table S11: Summary of the results of subgroup analyses

| **Outcome/ Subgroups** | **Trials** | **N (fiber/control)** | **I² (%)** | **RR/MD (95% CI)** | **p-value** | **Test for subgroup difference** |
| --- | --- | --- | --- | --- | --- | --- |
| **Mortality** | 12 | 399/403 | 0 | 0.66 [0.47, 0.92] | 0.01 |  |
| Published before 2000 | 1 | 17/18 | - | 0.85 [0.27, 2.64] | 0.77 | Chi² = 0.21, df = 1 (p = 0.65), I² = 0% |
| Published after 2000 | 11 | 382/385 | 0 | 0.64 [0.46, 0.91] | 0.01 |  |
| Fermentable fiber | 7 | 207/235 | 0 | 0.63 [0.35, 1.14] | 0.13 | Chi² = 0.18, df = 2 (p = 0.92), I² = 0% |
| Non-fermentable fiber | 0 | - | | | |  |
| Mixed fiber | 4 | 177/153 | 0 | 0.69 [0.45, 1.04] | 0.08 |  |
| Unspecified | 1 | 15/15 | - | 0.50 [0.11, 2.33] | 0.38 |  |
| Viscous fiber | 2 | 82/83 | 0 | 0.29 [0.06, 1.33] | 0.11 | Chi² = 1.37, df = 3 (p = 0.71), I² = 0% |
| Non-viscous fiber | 5 | 125/152 | 0 | 0.72 [0.39, 1.36] | 0.32 |  |
| Mixed fiber | 4 | 177/153 | 0 | 0.69 [0.45, 1.04] | 0.08 |  |
| Unspecified | 1 | 15/15 | - | 0.50 [0.11, 2.33] | 0.38 |  |
| Soluble fiber | 6 | 190/217 | 0 | 0.57 [0.29, 1.13] | 0.11 | Chi² = 0.36, df = 2 (p = 0.83), I² = 0% |
| Insoluble fiber | 1 | 17/18 | - | 0.85 [0.27, 2.64] | 0.77 |  |
| Mixed fiber | 5 | 192/168 | 0 | 0.67 [0.45, 1.00] | 0.05 |  |
| daily fiber dose < 20 g | 4 | 194/170 | 0 | 0.66 [0.45, 0.98] | 0.04 | Chi² = 0.11, df = 2 (p = 0.95), I² = 0% |
| daily fiber dose ≥ 20 g | 5 | 147/175 | 0 | 0.59 [0.24, 1.44] | 0.24 |  |
| Unclear | 3 | 58/58 | 0 | 0.72 [0.31, 1.65] | 0.44 |  |
| Low risk of bias | 0 |  | | | | |
| High risk of bias/ some concerns | 12 |  |  |  |  |  |
| age < 50 years | 8 | 212/241 | 0 | 0.66 [0.36, 1.22] | 0.19 | Chi² = 0.00, df = 1 (p = 0.98), I² = 0% |
| age ≥ 50 years | 4 | 187/162 | 0 | 0.66 [0.44, 0.98] | 0.04 |  |
| APACHE II < 17 | 4 | 134/164 | 0 | 0.74 [0.32, 1.73] | 0.49 | Chi² = 0.77, df = 2 (p = 0.68), I² = 0% |
| APACHE II ≥ 17 | 6 | 235/209 | 0 | 0.68 [0.46, 0.99] | 0.04 |  |
| Unclear | 2 | 30/30 | 0 | 0.40 [0.12, 1.32] | 0.13 |  |
| Medical ICU | 2 | 23/22 | 0 | 0.39 [0.12, 1.26] | 0.12 | Chi² = 1.17, df = 3 (p = 0.76), I² = 0% |
| Surgical ICU | 2 | 46/75 | 0 | 0.94 [0.23, 3.74] | 0.92 |  |
| Mixed ICU | 2 | 164/140 | 0 | 0.70 [0.46, 1.07] | 0.10 |  |
| Unclear | 6 | 166/166 | 0 | 0.61 [0.31, 1.20] | 0.15 |  |
| intervention start ≤ 24h | 4 | 99/127 | 0 | 0.66 [0.33, 1.33] | 0.24 | Chi² = 0.04, df = 2 (p = 0.98), I² = 0% |
| intervention start ≤ 48h | 3 | 204/181 | 0 | 0.64 [0.41, 1.00] | 0.05 |  |
| Unclear | 5 | 96/95 | 0 | 0.70 [0.34, 1.42] | 0.32 |  |
| minimum duration of intervention < 6 days | 2 | 27/27 | 0 | 0.50 [0.15, 1.71] | 0.27 | Chi² = 0.70, df = 2 (p = 0.71), I² = 0% |
| minimum duration of intervention ≥ 6 days | 6 | 249/226 | 0 | 0.63 [0.43, 0.93] | 0.02 |  |
| Unclear | 4 | 123/150 | 0 | 0.84 [0.41, 1.73] | 0.64 |  |
| co-intervention with immunonutrition | 2 | 164/140 | 0 | 0.70 [0.46, 1.07] | 0.10 | Chi² = 0.24, df = 1 (p = 0.62), I² = 0% |
| fiber only | 10 | 235/263 | 0 | 0.59 [0.34, 1.02] | 0.06 |  |
| Industry funding | 3 | 152/127 | 0 | 0.65 [0.42, 1.01] | 0.06 | Chi² = 0.34, df = 2 (p = 0.84), I² = 0% |
| Non-industry funding | 4 | 121/151 | 0 | 0.54 [0.22, 1.33] | 0.18 |  |
| Unclear funding | 5 | 126/125 | 0 | 0.74 [0.41, 1.34] | 0.32 |  |
| Standard formula in control group | 8 | 189/187 | 0 | 0.47 [0.24, 0.90] | 0.02 | Chi² = 1.43, df = 1 (p = 0.23), I² = 29.9% |
| Non-standard formula in control group | 4 | 210/216 | 0 | 0.74 [0.51, 1.09] | 0.12 |  |

| **Outcome/ Subgroups** | **Trials** | **N (fiber/control)** | **I² (%)** | **RR/MD (95% CI)** | **p-value** | **Test for subgroup difference** |
| --- | --- | --- | --- | --- | --- | --- |
| **Diarrhea** | 11 | 396/369 | 51 | 0.70 [0.51, 0.96] | 0.03 |  |
| Published before 2000 | 2 | 80/79 | 0 | 1.04 [0.73, 1.46] | 0.84 | Chi² = 4.86, df = 1 (p = 0.03), I² = 79.4% |
| Published after 2000 | 9 | 316/290 | 43 | 0.59 [0.40, 0.85] | 0.005 |  |
| Fermentable fiber | 7 | 256/251 | 57 | 0.70 [0.47, 1.05] | 0.08 | Chi² = 3.75, df = 2 (p = 0.15), I² = 46.7% |
| Non-fermentable fiber | 0 | - | | | |  |
| Mixed fiber | 2 | 77/77 | 0 | 0.57 [0.40, 0.81] | 0.002 |  |
| Unspecified | 2 | 63/41 | - | 3.67 [0.53, 25.26] | 0.19 |  |
| Viscous fiber | 2 | 97/96 | 66 | 0.70 [0.32, 1.50] | 0.36 | Chi² = 3.61, df = 3 (p = 0.31), I² = 16.8% |
| Non-viscous fiber | 5 | 159/155 | 64 | 0.66 [0.36, 1.19] | 0.16 |  |
| Mixed fiber | 3 | 107/107 | 0 | 0.57 [0.40, 0.81] | 0.002 |  |
| Unspecified | 1 | 33/11 | - | 3.67 [0.53, 25.26] | 0.19 |  |
| Soluble fiber | 6 | 211/205 | 58 | 0.61 [0.39, 0.97] | 0.03 | Chi² = 3.81, df = 2 (p = 0.15), I² = 47% |
| Insoluble fiber | 1 | 45/46 | - | 1.26 [0.69, 2.31] | 0.46 |  |
| Mixed fiber | 4 | 140/118 | 47 | 0.69 [0.33, 1.45] | 0.33 |  |
| daily fiber dose < 20 g | 5 | 175/149 | 33 | 0.96 [0.61, 1.50] | 0.84 | Chi² = 3.37, df = 2 (p = 0.19), I² = 40.7% |
| daily fiber dose ≥ 20 g | 5 | 208/208 | 53 | 0.59 [0.37, 0.96] | 0.04 |  |
| Unclear | 1 | 13/12 | - | 0.50 [0.27, 0.93] | 0.03 |  |
| Low risk of bias | 0 |  | | | | |
| High risk of bias/ some concerns | 11 |  |  |  |  |  |
| age < 50 years | 6 | 213/211 | 59 | 0.68 [0.40, 1.15] | 0.15 | Chi² = 0.00, df = 1 (p = 0.98), I² = 0% |
| age ≥ 50 years | 5 | 183/158 | 53 | 0.69 [0.43, 1.09] | 0.11 |  |
| APACHE II < 17 | 3 | 155/134 | 51 | 0.64 [0.32, 1.28] | 0.21 | Chi² = 8.77, df = 2 (p = 0.01), I² = 77.2% |
| APACHE II ≥ 17 | 5 | 126/123 | 6 | 0.42 [0.25, 0.71] | 0.001 |  |
| Unclear | 3 | 115/112 | 0 | 1.00 [0.76, 1.32] | 1.00 |  |
| Medical ICU | 2 | 73/72 | 0 | 0.56 [0.40, 0.77] | 0.0004 | Chi² = 10.22, df = 3 (p = 0.02), I² = 70.6% |
| Surgical ICU | 1 | 17/17 | - | 0.50 [0.18, 1.35] | 0.17 |  |
| Mixed ICU | 5 | 190/165 | 22 | 1.02 [0.73, 1.42] | 0.93 |  |
| Unclear | 3 | 116/115 | 0 | 0.37 [0.18, 0.74] | 0.005 |  |
| intervention start ≤ 24h | 3 | 90/87 | 60 | 0.63 [0.31, 1.29] | 0.20 | Chi² = 4.14, df = 3 (p = 0.25), I² = 27.6% |
| intervention start ≤ 48h | 2 | 122/123 | 0 | 0.55 [0.39, 0.78] | 0.0008 |  |
| intervention start ≥ 48h | 1 | 33/11 | - | 3.67 [0.53, 25.26] | 0.19 |  |
| Unclear | 5 | 151/148 | 57 | 0.76 [0.43, 1.34] | 0.34 |  |
| minimum duration of intervention < 6 days | 4 | 127/126 | 18 | 0.95 [0.65, 1.39] | 0.79 | Chi² = 3.43, df = 2 (p = 0.18), I² = 41.6% |
| minimum duration of intervention ≥ 6 days | 4 | 143/119 | 56 | 0.71 [0.41, 1.26] | 0.24 |  |
| Unclear | 3 | 126/124 | 48 | 0.35 [0.13, 0.98] | 0.05 |  |
| co-intervention with immunonutrition | 1 | 42/42 | - | 0.09 [0.01, 1.59] | 0.10 | Chi² = 1.97, df = 1 (p = 0.16), I² = 49.3% |
| fiber only | 10 | 354/327 | 51 | 0.72 [0.52, 0.98] | 0.04 |  |
| Industry funding | 4 | 98/73 | 52 | 0.75 [0.43, 1.31] | 0.31 | Chi² = 0.35, df = 2 (p = 0.84), I² = 0% |
| Non-industry funding | 2 | 97/96 | 64 | 0.70 [0.32, 1.49] | 0.35 |  |
| Unclear funding | 5 | 201/200 | 68 | 0.56 [0.26, 1.22] | 0.14 |  |
| Standard formula in control group | 10 | 354/327 | 51 | 0.72 [0.52, 0.98] | 0.04 | Chi² = 1.97, df = 1 (p = 0.16), I² = 49.3% |
| Non-standard formula in control group | 1 | 42/42 | 51 | 0.09 [0.01, 1.59] | 0.10 |  |

### Table S12: Calculation of daily fiber doses

| **Reference No** | **Author, year** | **Daily fiber dose** | **Variables** | **Calculation*** |
| --- | --- | --- | --- | --- |
| [38] | Hart, 1988 | 7 g/d | - | Fiber/d reported |
| [39] | Dobb, 1990 | 21 – 42 g/d | Fiber: 21g/l  Feed volumes from day 3: mean volume of 1000-2000 ml | $Daily fiber dose1=1000 ml \times\left( {21g}/{1000 ml} \right)= 21 g$  $Daily fiber dose2=2000 ml \times\left( {21g}/{1000 ml} \right)= 42 g$ |
| [47] | Celaya, 1992 | NI | NI | - |
| [35] | Caparrós, 2000 | 11.7 g/d | Fiber: 13.5g/1500ml  Caloric density: 1.25kcal/ml  Administered caloric intake day 3: 1625 (1137 – 1828) kcal | $Daily fiber dose= \frac{1625 kcal}{1.25 kcal/ml} \times13.5 g/1500ml=11.7 g$ |
| [41] | Schultz, 2000 | 15.8 – 17.3 g/d | - | Fiber/d reported |
| [43] | Spapen, 2001 | NI | NI | - |
| [40] | Rushdi, 2004 | 22-24 g/d | - | Fiber/d reported |
| [37] | Palmese, 2006 | 10.6 g/d | - | Fiber/d reported |
| [49] | Karakan, 2007 | 24 g/d | - | Fiber/d reported |
| [46] | Spindler-Vesel, 2007 | 20.68 g/d | Fiber: 22 g/l  Total feed volumes day 2 to 4: 2820 (2300 – 3100) ml | $Daily fiber dose=\frac{2820 ml \times\left( {22g}/{1000 ml} \right)}{3 days}= 20.68 g$ |
| [45] | Chittawatanarat,  2010 | 22.65 g/d | Fiber: 15.1 g/l  Caloric density: 1000 kcal/l  Mean caloric intake day 6: 1500 kcal | $Daily fiber dose= \frac{1500 kcal}{{1000 kcal}/l} \times15.1 g/l=22.65 g$ |
| [52] | Zavertailo, 2010 | NI | NI | - |
| [53] | Aytünür | 18 g/d | Fiber: 10.6 g/l  Caloric density: 1.1 kcal/l  Max. calories per day: 1867 ± 120.18 ml | $Daily fiber dose= \frac{1867 kcal}{{1.1 kcal}/{ml}} \times10.6 g/l=18 g$ |
| [51] | Wang, 2014 | NI | NI | - |
| [36] | Kamarul Zaman, 2016 | 14.8 g/d | - | Fiber/d reported |
| [44] | Yagmurdur, 2016 | 28 g/d | - | Fiber/d reported |
| [54] | Xi, 2017 | 24 g/d | - | Fiber/d reported |
| [50] | Fazilaty, 2018 | 3 g/d | - | Fiber/d reported |
| [42] | Freedberg, 2020 | 11 g/d | - | Fiber/d reported |
| [48] | Chen, 2021 | 20 g/d | - | Fiber/d reported |
| **Median value** | | **20 g/d** |  |  |

In studies reporting the evolution of feed volume or caloric intake over the study period, daily fiber dose calculations for subgroup analysis were based on maximum observed values. We associated maximum values with the achievement of caloric goals, thereby ensuring that the calculated fiber doses are valid for most of the study period.

If median (IQR) or mean + SD of caloric intake/feed volumes were given, median or mean were used for the calculation.

When a range of daily fiber dose was reported, both values were used for the calculation of the median value of all fiber doses.

### Figure S2: Overall mortality (subgroup analyses)

#### 2a) Publication date


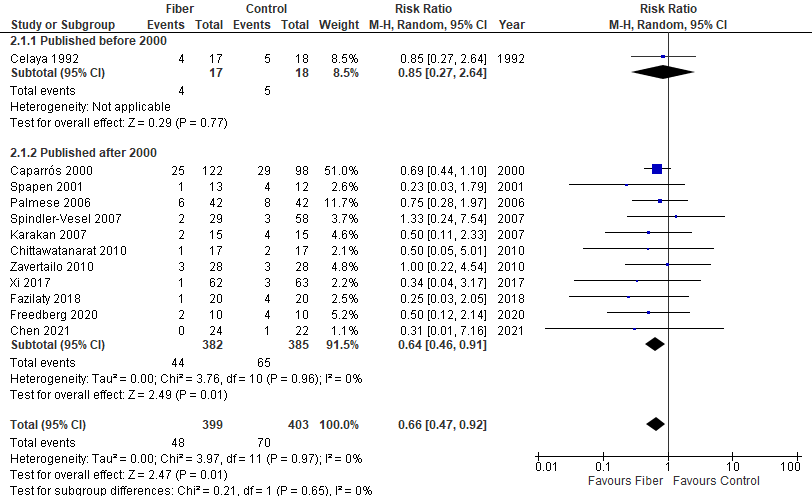


#### 2b) Fermentability


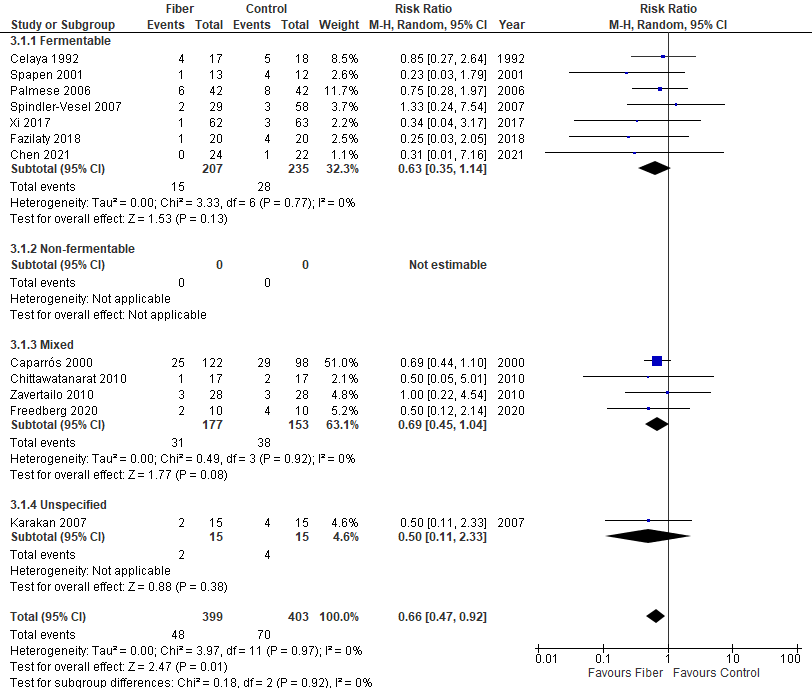


#### 2c) Viscosity


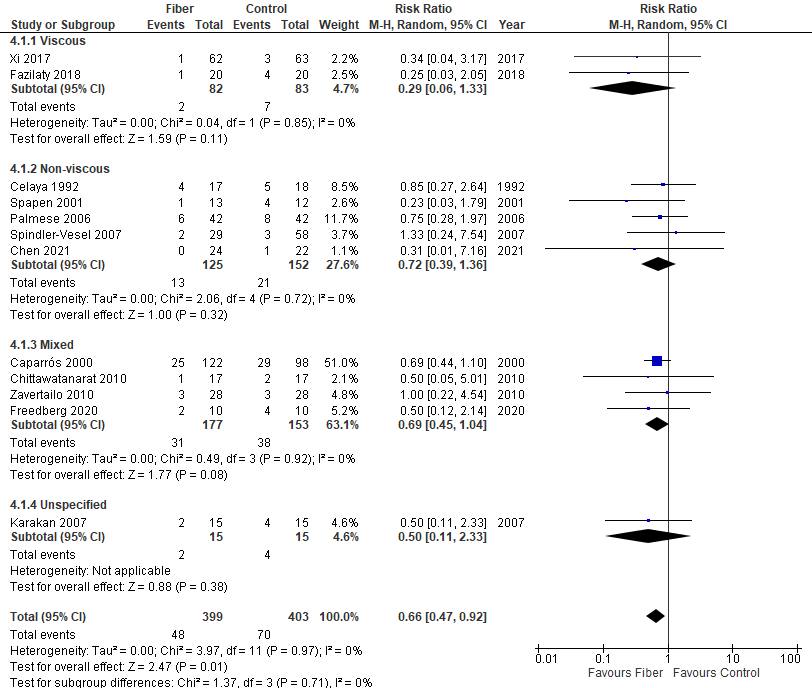


#### 2d) Solubility


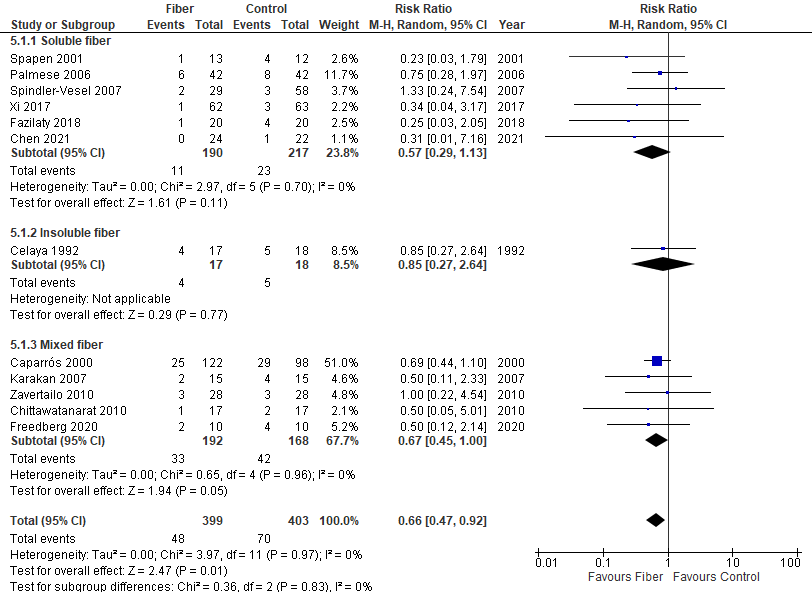


#### 2e) Daily fiber dose


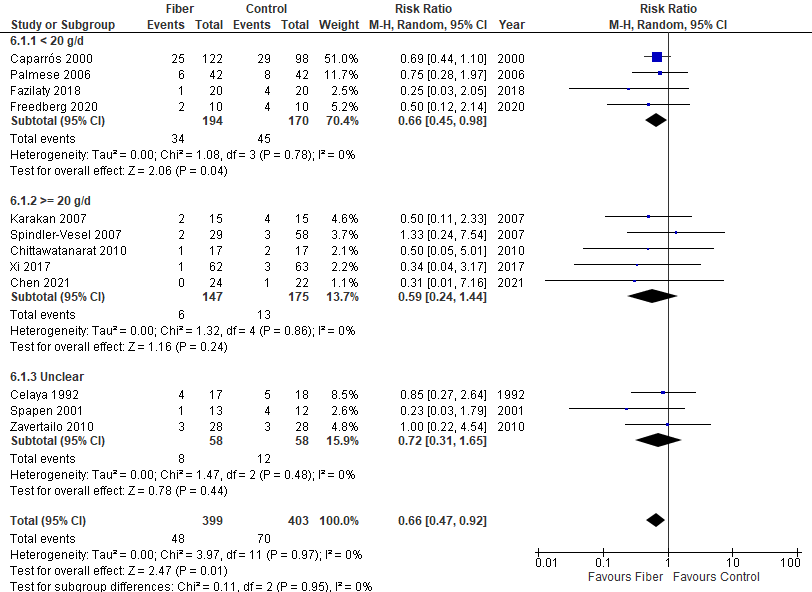


#### 2f) Age


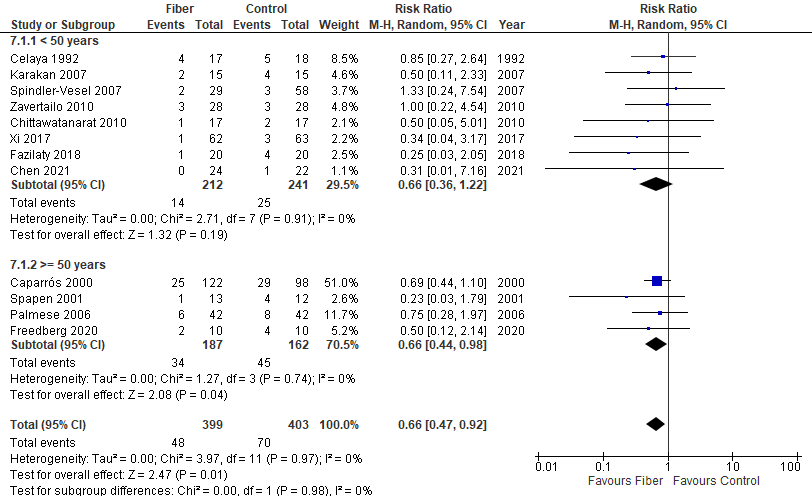


#### 2g) Disease severity


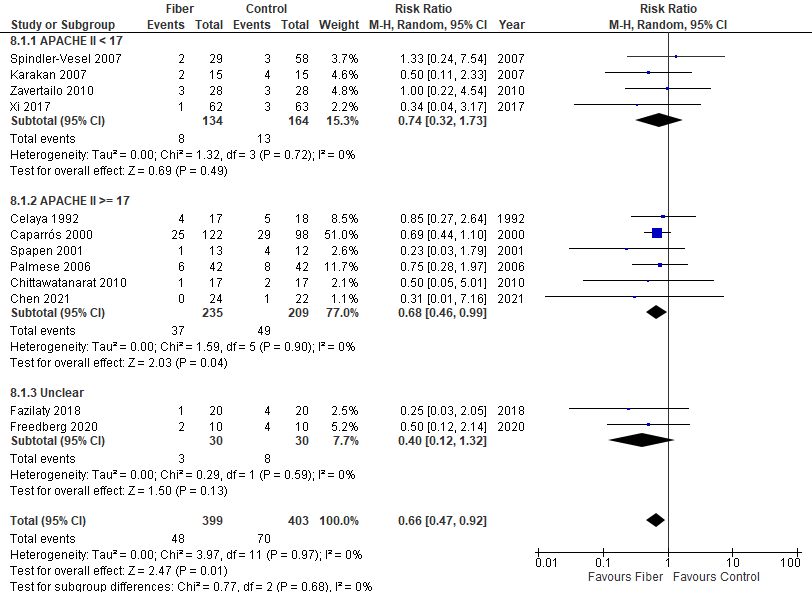


#### 2h) ICU admission type


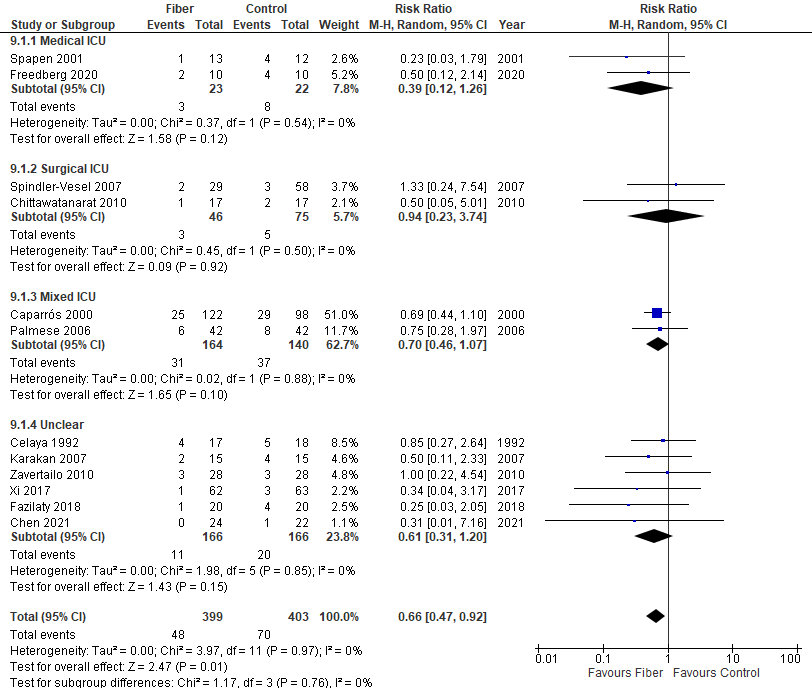


#### 2i) Intervention start


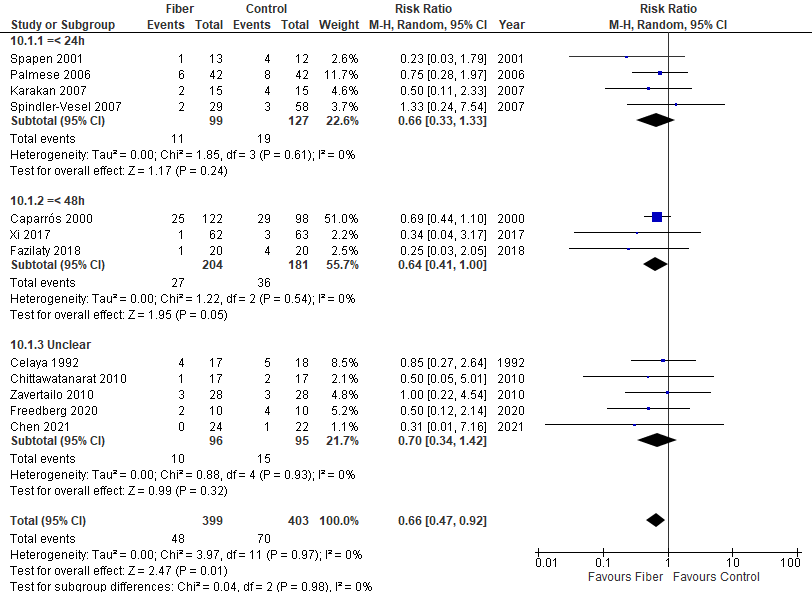


#### 2j) Minimum duration of intervention


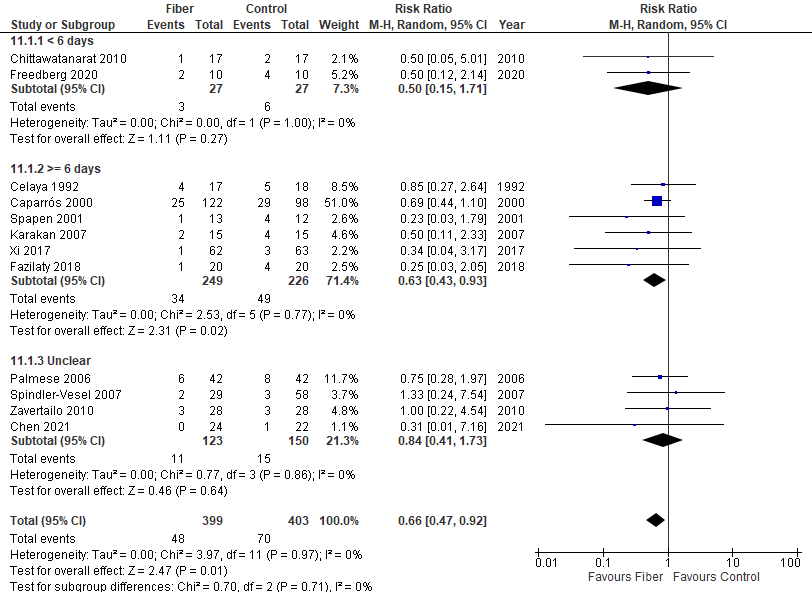


#### 2k) Co-intervention with immunonutrition


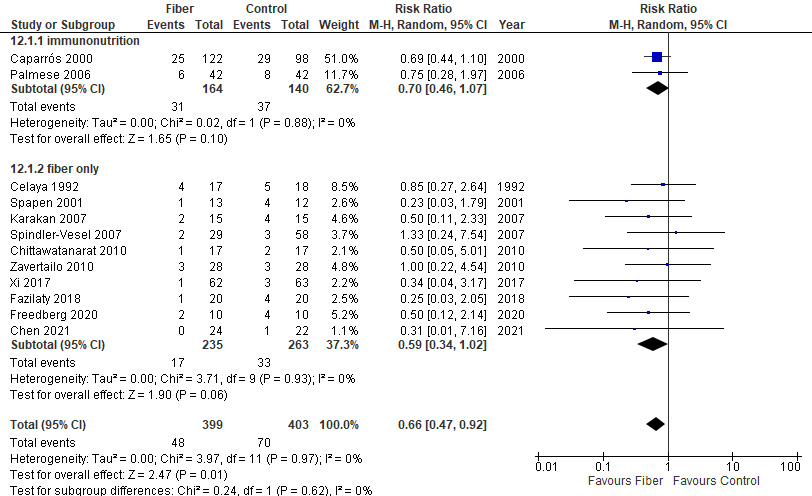


#### 2l) Funding source


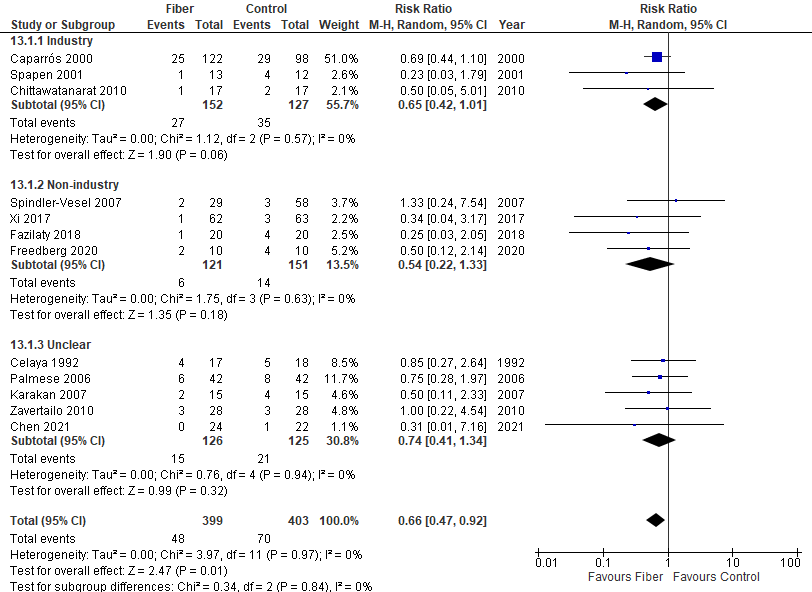


#### 2m) Type of control group


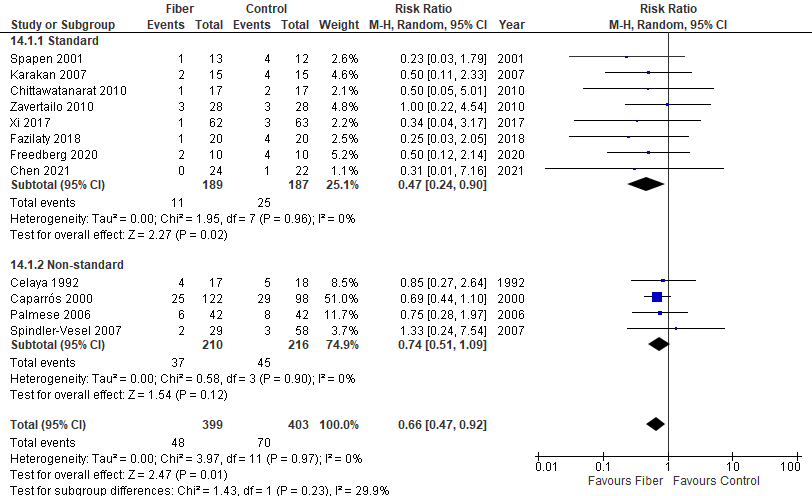


### Figure S3: Diarrhea incidence (subgroup analyses)

#### 3a) Publication date


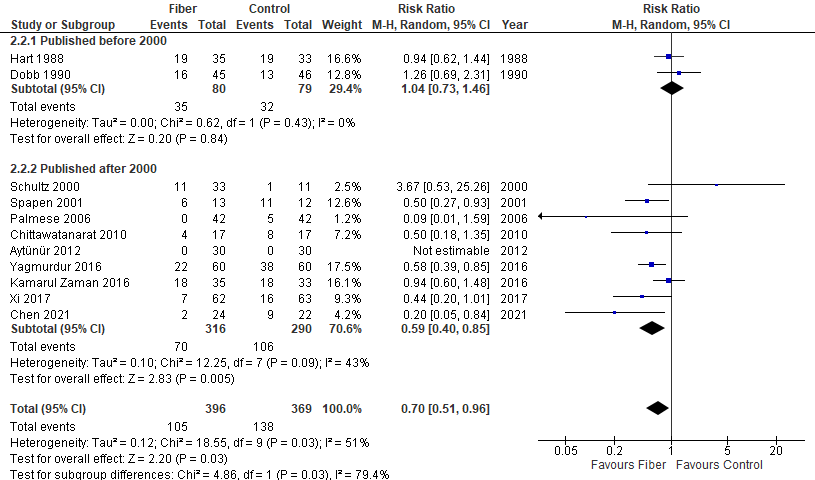


#### 3b) Fermentability


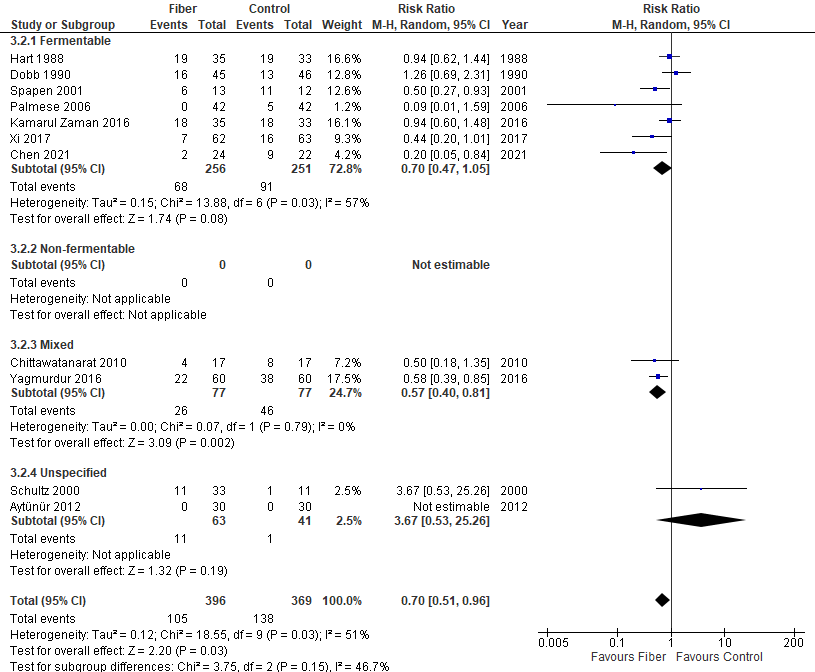


#### 3c) Viscosity


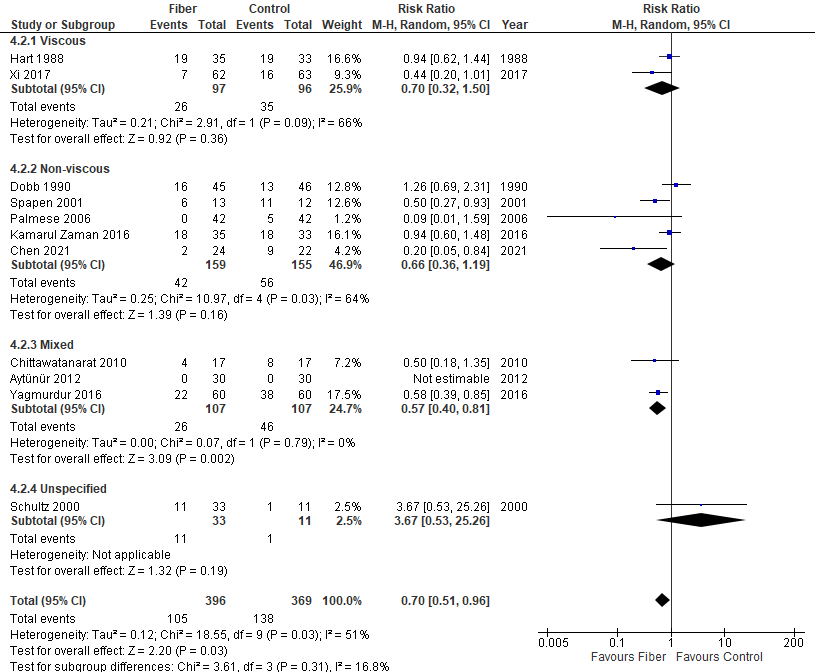


#### 3d) Solubility


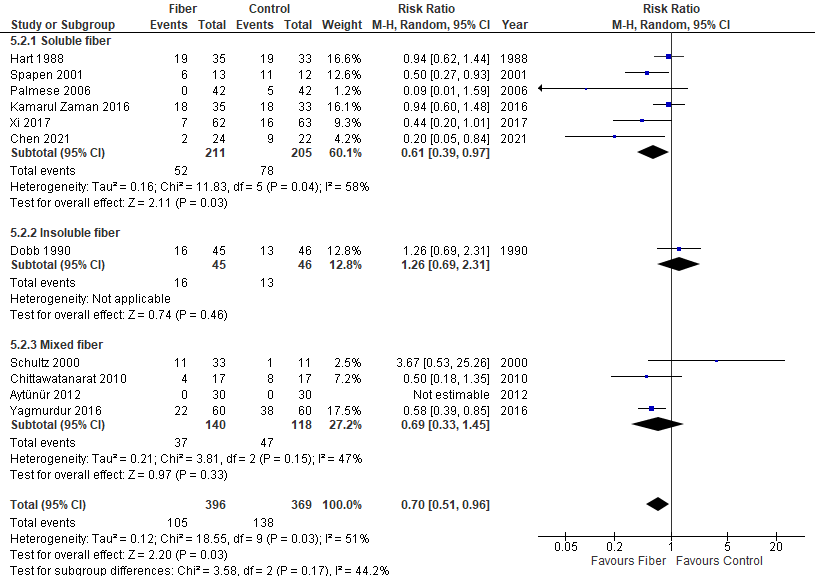


#### 3e) Daily fiber dose


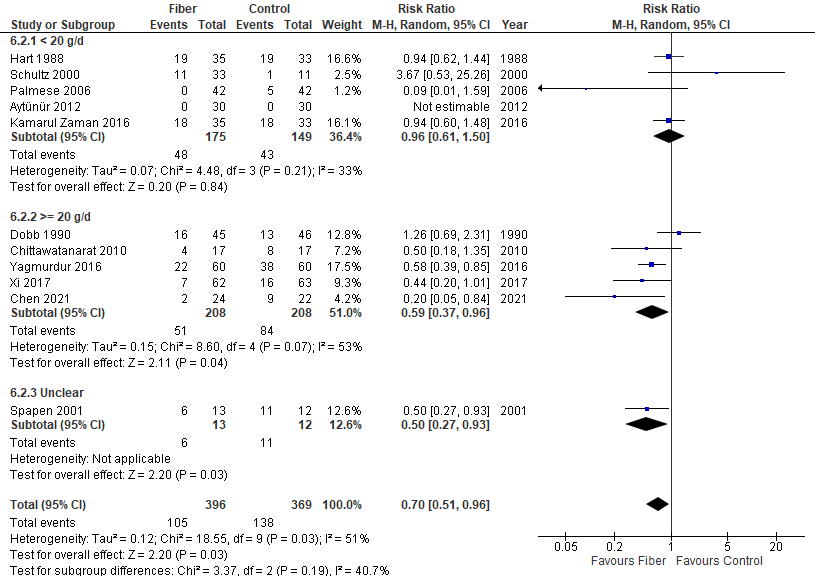


#### 3f) Age


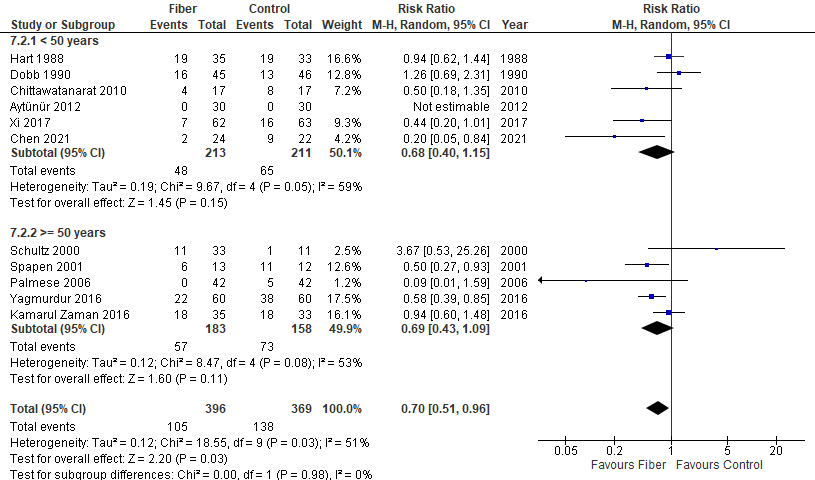


#### 3g) Disease severity


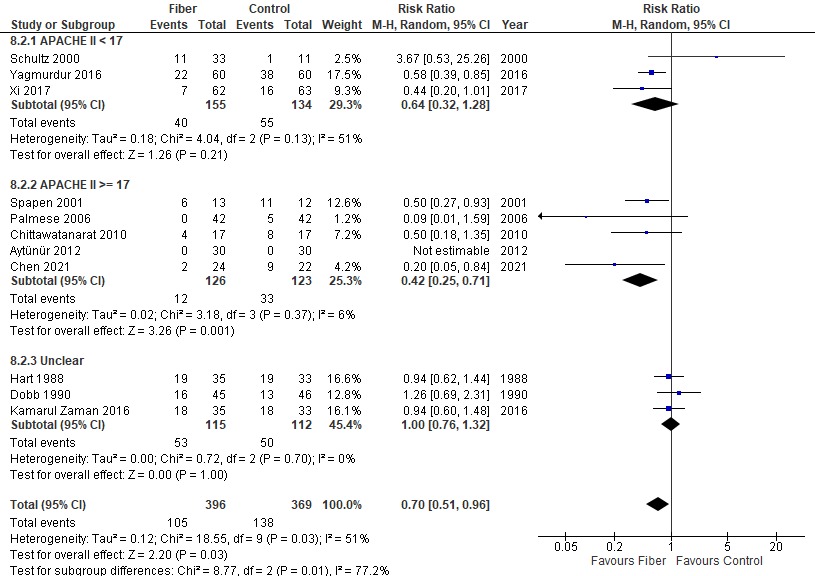


#### 3h) ICU admission type


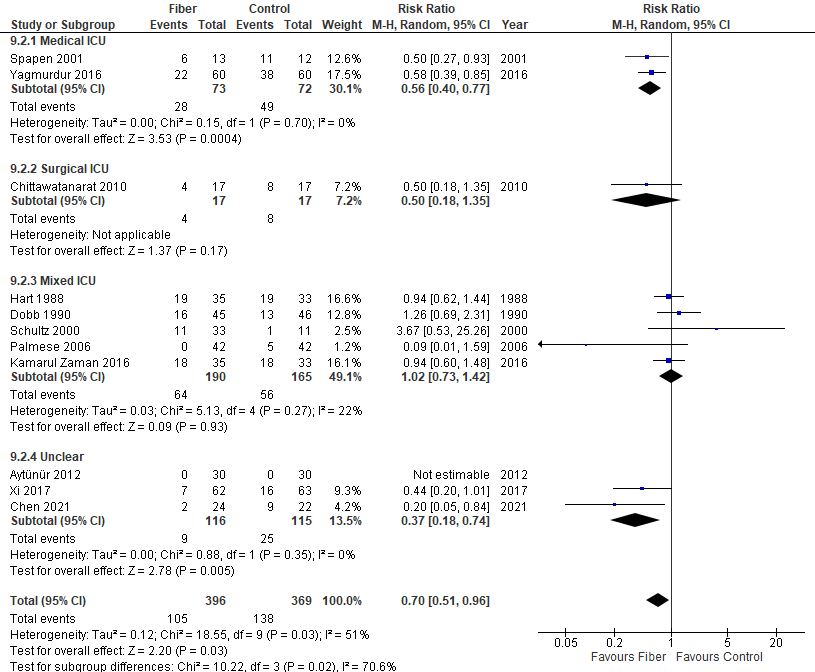


#### 3i) Intervention start


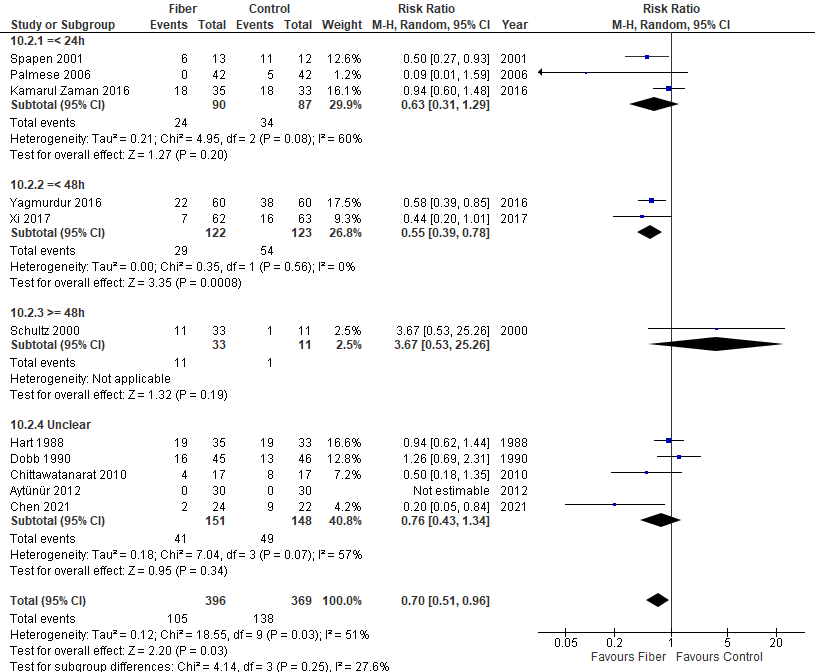


#### 3j) Minimum duration of intervention


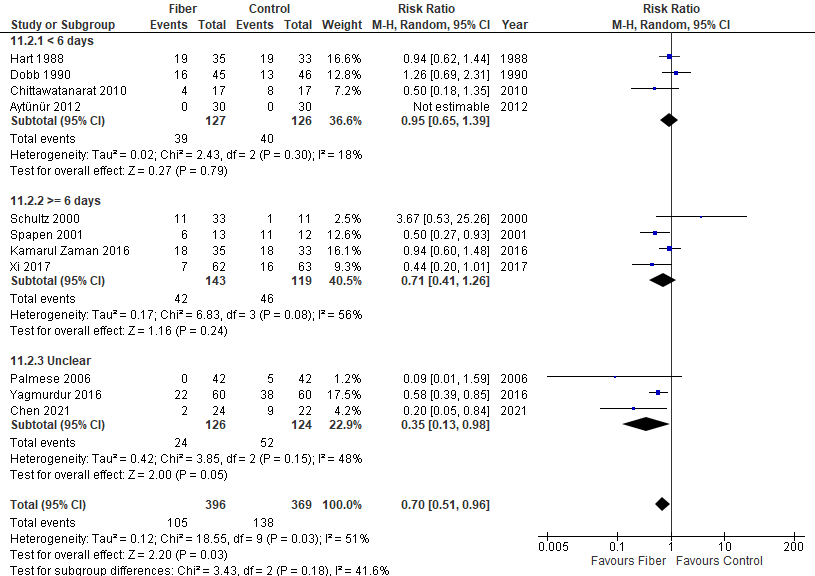


#### 3k) Co-intervention with immunonutrition


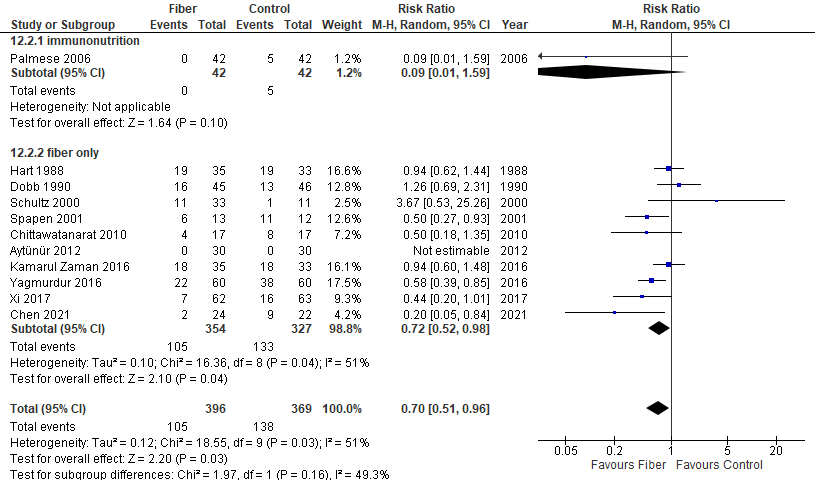


#### 3l) Funding source


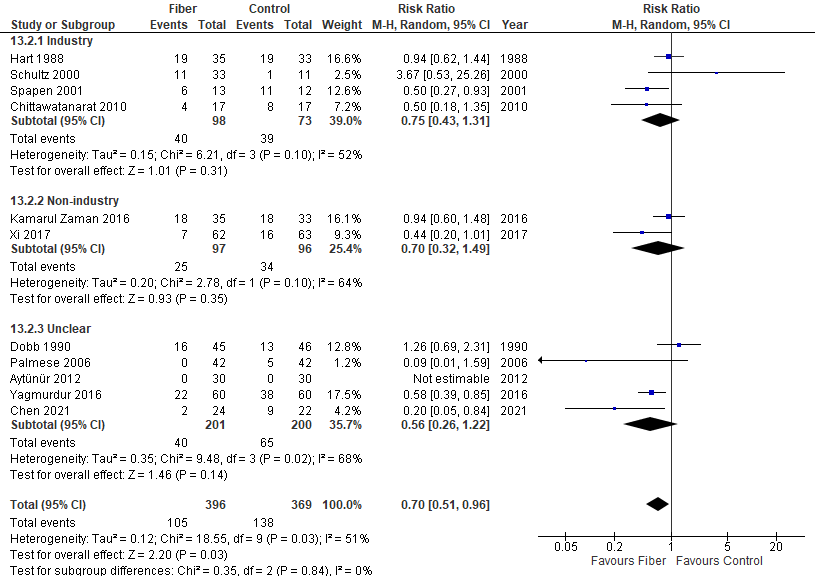


#### 3m) Type of control group


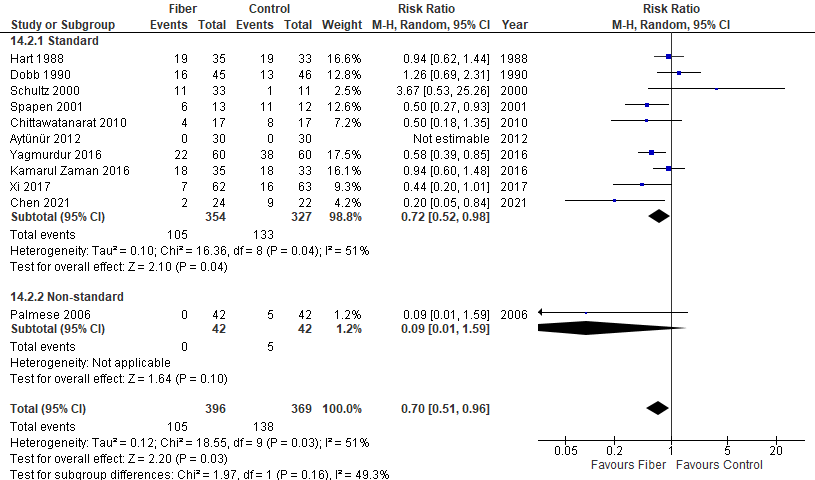


# PART 3B: Overall meta-analyses

### Figure S4: Diarrhea score


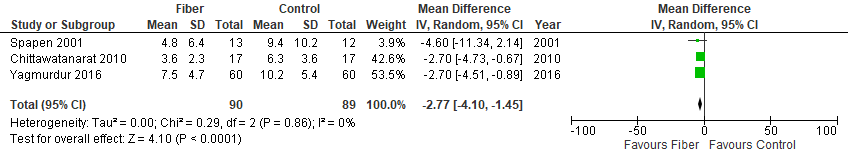


### Figure S5: Other GI complications

#### 5a) Patients with at least one GI complication


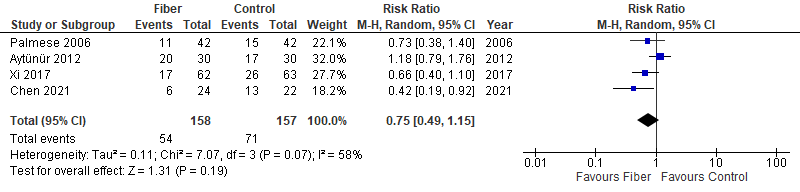


#### 5b) Abdominal distension


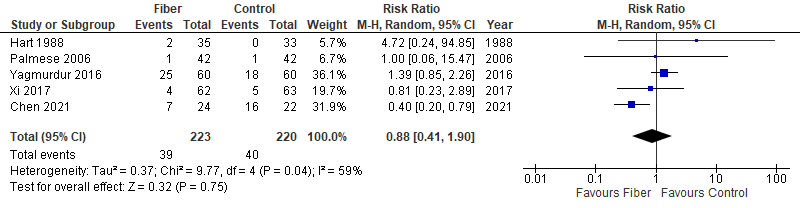


#### 5c) Vomiting


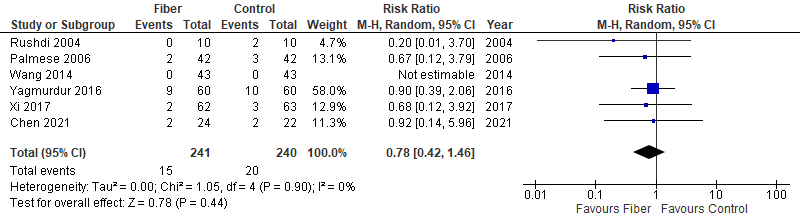


#### 5d) Regurgitation


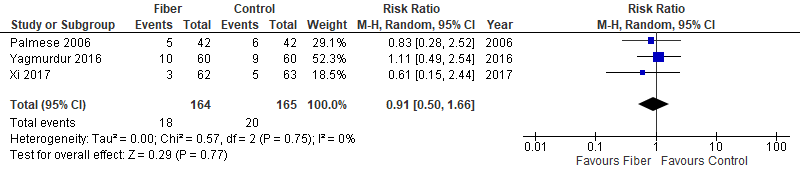


#### 5e) GI bleeding


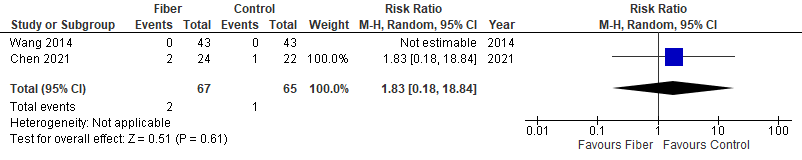


#### 5f) Constipation


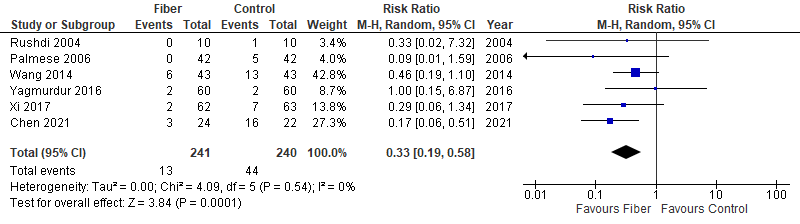


### Figure S6: Infectious complications

6a) Patients with at least 1 infectious complication


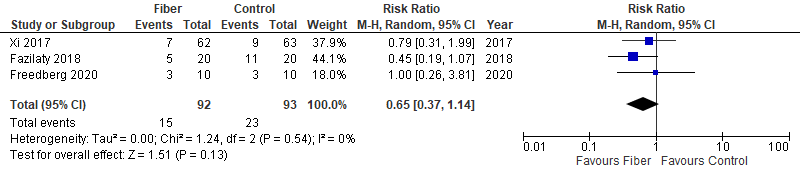


#### 6b) Pneumonia


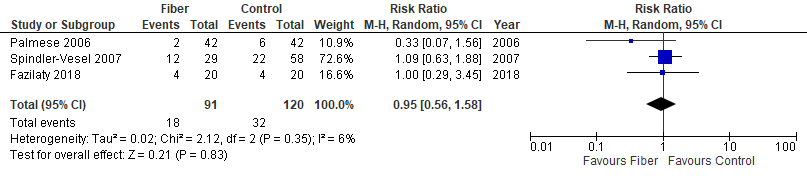


#### 6c) Urinary tract infection


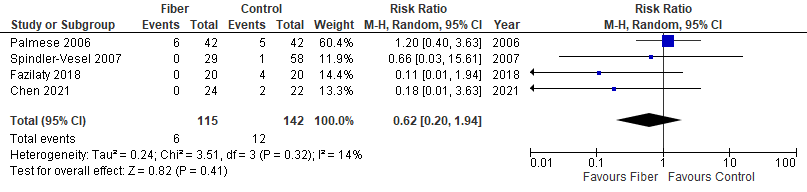


#### 6d) Intra-abdominal infection


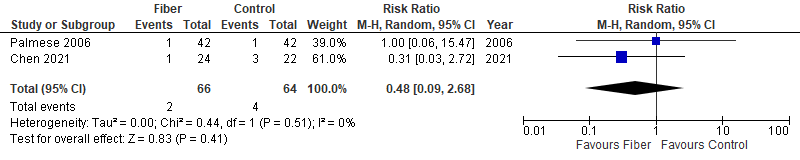


#### 6e) Sepsis


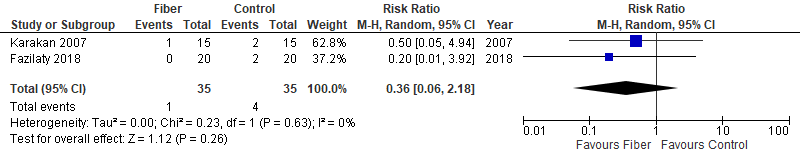


#### 6f) Vascular infection


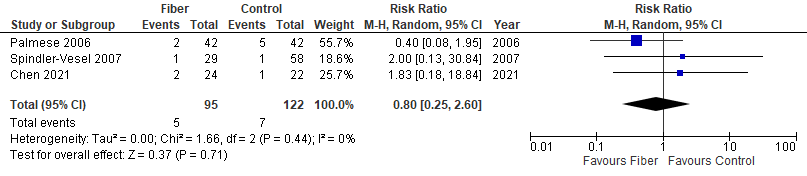


#### 6g) Wound infection


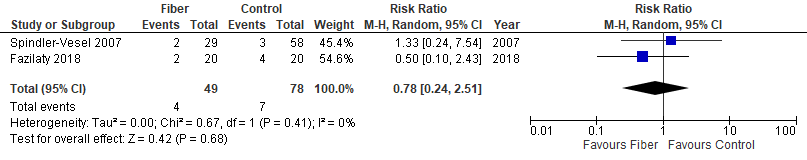


#### 6h) Bacteremia


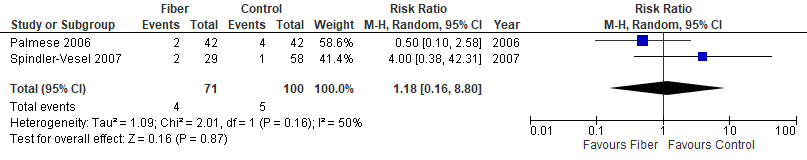


### Figure S7: Duration of mechanical ventilation


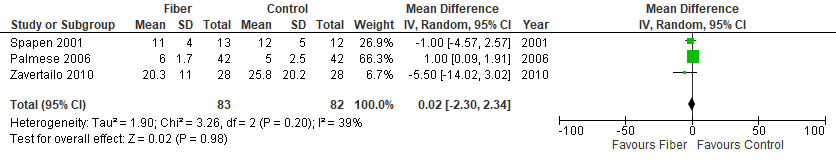


### Figure S8: Time to reach target energy needs


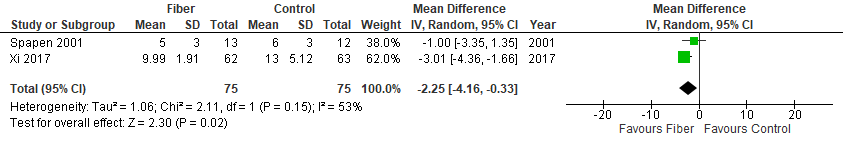


# PART 3C: Funnel plots

### Figure S9: Mortality


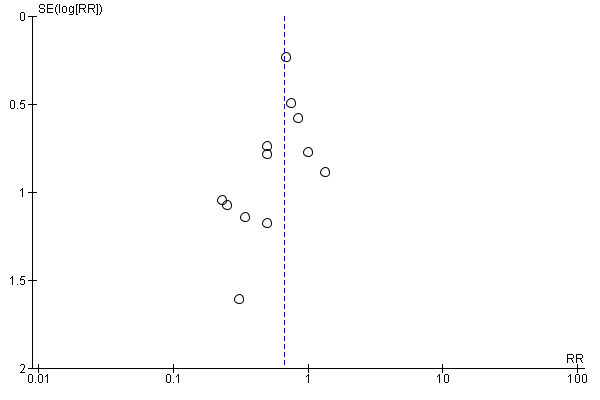

Egger’s test: p = 0.1430498

### Figure S10: Diarrhea

#### 10a) Diarrhea incidence


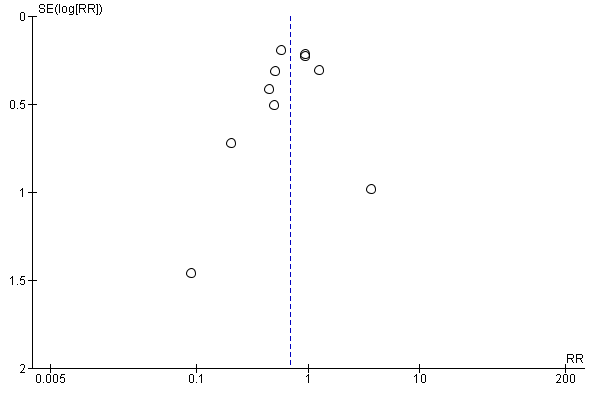

Egger’s test: p = 0.4083957

#### 10b) Diarrhea score


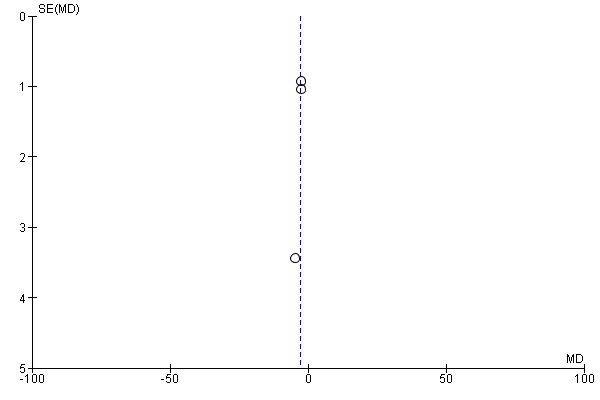


### Figure S11: Other GI complications

#### 11a) Patients with at least 1 GI complication


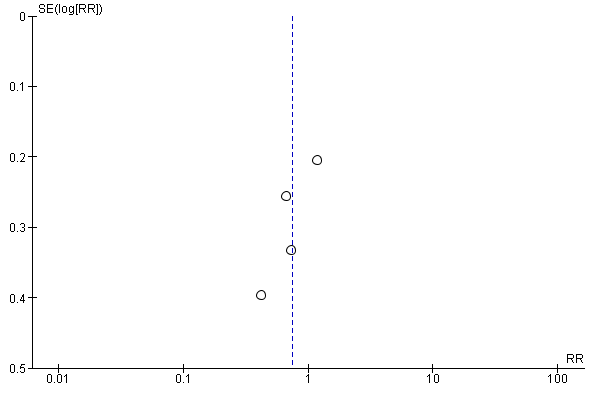


#### 11b) Abdominal distension


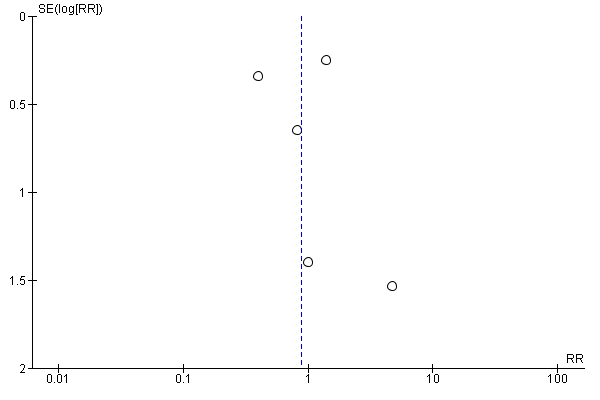


#### 11c) Vomiting


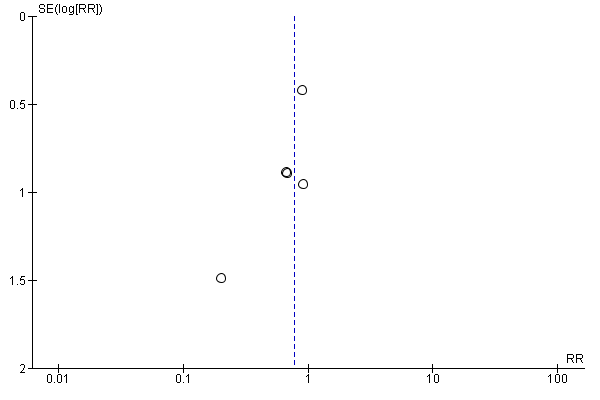


#### 11d) Constipation


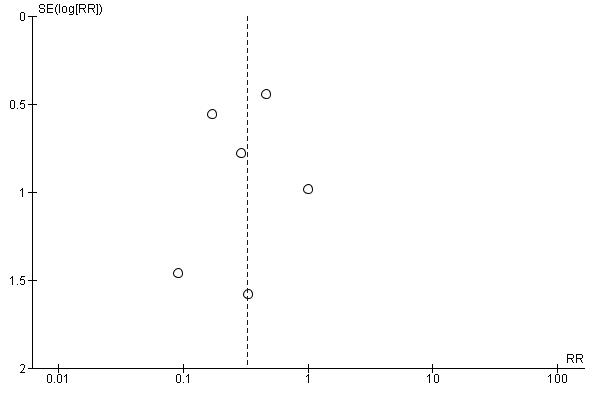


#### 11e) GI bleeding


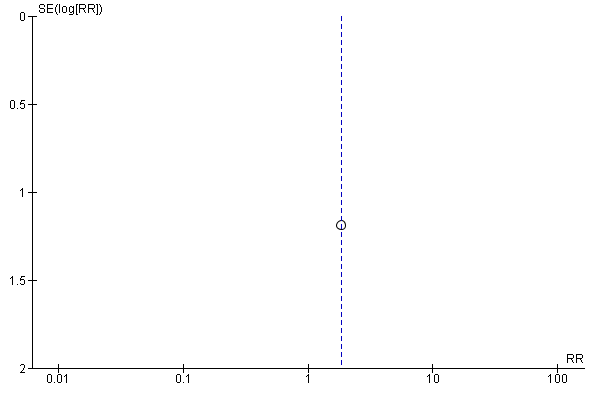


#### 11f) Regurgitation


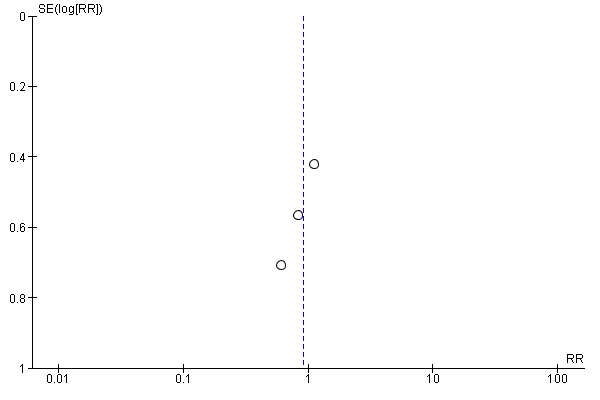


### Figure S12: ICU length of stay

**
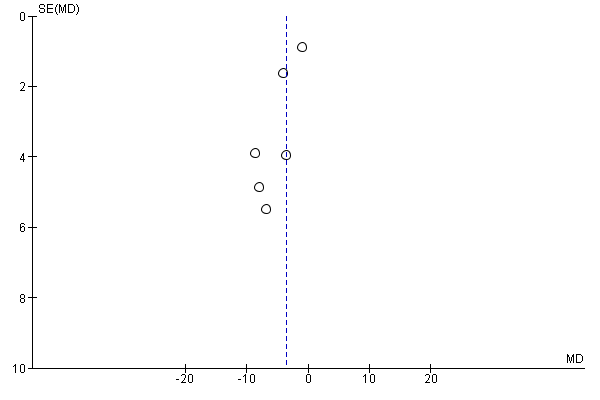
**

### Figure S13: Hospital length of stay


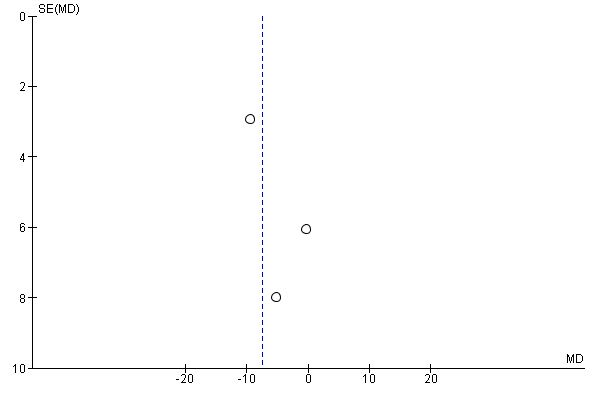


### Figure S14: Infectious complications

#### 14a) Patients with at least 1 infectious complication


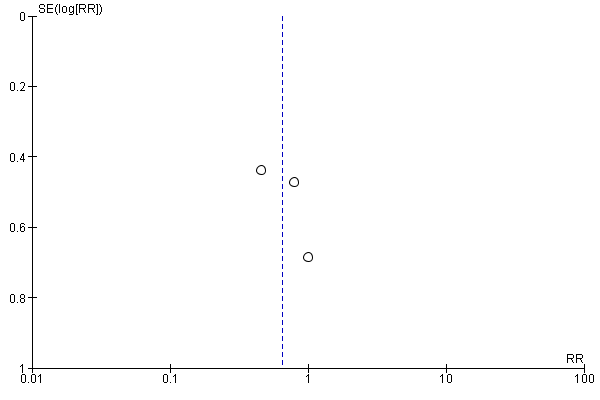


#### 14b) Pneumonia


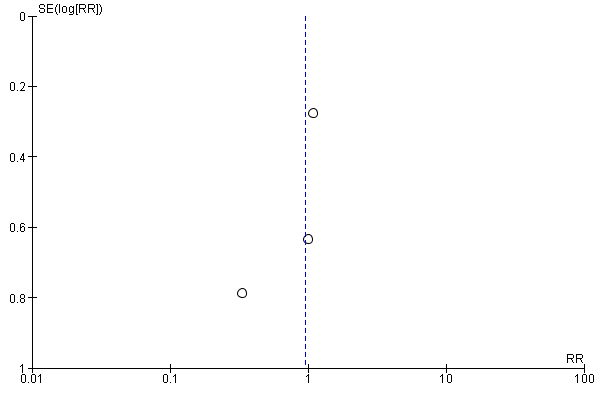


#### 14c) Urinary tract infection


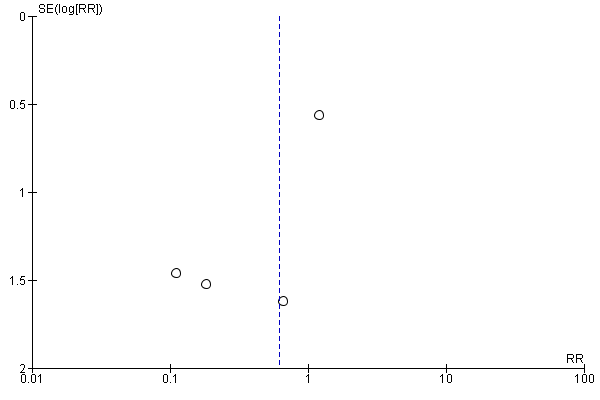


#### 14d) Intra-abdominal infection


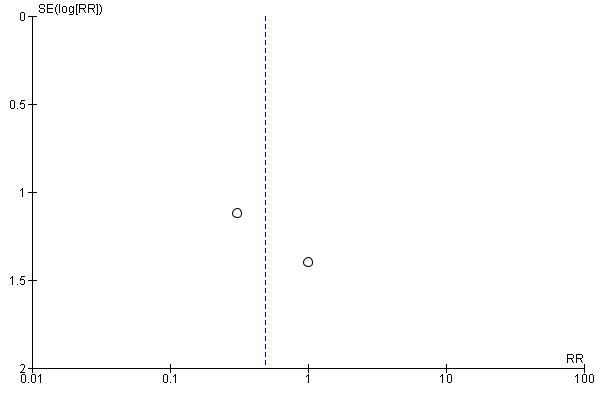


#### 14e) Sepsis


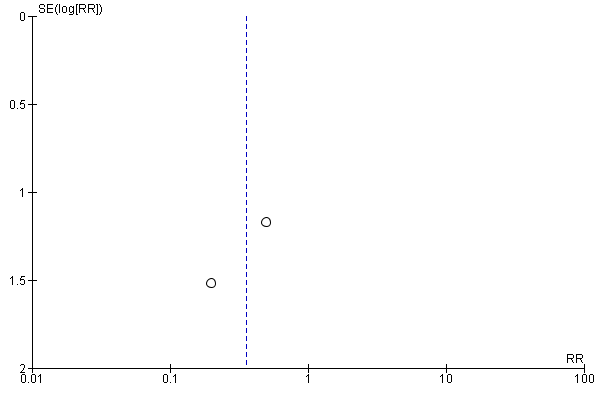


#### 14f) Vascular infection


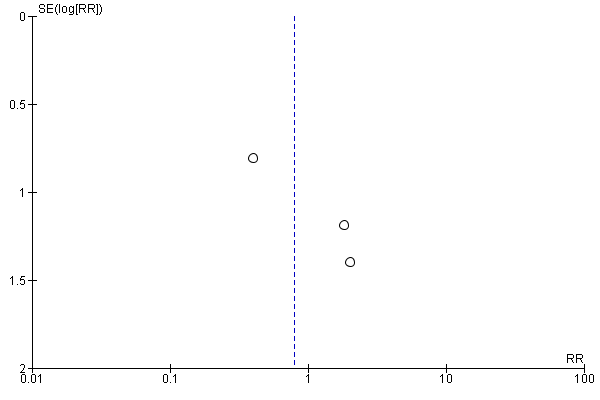


#### 14g) Wound infection


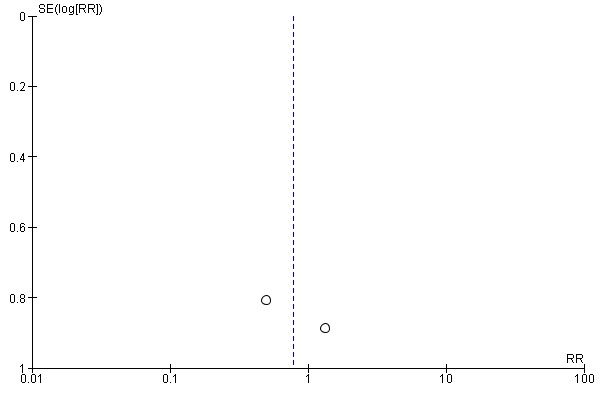


#### 14h) Bacteremia


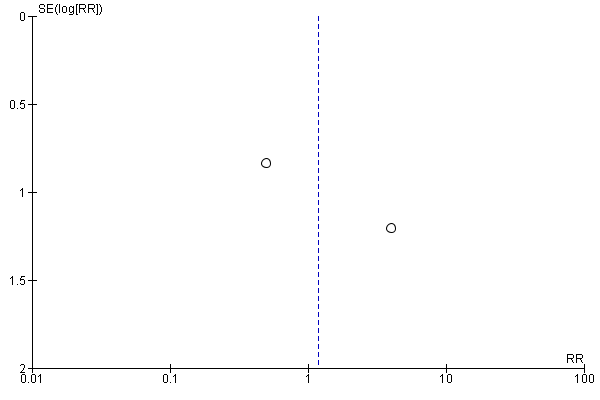


### Figure S15: Duration of mechanical ventilation


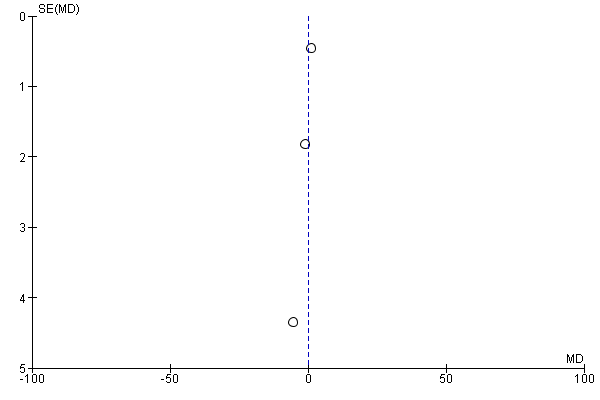


### Figure S16: Time to reach target energy needs


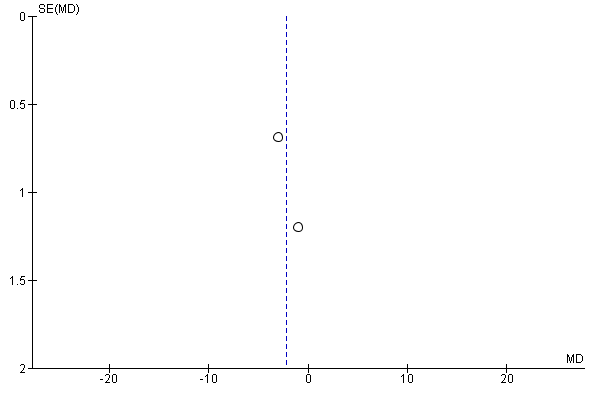


# PART 4: Trial Sequential Analysis

### Fig. S17: Diarrhea incidence

#### a) RRR = 25%


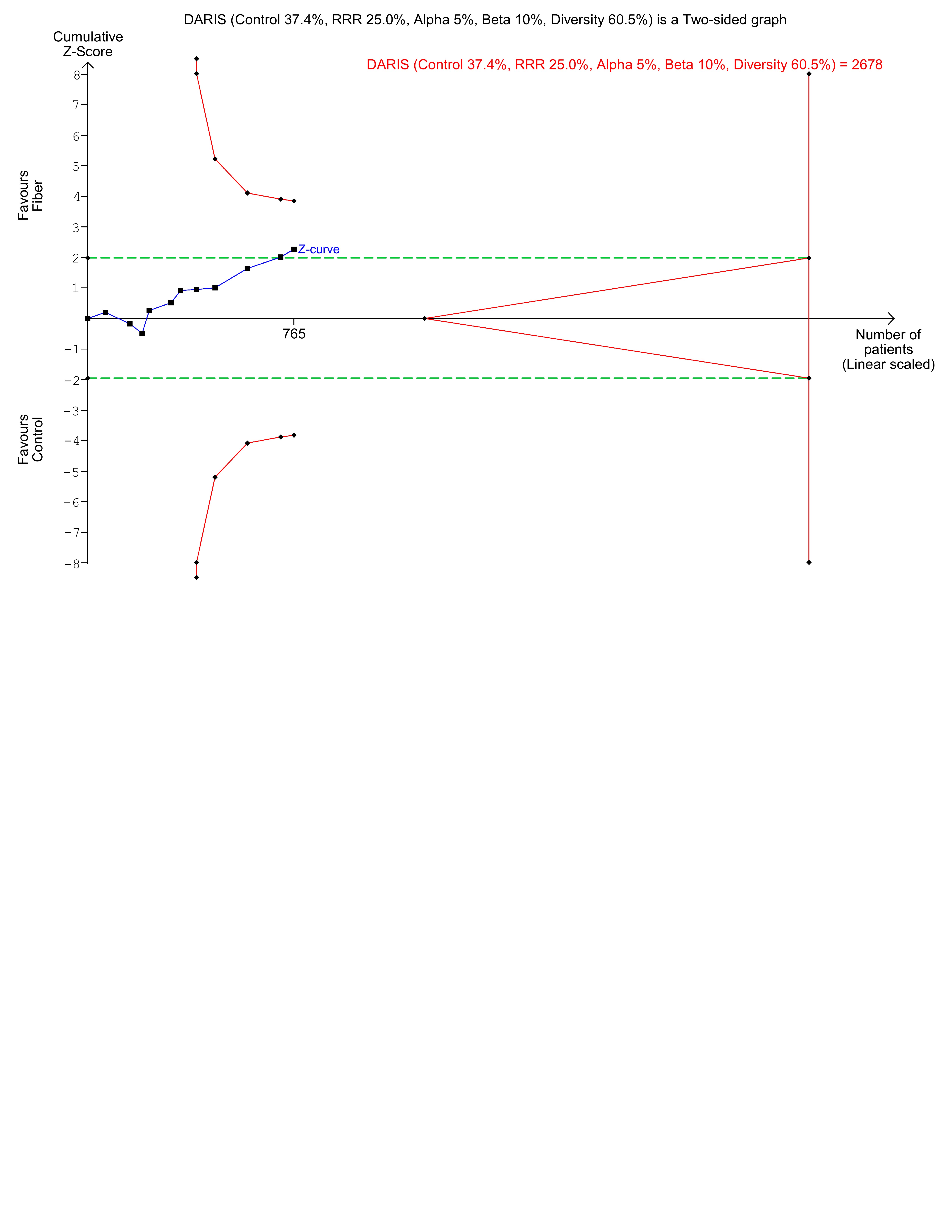


#### b) sensitivity analysis – RRR = 15%


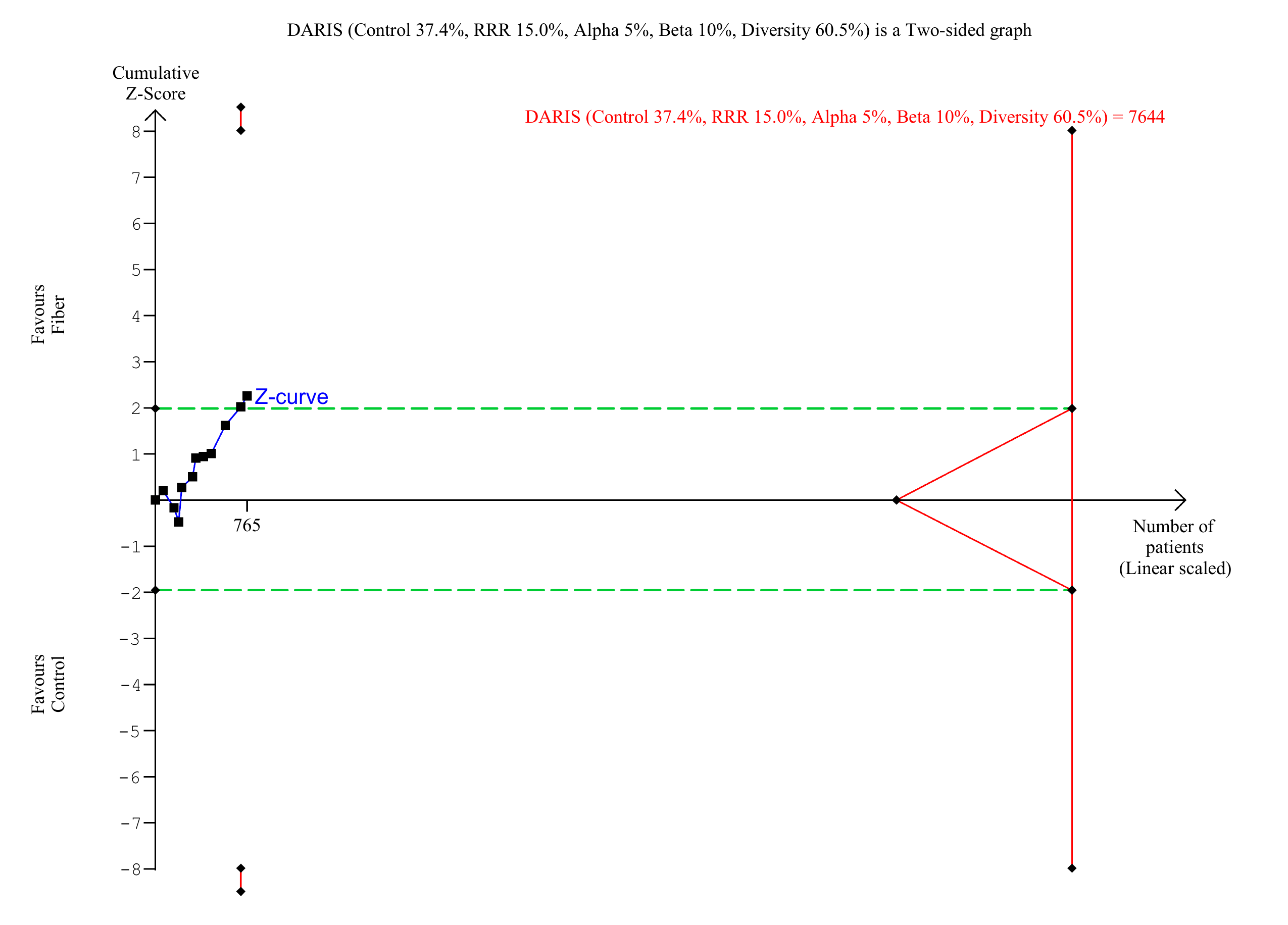


#### c) sensitivity analysis – RRR = 35%


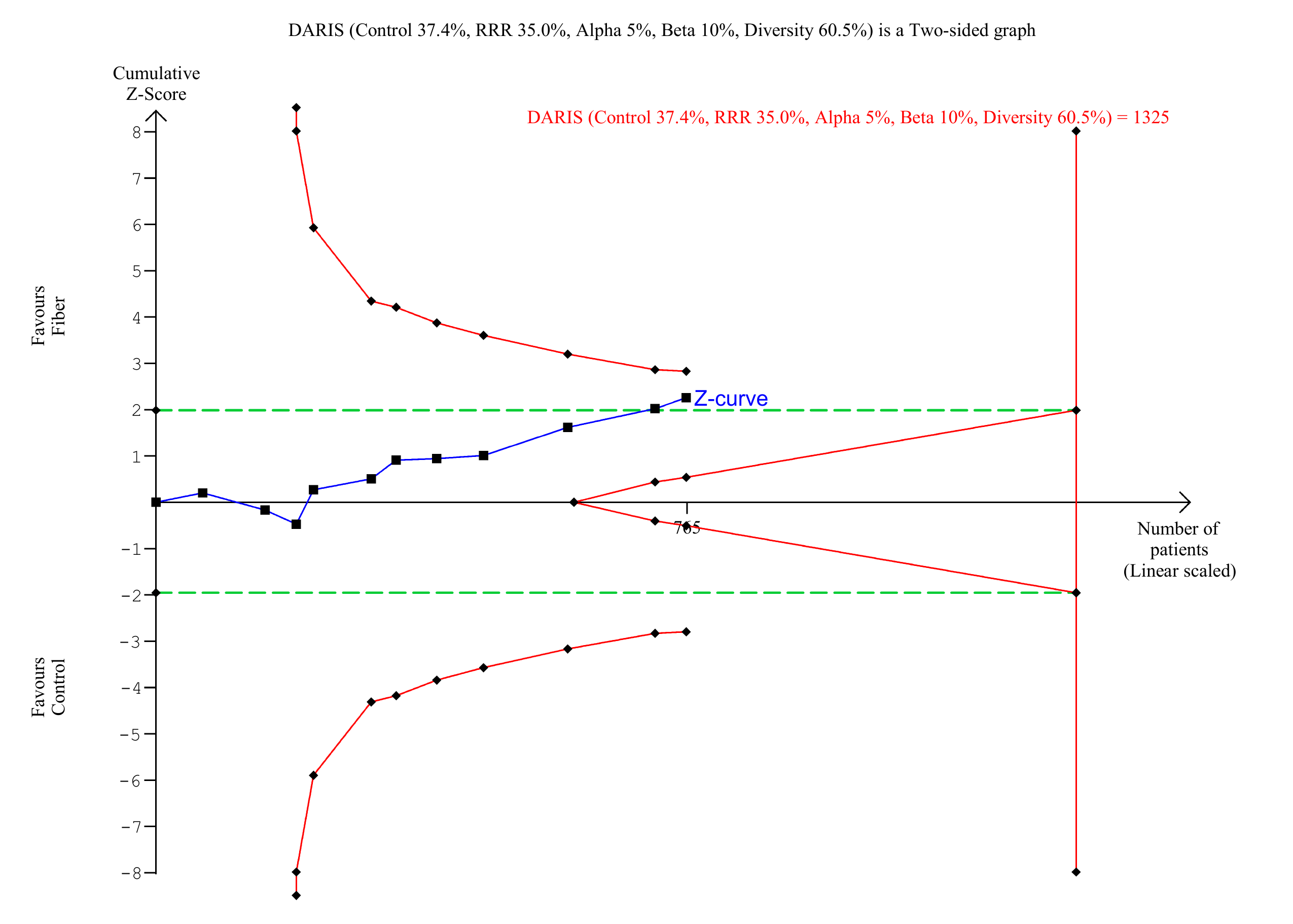


### Fig. S18: ICU LOS with sensitivity analyses

#### a) MIREDIF = 1 day


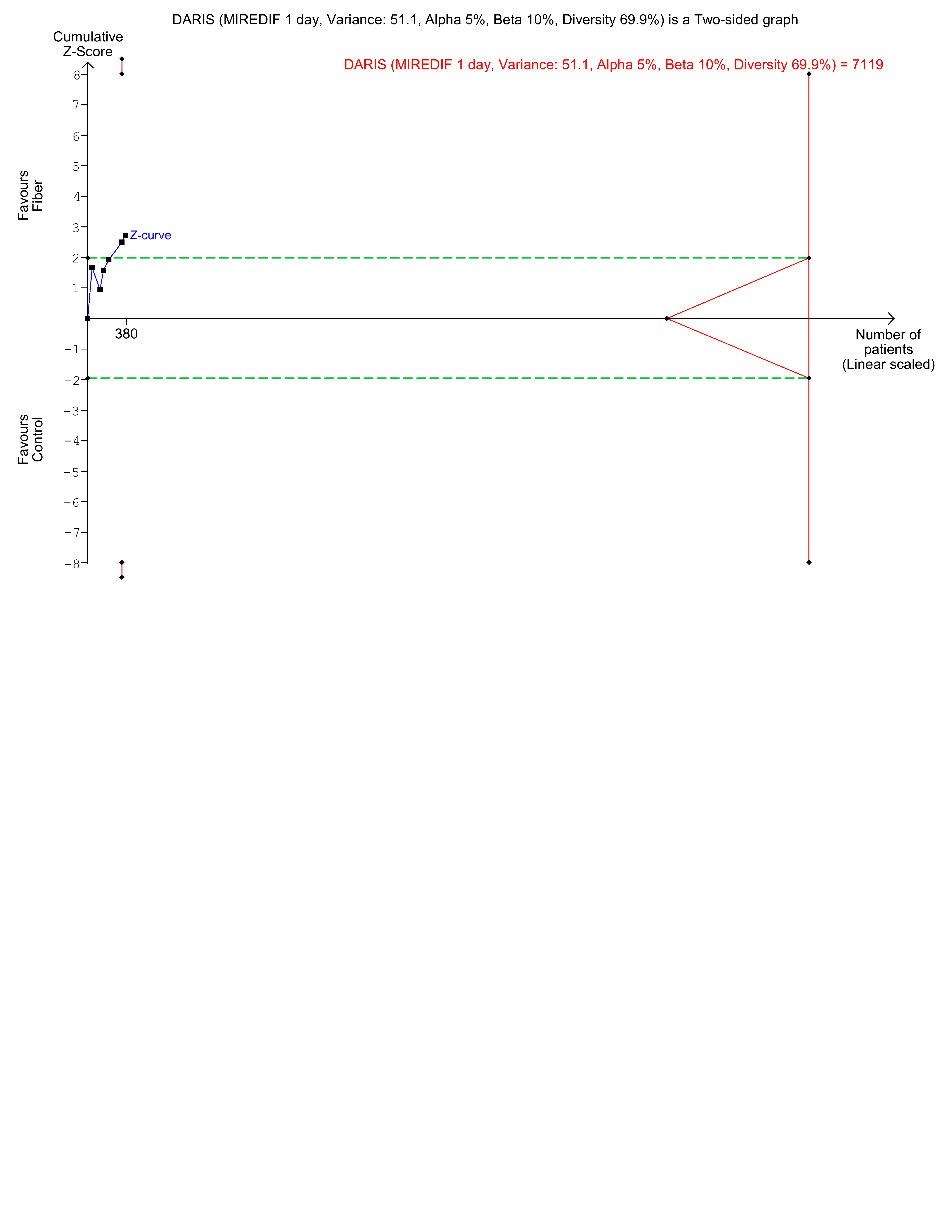


#### b) sensitivity analysis – MIREDIF = 2 days


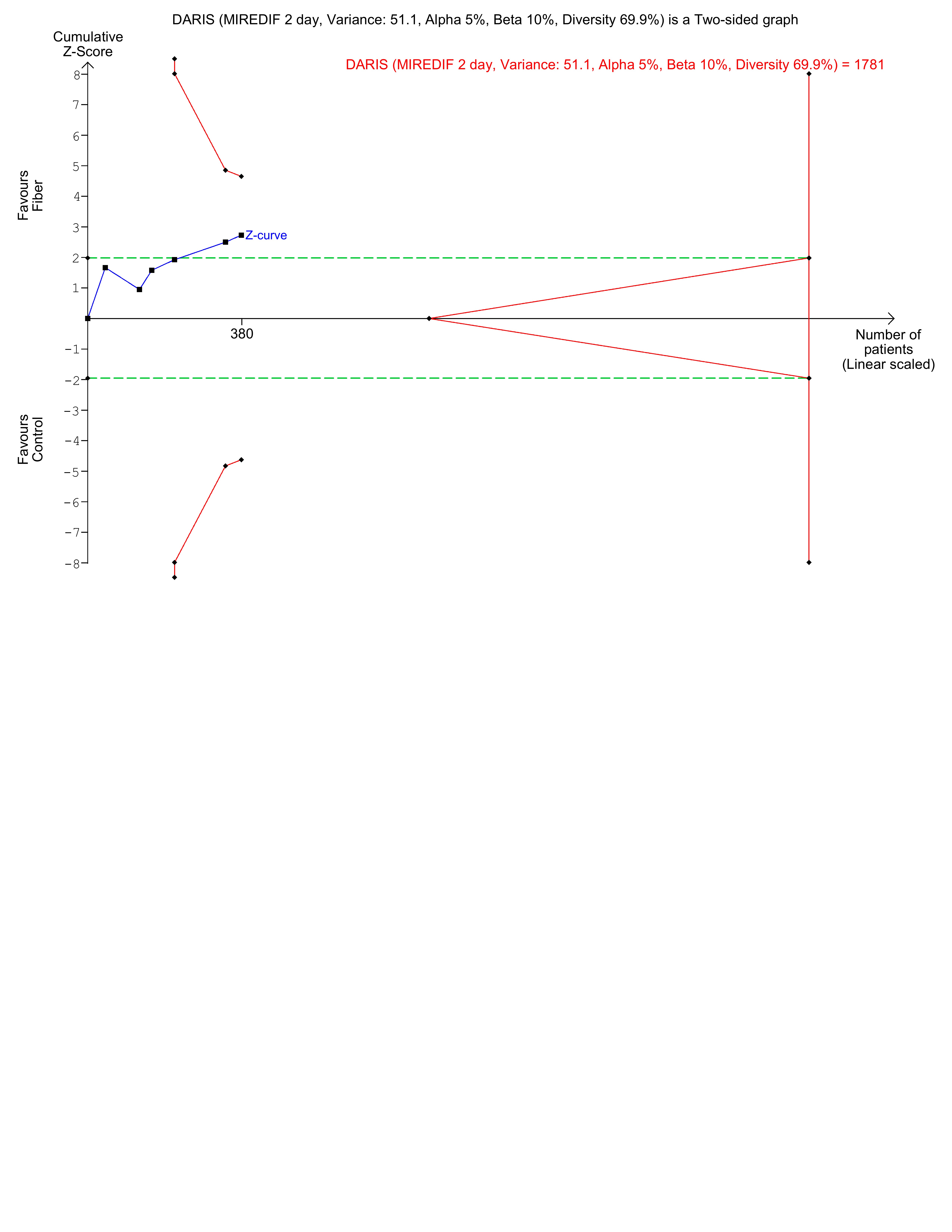


c) sensitivity analysis – MIREDIF = 3 days


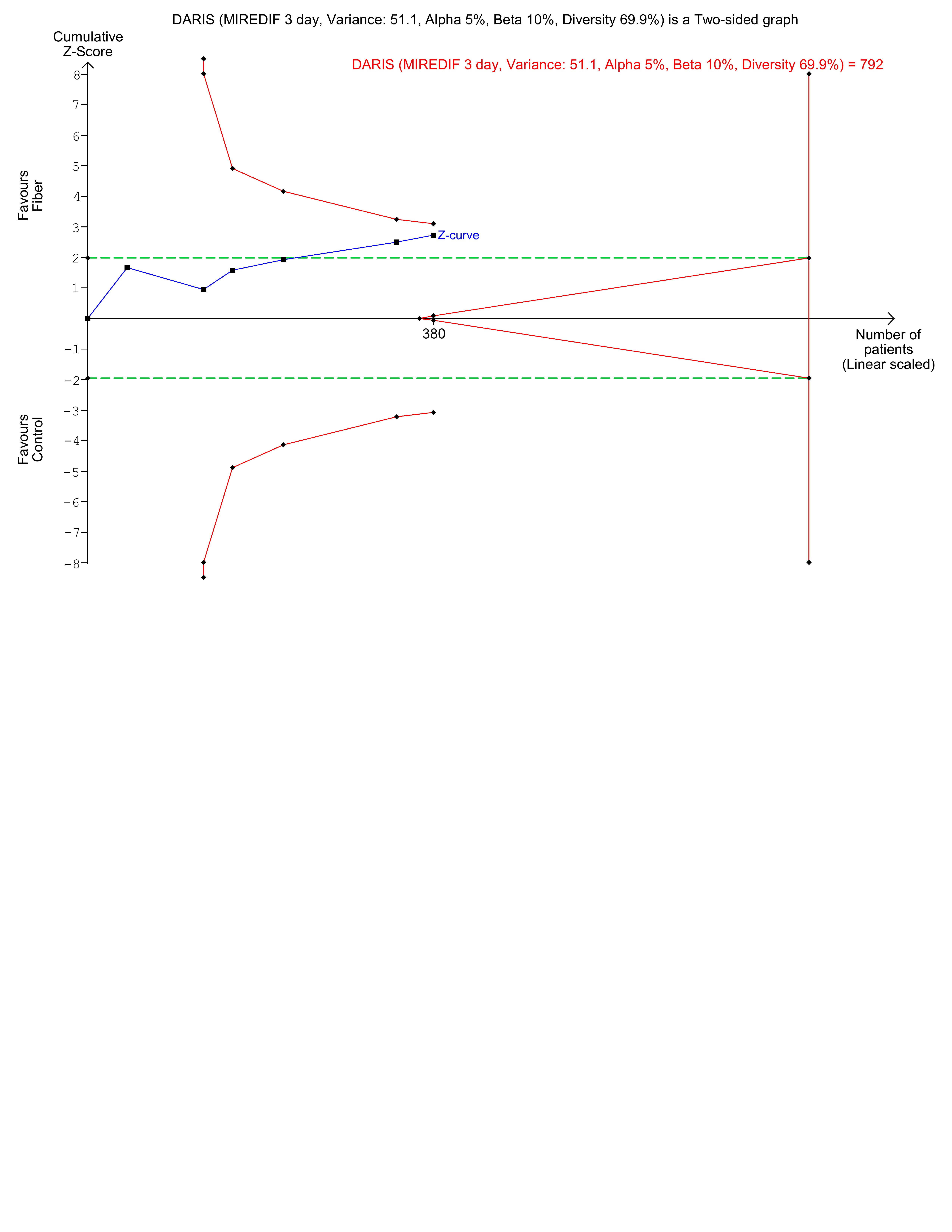


### Fig. S19: Hospital LOS with sensitivity analyses

#### a) MIREDIF = 1 day


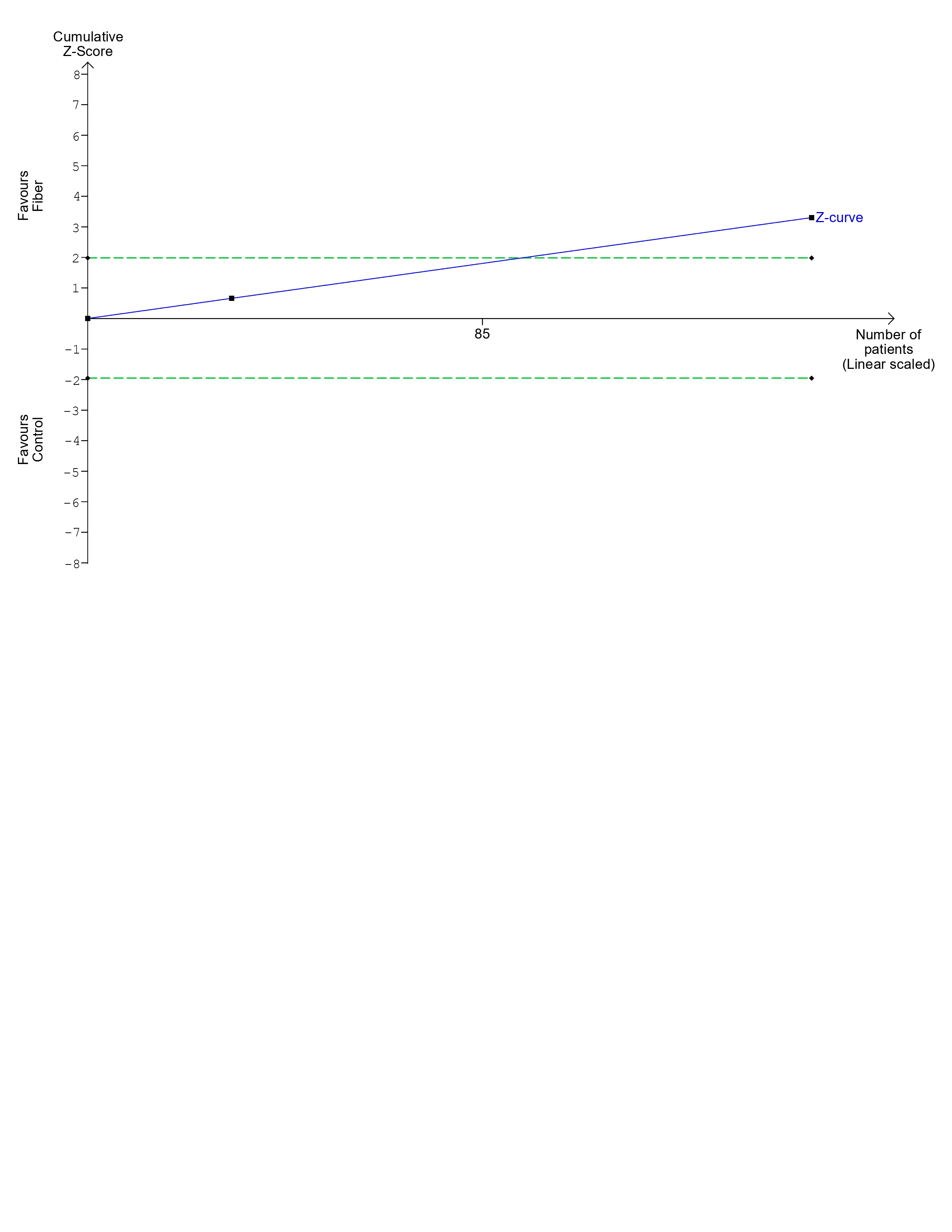


#### b) sensitivity analysis – MIREDIF = 2 days


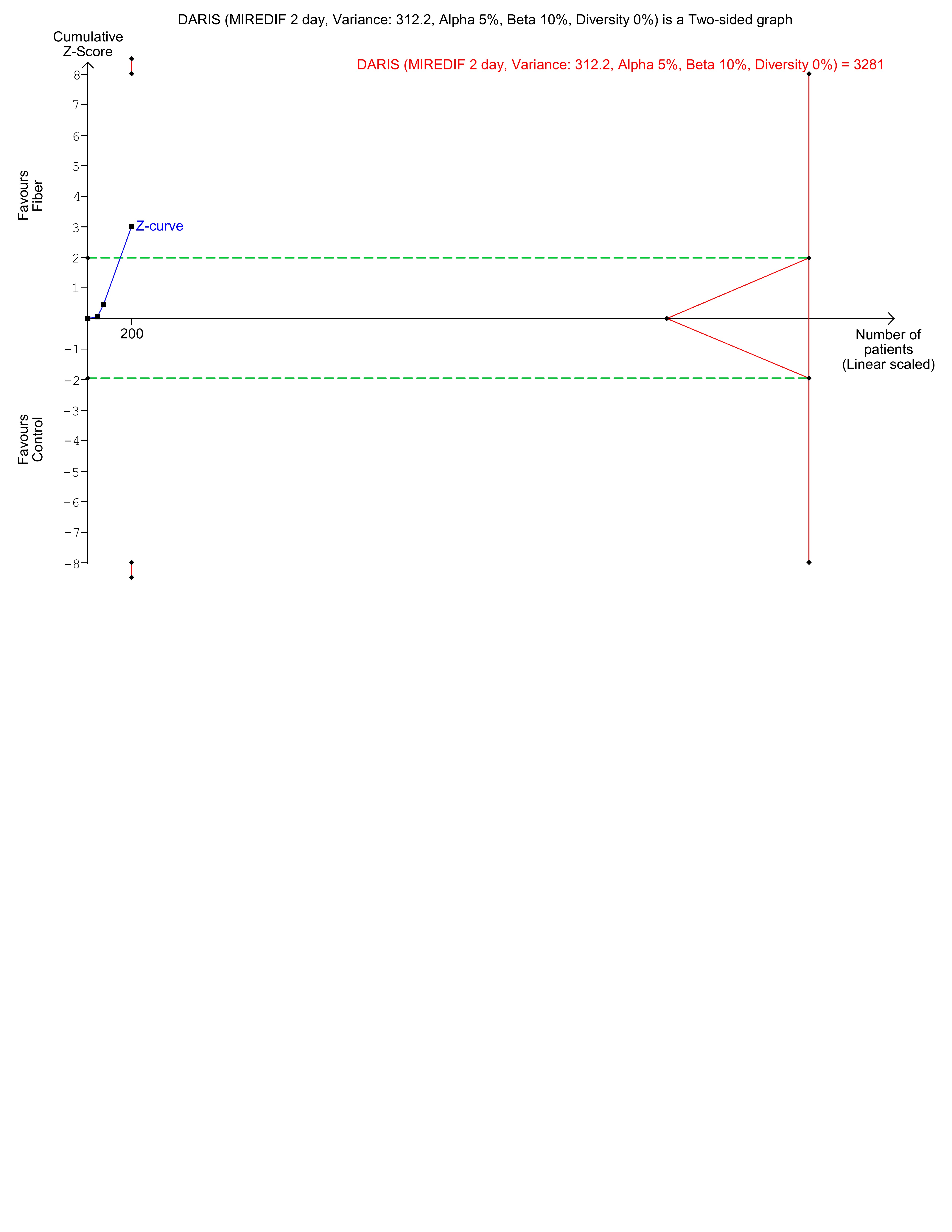


#### c) sensitivity analysis – MIREDIF = 3 days


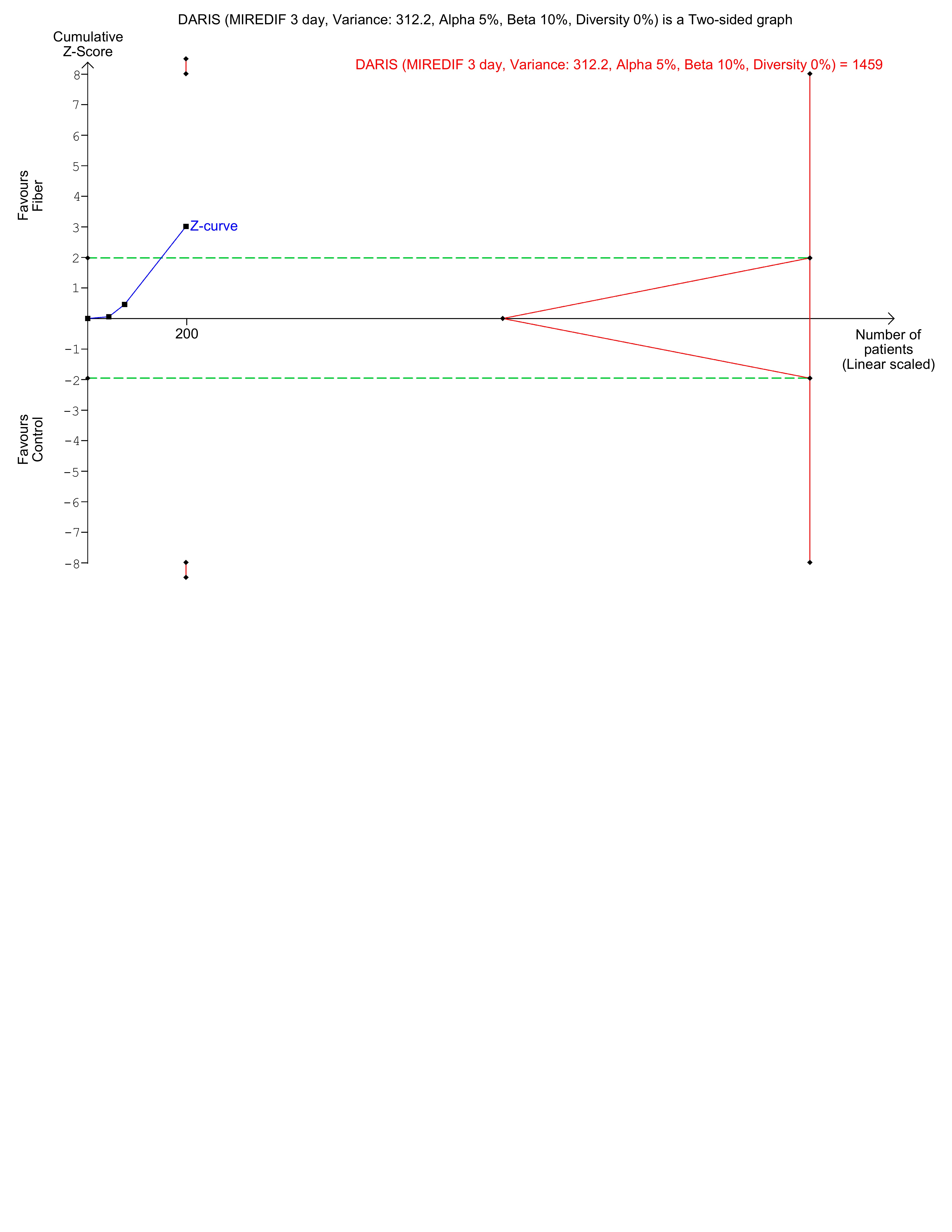


# References

[1] J. Wetterslev, J. C. Jakobsen, and C. Gluud, "Trial Sequential Analysis in systematic reviews with meta-analysis," (in eng), *BMC Med Res Methodol,* vol. 17, no. 1, p. 39, Mar 6 2017, doi: 10.1186/s12874-017-0315-7.
